# Total Synthesis and Structure Assignment of the Relacidine Lipopeptide Antibiotics and Preparation of Analogues with Enhanced Stability

**DOI:** 10.1021/acsinfecdis.3c00043

**Published:** 2023-03-31

**Authors:** Karol Al Ayed, Denise Zamarbide Losada, Nataliia V. Machushynets, Barbara Terlouw, Somayah S. Elsayed, Julian Schill, Vincent Trebosc, Michel Pieren, Marnix H. Medema, Gilles P. van Wezel, Nathaniel I. Martin

**Affiliations:** † Biological Chemistry Group, Institute of Biology, Leiden University, Sylviusweg 72, 2333 BE Leiden, Netherlands; ‡ Molecular Biotechnology Group, Institute of Biology, Leiden University, Sylviusweg 72, 2333 BE Leiden, Netherlands; § Bioinformatics Group, Wageningen University, Droevendaalsesteeg 1, 6708 PB Wageningen, Netherlands; ∥ BioVersys AG, c/o Technologiepark, Hochbergerstrasse 60c, CH-4057 Basel, Switzerland

**Keywords:** relacidine, AMR, lipopeptides, total synthesis, NRPS, bioinformatics

## Abstract

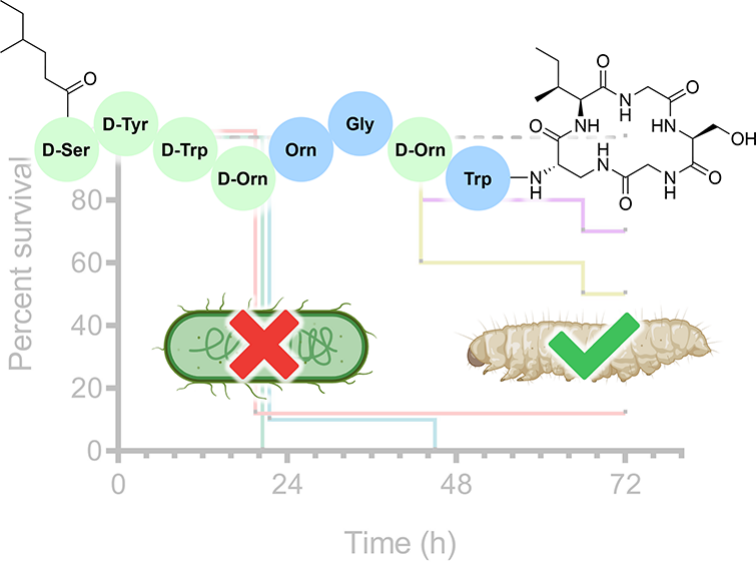

The unabated rise of antibiotic resistance has raised the specter of a post-antibiotic era and underscored the importance of developing new classes of antibiotics. The relacidines are a recently discovered group of nonribosomal lipopeptide antibiotics that show promising activity against Gram-negative pathogens and share structural similarities with brevicidine and laterocidine. While the first reports of the relacidines indicated that they possess a C-terminal five-amino acid macrolactone, an N-terminal lipid tail, and an overall positive charge, no stereochemical configuration was assigned, thereby precluding a full structure determination. To address this issue, we here report a bioinformatics guided total synthesis of relacidine A and B and show that the authentic natural products match our predicted and synthesized structures. Following on this, we also synthesized an analogue of relacidine A wherein the ester linkage of the macrolactone was replaced by the corresponding amide. This analogue was found to possess enhanced hydrolytic stability while maintaining the antibacterial activity of the natural product in both *in vitro* and *in vivo* efficacy studies.

Antimicrobial resistance (AMR) is recognized as a serious threat to public health today and has been projected to become a major global health crisis in the near future. A recently published study estimated that in 2019 there were 4.95 million deaths associated with bacterial AMR, including 1.27 million deaths directly attributable to AMR.^1^ Beyond these current numbers, some have estimated that AMR could kill upward of 10 million people per year globally by 2050.^2,3^ Moreover, a recent report from the US Centers for Disease Control and Prevention found that the COVID-19 pandemic has further accelerated the spread of AMR, particularly in hospital settings.^4^ In assessing the threat posed by AMR, the so-called ESKAPE pathogens have risen to prominence, which comprise *Enterococcus faecium*, *Staphylococcus aureus*, *Klebsiella pneumoniae*, *Acinetobacter baumannii*, *Pseudomonas aeruginosa*, and the *Enterobacter* species.^5^ These pathogens are prone to developing antibiotic resistance, and the infections they cause are often difficult to treat. Notable among the ESKAPE pathogens are the Gram-negative species, which the World Health Organization has exclusively categorized as threat-level “critical.” While there is a dire need for antibacterial agents that can effectively combat these particularly dangerous Gram-negative bacteria, the development of new antibiotics has stagnated since the 1980s.^6^ Clearly, innovative approaches are needed to discover and develop new antibiotics that act against these pathogens.

In 2018, Qian and co-workers reported two new lipopeptide antibiotics discovered by mining bacterial genomes.^7^ These natural products, termed brevicidine and laterocidine (Figure 1), were found to exhibit potent anti-Gram-negative specific activity and low eukaryotic cell toxicity. The low yields associated with the isolation of brevicidine and laterocidine via fermentative methods prompted us to pursue a total synthesis approach for their production, which we also recently reported.^8^ Shortly after brevicidine and laterocidine were discovered, the Kuipers group reported a series of structurally similar lipopeptides they named the relacidines.^9^ As with brevicidine and laterocidine, the relacidines were found to specifically affect the growth of Gram-negative bacteria via a mechanism involving interactions with lipopolysaccharide (LPS).^9^ On the basis of the relacidine biosynthetic gene clusters and subsequent NMR and mass-spectrometric analysis of the natural products isolated, the Kuipers group proposed two main relacidine structures: relacidine A and relacidine B (Figure 1).

**Figure 1:**
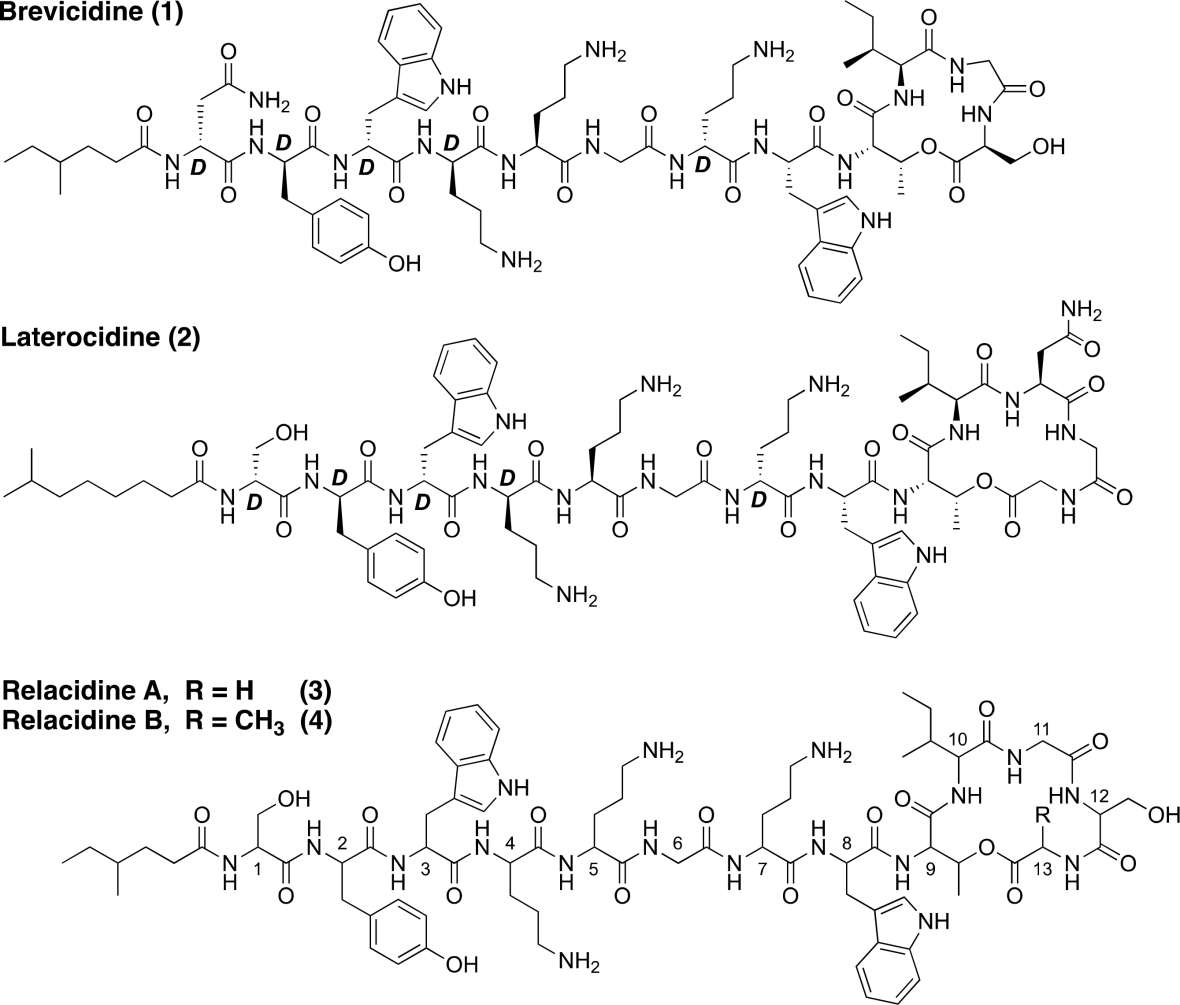
Structures of brevicidine (**1**), laterocidine (**2**), and previously proposed structures for relacidine A (**3**) and B (**4**). For the structures of brevicidine and laterocidine, d-amino acids are labeled d. Amino acid numbering is indicated for relacidine A and B.

While the stereochemical configurations of the amino acids comprising relacidine A and B were not assigned in the original report, the compounds are clearly similar in structure to brevicidine and laterocidine. In terms of their primary sequences, relacidine A and B differ only in the C-terminal residue that is esterified with the side chain of Thr9 to form the macrolactone. In relacidine A this C-terminal residue is Gly, while in relacidine B it is Ala (Figure 1). In comparison of the relacidines to brevicidine and laterocidine, it is apparent that they more closely resemble laterocidine. The sequence of the exocyclic linear peptide is the same for the relacidines and laterocidine and they also both have a five-amino acid macrocycle, while the brevicidine macrocycle consists of four residues. It is in the amino acids of the macrocycle that the relacidines differ from laterocidine. Specifically, the three C-terminal residues in the relacidines are Gly11, Ser12, and Gly13/Ala13, while in laterocidine they are Asn11, Gly12, and Gly13. These differences, and the lack of stereochemical assignments, prompted us to consider a total synthesis approach to the relacidines as a means of unambiguously establishing their structures. Furthermore, as for brevicidine and laterocidine, isolation of the relacidines from fermentation of the producing organism, *Brevibacillus laterosporus*, requires laborious purification while yielding low quantities of pure material (submilligram per liter). In such cases, total synthesis can provide an attractive alternative for obtaining quantities of material suitable for more comprehensive studies. To this end, we here report the total synthesis of a series of relacidine A and B diastereomers that, when compared with the natural product, allowed for unambiguous stereochemical assignments. In addition, we describe the synthesis of an analogue of relacidine A that exhibits increased stability in serum while retaining potent *in vitro* activity against a number of Gram-negative pathogens and *in vivo* activity in an established *Galleria mellonella* larvae infection model.

Prior to embarking on the synthesis of relacidine A and B, we used a bioinformatics-based approach to predict the stereochemical configuration of the amino acids. To this end, we first analyzed the genome of *Brevibacillus laterosporus* MG64 (GenBank accession NZ_QJJD01000001), the producing strain originally characterized by Kuipers and co-workers, using antiSMASH (v6.0.0; default settings).^9−11^ This allowed us to readily identify the relacidine biosynthetic gene cluster (BGC, see Supporting Information) on the basis of biosynthetic logic. Subsequently, the architecture of the nonribosomal peptide synthetase (NRPS) modules that assemble the relacidine peptide scaffold were studied. Particularly, we determined which modules contain epimerization domains, i.e., domains that catalyze the conversion from an l- to d-amino acid. Modules 1, 2, 3, 4, and 7 of *rlcC*, which incorporate serine, tyrosine, tryptophan, ornithine, and ornithine, respectively, were all predicted to contain epimerization domains (Figure 2A). Therefore, these residues were predicted to have a d-configuration in the final relacidine scaffold. To further confirm this, the relacidine BGC was compared with those of brevicidine and laterocidine using the MIBiG database (MIBiG accessions BGC0001536 and BGC0002432, respectively), which showed that, barring a missing terminal module in the brevicidine BGC, the module architectures of all three BGCs are identical.^12^ Because the stereochemical configurations of brevicidine and laterocidine have been fully assigned, we could confirm that the epimerization domains in the brevicidine and laterocidine BGCs correspond exactly to the positions of the d-amino acids observed in their respective products.^7^ Therefore, we predicted that the relacidines likely have the same stereochemical configurations at the same amino acid positions compared with brevicidine and laterocidine (Figure 2B).

**Figure 2:**
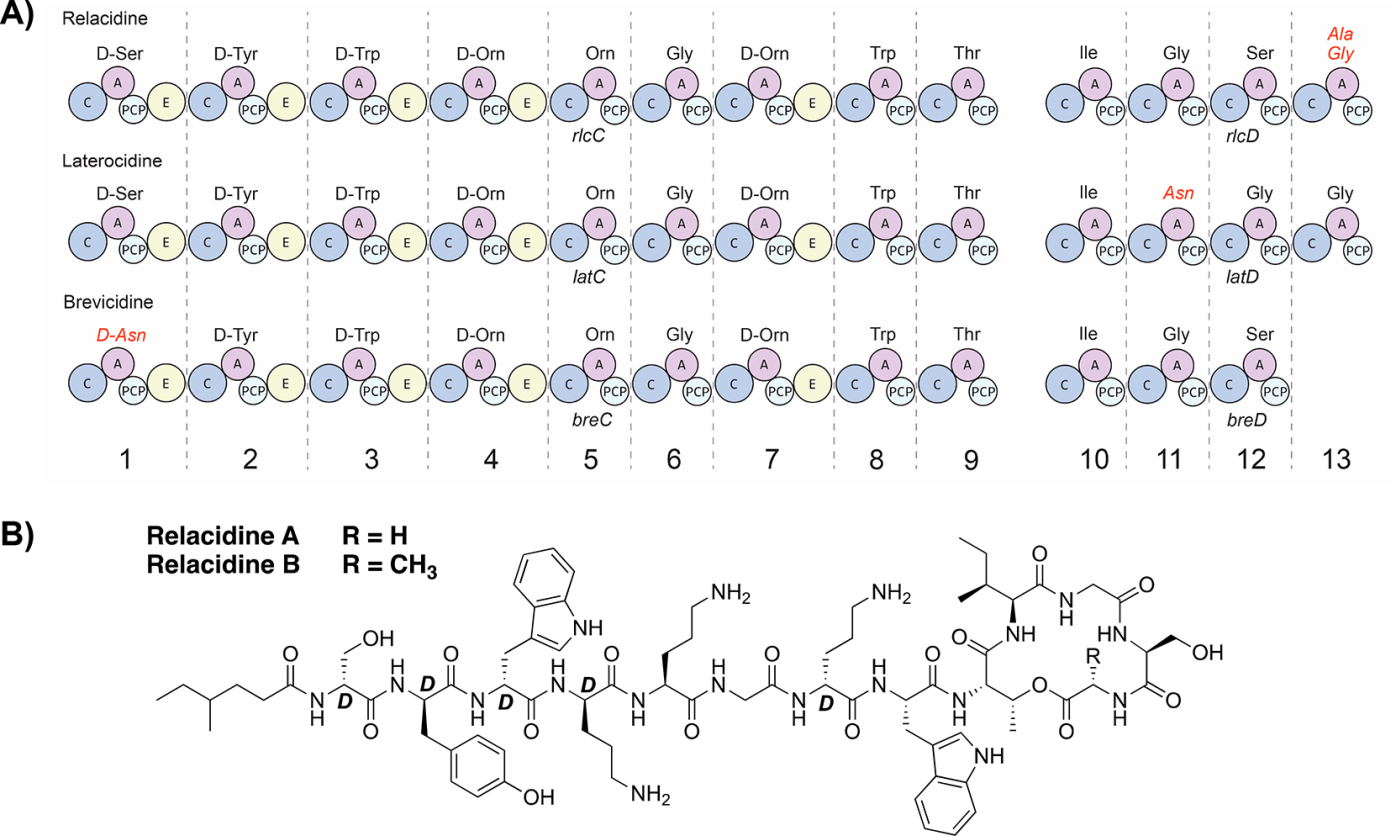
Comparison of the relacidine, laterocidine, and brevicidine biosynthetic gene clusters. (A) Architecture of the NRPS modules of the relacidine, laterocidine, and brevicidine BGCs. (B) Predicted chemical structures of relacidine A and B, including stereochemical assignments. (C = condensation domain; A = adenylation domain; PCP = peptidyl carrier protein domain; E = epimerization domain). d-amino acids are labeled d.

The peptide backbones of the relacidines, brevicidine, and laterocidine show differences at various positions (Figure 2A). We analyzed the genetic basis for these differences by examining the active site sequences of the adenylation (A) domains, which are responsible for selecting the amino acid building blocks in nonribosomal peptide biosynthesis. We uncovered a curious difference between the relacidine and laterocidine BGCs in the fourth module of *rlcD* (*latD*), which incorporates Gly in laterocidine biosynthesis and either Ala or Gly in relacidine biosynthesis.

Specifically, the relacidine BGC encodes Tyr at position 290, while the laterocidine BGC encodes Trp at the same position (labeled as position 291 in the laterocidine BGC). This difference is notable because this residue is centrally located in the active site of the A-domain that incorporates the 13th amino acid of relacidine and laterocidine (Figure 3A). Given that the only difference between relacidine A and B is the incorporation of Gly or Ala, respectively, as the 13th amino acid, we asked whether this could be due to substrate promiscuity of the A-domain related to the presence of Tyr rather than Trp at position 290 of the active site. This was further suggested by sequence analysis, which revealed that the active sites of all other Gly-only activating A-domains in relacidine, laterocidine, and brevicidine (Figure 3A) also contain a Trp rather than a Tyr residue at position 290. To investigate this further, the 3D protein structures of all Gly-recognizing A-domains encoded by the relacidine, laterocidine, and brevicidine BGCs were first predicted with AlphaFold2. We next investigated how the Gly or Ala substrates, corresponding to relacidine A and B respectively, would fit into the A-domain active sites.^13^ On the basis of this analysis, we hypothesize that in domains that exclusively incorporate Gly, Trp serves as a “gatekeeper” by effectively limiting the size of the active site pocket so that only the Gly substrate can fit. When modeling the corresponding Ala substrate into the active sites, the predicted distance between the Trp side chain of Gly-exclusive domains and the Ala methyl group is 1.9–2.4 Å, which would likely cause steric repulsion (Figure 3B; Supplemental Table S1). In contrast, if the A-domain active site contains the smaller Tyr residue at position 290 (as observed for the 13th A-domain encoded by the relacidine BGC), it appears to tolerate recognition of the Ala substrate, with a predicted distance of 3.4 Å between the Tyr side chain and the Ala methyl group. These findings suggest that a single substitution in the active site of an A-domain can lead to substrate promiscuity, thereby resulting in the production of a NRPS with varying structural features encoded by the same BGC.

**Figure 3:**
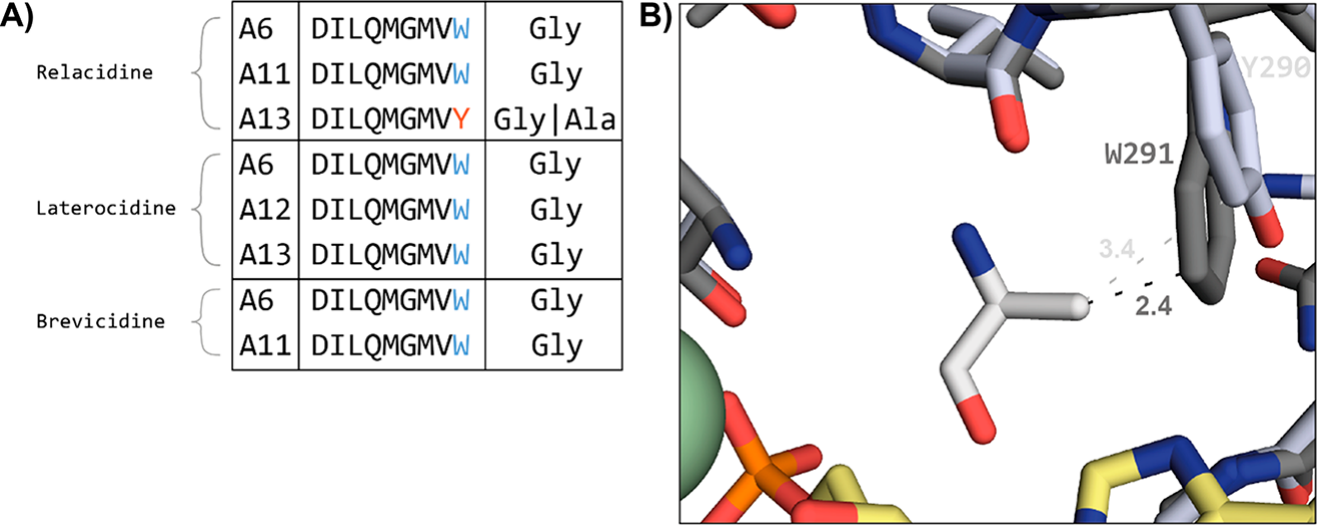
Comparison of the Gly-recognizing A-domains in the relacidine, laterocidine, and brevicidine biosynthetic gene clusters. (A) Comparison of the active sites of the Gly-recognizing A-domains; (B) Comparison of the 3D structures predicted for the active sites of A-domain 13 containing Trp (laterocidine BGC; dark gray) or Tyr (relacidine BGC; gray), which shows interactions of these residues with the Ala substrate.

On the basis of the stereochemical predictions generated from our analysis of the relacidine BGC, we next set out to synthesize relacidine A and B. The approach used was inspired by our earlier work on the total synthesis of brevicidine and laterocidine with some key differences.^8,14^ Specifically, our previous solid-phase peptide synthesis (SPPS)-based preparation of laterocidine made use of the fact that the laterocidine macrocycle includes an Asn residue at position 11, which presented a viable option for resin attachment. However, because the relacidines lack an Asn residue at position 11, a different resin anchoring strategy was required. Instead, we identified the Ser at position 12 of the relacidine macrocycle as a viable option for resin attachment. To this end, the free side chain hydroxyl of Fmoc-l-Ser-OAllyl was first attached to 2-chlorotrityl resin (2-CT) via an overnight coupling, which resulted in an acceptable loading of 0.37 mmol/g. The peptide was then extended to the pentapeptide using standard SPPS with the notable incorporation of Thr9 as the side chain unprotected species (Scheme 1A). At this stage, the required ester linkage between the Thr9 side chain hydroxyl and the C-terminal carboxylate of Gly13 was introduced by coupling Alloc-Gly using an on-resin Steglich esterification. After the subsequent simultaneous removal of the allyl and Alloc protecting groups, the macrocycle was formed using a BOP/DIPEA-mediated amide bond formation between Ser12 and Gly13. The remaining exocyclic peptide was then elongated through eight additional rounds of SPPS, including an N-terminal lipidation using racemic 4-methylhexanoic acid. Cleavage of the peptide from resin with concomitant global deprotection was achieved using acidic conditions, after which RP-HPLC purification afforded the desired relacidine A diastereomer **3a**. The same route was then applied to the synthesis of relacidine B diastereomer **4a** with the only difference being that Alloc-l-Ala was instead used to install the ester linkage with Thr9. Given that the relacidines differ from laterocidine only at positions 12 and 13, we also opted to prepare the full suite of possible relacidine A and B diastereomers on the basis of l/d-Ser at position 12 and Gly or l/d-Ala at position 13 (Scheme 1B).

**Scheme 1:**
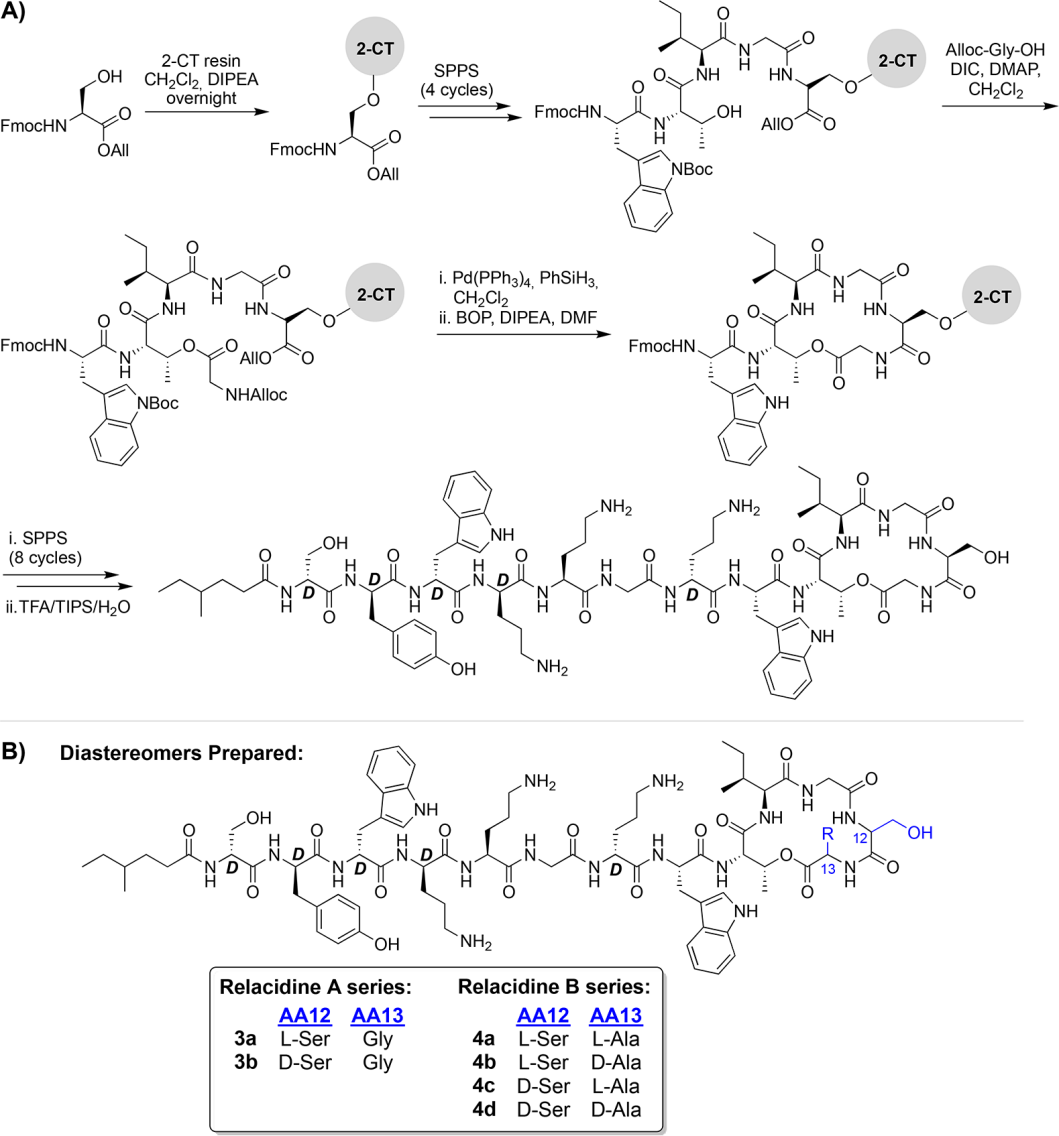
(A) Representative Total SPPS of Relacidine A (**3a**) and (B) Structures of Various Diastereomers of Relacidine A and B Also Prepared

With access to relacidine A diastereomers **3a,b** and relacidine B diastereomers **4a**–**d** via chemical synthesis, we next set out to confirm the stereochemical configuration of the natural products by comparison with the relacidines obtained from fermentation of the producing strain. To do so, the relacidines were extracted from the cellular fraction of a *B. laterosporus* MG64 culture fermented in LB broth for 24 h. The crude extract thus obtained was analyzed using liquid chromatography–mass spectrometry (LC-MS) in order to compare the retention times of the natural and synthetic relacidines. Coelution studies clearly demonstrated that the retention times of relacidine A and B from the bacterial extracts matched compounds **3a** and **4a**, respectively (Figure 4). These findings support an l-configuration for Ser12 in relacidine A and B and an l-configuration for Ala13 in relacidine B, in accordance with the predictions on the basis of our assessment of the BGC. Furthermore, analysis of the ^1^H NMR spectra of relacidine B diastereomers **4a**–**4d** (Supplemental Figure S1) clearly show that the spectra obtained for diastereomer **4a** match the data previously published for relacidine B^9^ (note: because of the extremely low isolated yield of relacidine A from fermentation, no NMR data were reported for the natural product, which prevented comparison with our synthetic relacidine A diastereomers).

**Figure 4:**
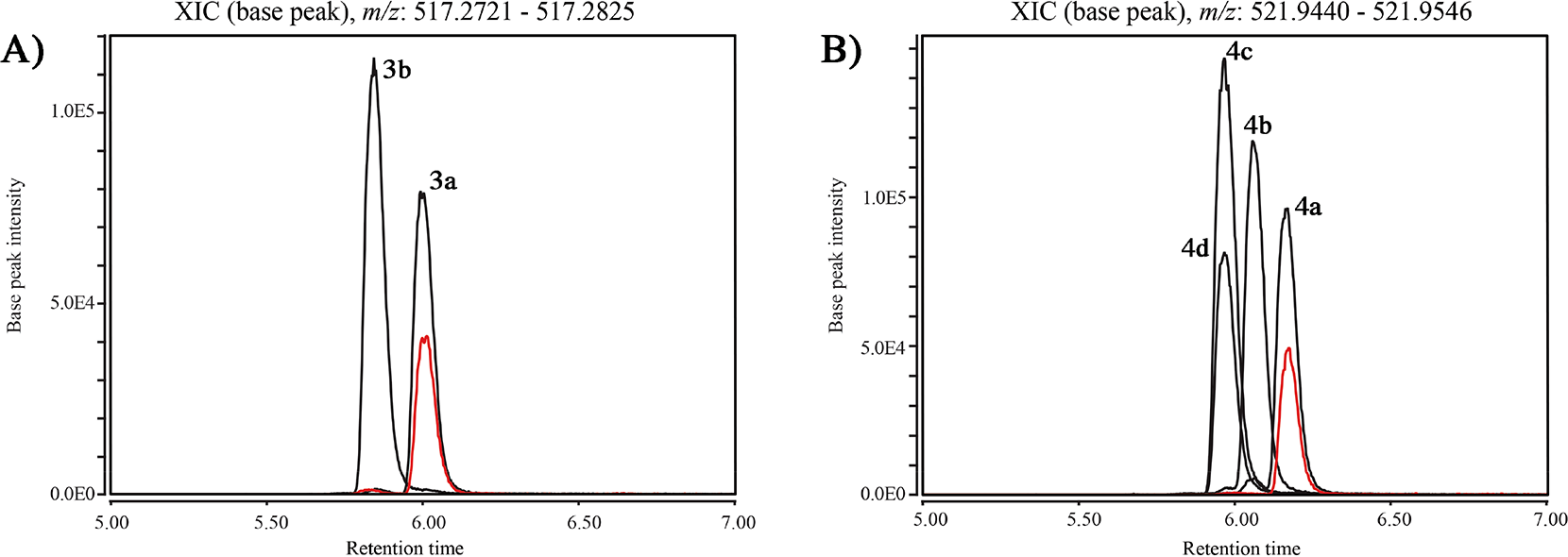
Extracted ion chromatograms (EICs) of (A) relacidine A with *m*/*z* 517.2776 and (B) relacidine B with *m*/*z* 521.9492 from the crude extracts of *B. laterosporus* (red), overlaid with traces obtained for relacidine A diastereomers **3a,b** and relacidine B diastereomers **4a**–**d**. Synthetically prepared **3a** and **4a** coelute with relacidine A and B from *B. laterosporus* MG64, respectively.

With the structures of relacidine A and B established, we next explored the synthesis of a relacidine A analogue wherein the ester linkage of the macrolactone ring was replaced by the corresponding amide. Our interest in this “relacidamide” analogue was 2-fold: we hypothesized, first, that the amide analogue would be more readily synthesized, and second, that the macrolactam ring would be more stable to hydrolysis than the corresponding macrolactone. The synthesis of relacidamide (**5**) is depicted in Scheme 2 and was inspired by our previous work with laterocidine and brevicidine analogues.^8^ SPPS was used to assemble the linear precursor peptide on 2-CT resin with the notable introduction of 3-azido-l-alanine at position 9 instead of Thr. Notably, our initial approach involved the incorporation of Alloc-l-diaminopropionic acid at position 9; however, the required on-resin deprotection of the Alloc group following completion of the linear precursor peptide was found to proceed very sluggishly. By comparison, reduction of the azide in the 3-azido-l-alanine-containing peptide proceeded smoothly upon treatment of PMe_3_, which afforded the amine cleanly after 3 h. The protected linear peptide was then cleaved from the resin using mild acidic condition (HFIP/DCM) and cyclized in solution using BOP/DIPEA. Finally, global deprotection and purification using RP-HPLC afforded pure relacidamide (**5**) in 18% yield over 30 steps.

**Scheme 2:**
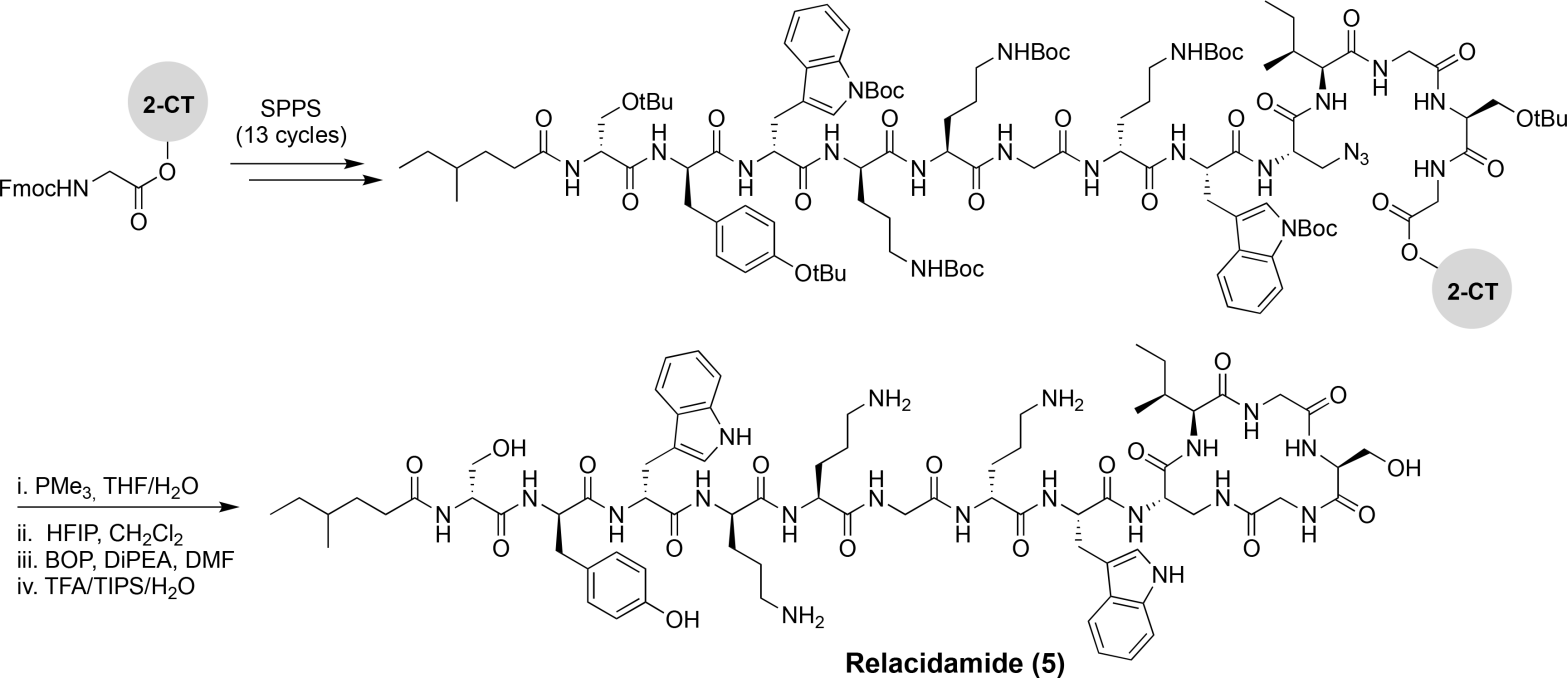
Synthesis of Relacidamide (**5**)

The antibacterial activities of relacidine A (**3a**) and B (**4a**), along with diastereomers **3b** and **4b**–**d** and relacidamide (**5**), were assessed using a standard microbroth dilution assay to establish minimum inhibitory concentration (MIC) values against a panel of Gram-negative bacteria (Table 1). The MIC values measured for synthetic relacidine A (**3a**) and B (**4a**) agree well with those previously published.^9^ Interestingly, the unnatural diastereomers of relacidine A and B, as well as the relacidamide analogue (**5**), exhibited antibacterial activities similar to the natural products. As in the case of brevicidine and laterocidine, the biological activity of the relacidines was found to be unaffected by the clinically relevant *mcr-1* type colistin resistance. Relacidine A (**3a**) and B (**4a**), as well as relacidamide (**5**), were further tested against a panel of colistin-resistant *A. baumannii* clinical isolates, which confirmed their ability to prevent the growth of colistin-resistant isolates (Supplemental Table S2). The hemolytic activity of the compounds was also assessed, which showed them to cause little-to-no hemolysis when tested at 128 μg/mL (Supplemental Figure S2). Notably, while relacidine A (**3a**) showed the highest level of hemolysis at 15% under these conditions, relacidamide analogue (**5**) showed essentially no hemolysis (1%). Also of interest was the finding that while relacidine B (**4a**) induced <1% hemolysis, a substitution of either the l-Ala or l-Ser to d-Ala or d-Ser increased hemolytic activity to 8–12%. Serum stability assays were also performed, thereby revealing relacidamide (**5**) to be significantly more stable than relacidine A (**3a**), which was in line with expectation (Supplemental Figure S3). LC-MS analysis indicated that the most prominent degradation product of relacidine A corresponds to the hydrolyzed species (+18 amu), presumably because of the cleavage of the ester linkage between Thr9 and Gly13. Somewhat surprisingly, relacidine B (**4a**) was also found to be highly resistant to degradation in human serum even after 24 h of incubation. The greater hydrolytic stability of relacidine B would appear to be attributable to the increased steric bulk of the Ala side chain next to the ester linkage (compared with the methylene of Gly in relacidine A), which may hinder the nucleophilic attack of water at the ester carbonyl.

**Table 1:** In Vitro Minimum Inhibitory Concentrations (MICs) of Relacidines

	peptide	*E. coli* ATCC 25922	*E. coli* ATCC 25922 *mcr-1*	*K. pneumoniae* ATCC 13883	*P. aeruginosa* PAO1	*S. aureus* USA300
peptide MIC^a^	**3a**	2	2–4	4	4	>32
	**3b**	2–4	4	4	2	>32
	**4a**	1	2	4	4	>32
	**4b**	2	2–4	4	4	>32
	**4c**	1–2	1–2	2–4	4	>32
	**4d**	2	2–4	2–4	8	>32
	**5**	2–4	2–4	4	8	>32
	colistin	1	4	0.25–0.5	1	>32

a MICs given in μg/mL.

Given the promising *in vitro* activity of relacidamide (**5**), along with its high serum stability, low hemolytic activity, and ease of synthesis, we further evaluated its efficacy in an established *in vivo* infection model using *G. mellonella* larvae. The *G. mellonella* infection model has gained popularity in recent years and provides a convenient method to assess the *in vivo* effectiveness and toxicity of novel antibiotics.^15,16^ Relacidamide (**5**) was found to be well tolerated up to the highest tested dose, after which its ability to treat infection with a colistin-resistant *A. baumannii* isolate (strain no. BV94) was assessed (Figure 5). The larvae were first injected with either vehicle (PBS) or *A. baumannii* BV94 suspension in the right second proleg. The moth larvae were then injected in the left second proleg with either PBS (untreated), colistin at 100 mg/kg or 200 mg/kg, or relacidamide (**5**) at 100 mg/kg or 200 mg/kg. The *G. mellonella* larvae were then incubated for 72 h, and their survival was assessed twice per day. Relacidamide (**5**) induced 50% survival after 72 h at 100 mg/kg and 70% survival at 200 mg/kg, whereas none of the larvae treated with colistin survived, and only 10% of the untreated larvae survived after 72 h. These results also reflect the differences observed for the *in vitro* activity of relacidamide (**5**), which showed a MIC of 8 μg/mL against *A. baumannii* BV94, while colistin showed no activity at the highest concentration tested of 64 μg/mL (Supplemental Table S2).

**Figure 5:**
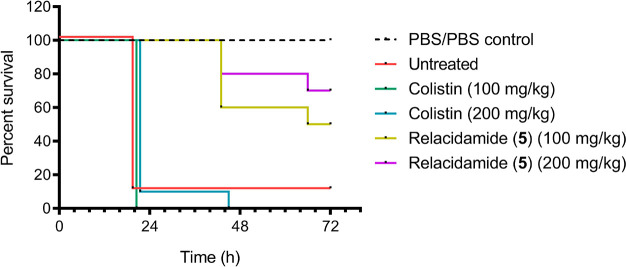
Percent survival of *G. mellonella* larvae after infection with colistin resistant *A. baumannii* isolate BV94 and subsequent treatment with test articles.

In conclusion, we here report the total synthesis of the recently discovered lipodepsipeptide antibiotics relacidine A and B, which was guided by bioinformatic analysis of the BGC. By examining the positions of the epimerization domains encoded by the relacidine BGC, we were able to predict the structure of these natural products, including the stereochemical assignments of their component amino acids. Also of note, our analysis suggests that a single Trp to Tyr mutation in the active site of A-domain 13 of RlcD may allow the incorporation of Ala at position 13 in relacidine B. Following on this, we developed a robust SPPS route that provided access to relacidine A and B, including a number of diastereomers. Subsequent comparison of the synthetic lipopeptides with the natural products confirmed the structural predictions on the basis of our analysis of the relacidine BGC. Furthermore, comparison of the NMR data published for relacidine B with the data obtained for the synthetic relacidine B diastereomers confirmed the predicted stereochemical configuration of relacidine B, thereby validating our bioinformatics predictions. The antibacterial activities of the synthetic relacidines prepared, including the amide-for-ester relacidamide analogue, were evaluated against a panel of Gram-negative bacteria. All of the relacidines exhibited similar antibacterial potency while also exhibiting low hemolytic activity. In line with expectation, the relacidamide analogue (**5**) exhibited high serum stability while retaining potent activity *in vitro*, and was, therefore, further evaluated *in vivo* in a *G. mellonella* larvae infection model. The compound was well tolerated at the highest dose tested and exhibited efficacy against a colistin-resistant *A. baumannii* isolate. These results showcase the relacidines as promising new lipopeptide antibiotics and indicate that further studies to more fully assess their therapeutic potential may be warranted.

## Methods

### Peptide Synthesis

All reagents employed were of American Chemical Society (ACS) grade or higher and were used without further purification unless otherwise stated. Fmoc-l-Ser-OAllyl and Fmoc-d-Ser-OAllyl,^17^ as well as Alloc-Gly-OH, Alloc-l-Ala-OH, and Alloc-d-Ala-OH,^18^ were synthesized according to referenced literature procedures. Fmoc-l-Orn(Boc)-OH, Fmoc-d-Orn(Boc)-OH, and 4-methylhexanoic acid were purchased from Combi-Blocks. All other Fmoc-amino acids were purchased from P3 BioSystems. 2-Chloro trityl chloride (2-CT) resin was purchased from Iris Biotech. [(1*H*-Benzo[*d*][1,2,3]triazol-1-yl)oxy]tris(dimethylamino)phosphonium hexafluorophosphate (BOP), *N*,*N*-diisopropylcarbodiimide (DIC), and triisopropylsilane (TIPS) were purchased from Manchester Organics. From Sigma-Aldrich were purchased 1 M PMe_3_ in THF and 4-dimethylaminopyridine (DMAP). Phenylsilane was purchased from Thermo Scientific (PhSiH_3_). Fmoc-l-azidoalanine was purchased from Chiralix. Diisopropylethylamine (DIPEA), piperidine, trifluoroacetic acid (TFA), and dimethyl sulfoxide (DMSO) were purchased from Carl Roth. Dichloromethane (CH_2_Cl_2_) and petroleum ether were purchased from VWR Chemicals. Acetonitrile (MeCN), dimethylformamide (DMF), and methyl tertiary-butyl ether (MTBE) were purchased from Biosolve.

2-Chlorotrityl chloride resin (2-CT) (5 g, 1.55 mmol/g) was loaded by overnight coupling via the free side chain hydroxyl of Fmoc-l-Ser-OAllyl (2.84 g, 7.75 mmol, 1 equiv) with DIPEA (1.4 mL, 7.75 mmol, 1 equiv) in 23 mL of CH_2_Cl_2_. The suspension was stirred under argon at 45 °C for 5 min. An additional volume of DIPEA (2.1 mL, 11.1 mmol, 1.5 equiv) was added, and the suspension was stirred under argon at 45 °C overnight. After it was capped with MeOH (0.92 mL, 22.7 mmol, 3 equiv) and DIPEA (0.67 mL, 3.7 mmol, 0.5 equiv) for 15 min, the resin was filtered, washed, and dried overnight under a stream of air. The resin loading was then determined to be 0.37 mmol/g.

Resin loaded with Fmoc-l-Ser-OAllyl (0.68 g, 0.25 mmol) was added to a manual SPPS cartridge and bubbled with nitrogen in DMF (5 mL, 30 min) to swell. Fmoc deprotections (1 min, then 10 min) were carried out with 5 mL of dry piperidine in DMF (1:5, v/v). The next four amino acids (Gly11, Ile10, Thr9, Trp8) were coupled manually (1 h) under nitrogen flow via standard Fmoc solid-phase peptide synthesis (SPPS) (resin/Fmoc-AA/BOP/DIPEA, 1:4:4:8 molar equiv). The following Fmoc amino acids were used: Fmoc-Gly-OH, Fmoc-Ile-OH, Fmoc-Thr-OH (used without side chain protection), and Fmoc-Trp(Boc)-OH. After the coupling of Fmoc-Trp(Boc)-OH, esterification of the Thr side chain was achieved by treating the resin-bound peptide with Alloc-Gly-OH (0.60 g, 3.75 mmol, 15 equiv), DIC (0.59 mL, 3.75 mmol, 15 equiv), and DMAP (15 mg, 0.13 mmol, 0.5 equiv) in 8 mL of CH_2_Cl_2_/DMF (3:1, v/v) for 18 h under argon. The resin was treated with Pd(PPh_3_)_4_ (75 mg, 0.075 mmol, 0.3 equiv) and PhSiH_3_ (0.75 mL, 7.5 mmol, 30 equiv) in CH_2_Cl_2_ (17 mL) under argon for 2 h. The resin was subsequently washed with dry CH_2_Cl_2_ (5 × 5 mL for 3 min), diethyldithiocarbamic acid trihydrate sodium salt in dry DMF (5 mg/mL, 5 × 5 mL for 3 min), and dry DMF (5 × 5 mL for 3 min). Subsequently, BOP (442 mg, 1.0 mmol, 4 equiv) and dry DIPEA (0.35 mL, 2.0 mmol, 8 equiv) were added to cyclize the peptide in 5 mL of DMF, and the suspension was bubbled with nitrogen for 1 h. The remaining N-terminal section of the peptide was then synthesized using the standard SPPS protocol mentioned above. The following Fmoc amino acids were used: Fmoc-d-Orn(Boc)-OH, Fmoc-Gly-OH, Fmoc-l-Orn(Boc)-OH, Fmoc-d-Trp(Boc)-OH, Fmoc-d-Tyr(tBu)-OH, and Fmoc-d-Ser(tBu)-OH. Following the coupling of the last amino acid, the resin was split into two batches of 0.125 mmol. 4-Methylhexanoic acid (34 mg, 0.25 mmol, 2 equiv) was coupled using BOP (221 mg, 0.5 mmol, 4 equiv) and DIPEA (0.17 mL, 1.0 mmol, 8 equiv) in dry DMF (3 mL) under nitrogen flow for 2 h. Final deprotection was carried out by treating the resins with TFA/H_2_O/TIPS (95:2.5:2.5, v/v, 5 mL) for 90 min while shaking. The reaction mixture was filtered through cotton, the filtrate was precipitated from MTBE/petroleum ether (1:1, v/v, 45 mL), and centrifuged (4500 rpm, 5 min). The pellet was then resuspended in MTBE/petroleum ether (1:1, v/v, 50 mL) and centrifuged again (4500 rpm, 5 min). Finally, the pellet containing the crude lipopeptide was dissolved in tBuOH/H_2_O (1:1, v/v, 20 mL) and lyophilized overnight. The crude mixtures were subsequently purified by RP-HPLC (see Purification and Analysis Methods, S8). Fractions were assessed by HPLC and LC-MS, and product-containing fractions were pooled, frozen, and lyophilized to yield pure (95%, determined by HPLC and NMR) lipopeptide **3a** as a white powder in 5% yield over 28 steps.

### Characterization of Relacidines from the Producing Strain

*B. laterosporus* MG64 was cultured on Luria–Bertani (LB) agar and colonies were then grown overnight in 5 mL of LB broth at 37 °C. This inoculum was transferred to 2 L Erlenmeyer flasks containing 500 mL of LB broth and incubated at 37 °C with 220 rpm shaking for 24 h. Cells were collected by centrifugation (10 000 × *g*, 10 min, 4 °C) and extracted with 100 mL of 70% isopropyl alcohol, pH 2 (acidified with 1 M HCl). The supernatant was separated by centrifugation (6000 × *g*, 10 min, 4 °C), and the solvent was evaporated under vacuum. The crude extract was reconstituted in water and filtered with a 0.22 μm syringe filter.

LC-MS analysis was performed using a Shimadzu Nexera X2 UHPLC system coupled to a Shimadzu 9030 QTOF mass spectrometer, as previously described.^19^ Briefly, extracts and pure compounds were dissolved in water to a final concentration of 1 mg/mL and 0.01 mg/mL, respectively, and 2 μL were injected into a Waters Acquity HSS C18 column (1.8 μm, 100 Å, 2.1 × 100 mm). The column was maintained at 30 °C and run at a flow rate of 0.5 mL/min using 0.1% formic acid in water as solvent A and 0.1% formic acid in acetonitrile as solvent B. A gradient was employed for chromatographic separation starting at 15% B for 2 min, then 15−55% B for 9 min, 55−100% B for 0.1 min, and finally held at 100% B for 4 min. The column was re-equilibrated to 15% B for 3 min before the next run was started. The parameters used for the ESI source were: interface voltage 4 kV, interface temperature 300 °C, nebulizing gas flow 3 L/min, and drying gas flow 10 L/min.

### MIC Experiments

Colistin sulfate was purchased from Activate Scientific. Kanamycin monosulfate was purchased from MP Biomedicals. *E. coli* ATCC 25922, *S. aureus* USA300 (ATCC BAA1717), and *K. pneumoniae* ATCC 13883 belong to the American Type Culture Collection (ATCC). *P. aeruginosa* PAO1 was kindly provided by L.H.C. Quarles Van Ufford of Utrecht University, Utrecht, The Netherlands. *E. coli* ATCC 25922 MCR-1 was transfected in-house using the pGDP2-MCR1 plasmid kindly provided by Yong-Xin Li of The University of Hong Kong, Hong Kong, China. Sheep blood agar plates (ref. PB5039A) were purchased from Thermo Scientific. Tryptic soy broth (ref. 02-200-500) was purchased from Scharlab. Mueller-Hinton broth (ref. X927.1) was purchased from Carl Roth. Polypropylene 96-wells plates (ref. 3879) were purchased from Corning.

MICs were determined according to Clinical and Standards Laboratory Institute (CLSI) guidelines. Blood agar plates were inoculated with glycerol stocks of *E*. *coli* ATCC 25922, *K*. *pneumoniae* ATCC 13883, *P. aeruginosa* PAO1, and *S. aureus* USA300. The inoculated agar plates were then incubated for 16 h at 37 °C. Individually grown colonies were subsequently used to inoculate 5 mL aliquots of tryptic soy broth (TSB) that were then incubated at 37 °C with shaking at 220 rpm. *E. coli* 25922 MCR-1 glycerol stock was used to inoculate 5 mL of TSB supplemented with kanamycin that was then incubated for 16 h at 37 °C with shaking at 220 rpm. The next day, the culture was diluted 100-fold in TSB supplemented with kanamycin and incubated at 37 °C with shaking at 220 rpm. In parallel, the lipopeptide antibiotic DMSO stocks to be assessed were serially diluted with Mueller Hinton broth (MHB) in polypropylene 96-well plates (50 μL in each well). Colistin sulfate stocks were dissolved in water before being diluted with MHB. Aliquots of the inoculated TSB were incubated until an OD_600_ of around 0.5 was reached. The bacterial suspensions were then diluted with MHB (2 × 10^5^ cfu mL^–1^) and added to the microplates containing the test compounds (50 μL to each well). The well plates were sealed with an adhesive membrane, and after 18 h of incubation at 37 °C with shaking at 600 rpm, the wells were visually inspected for bacterial growth. MIC values reported are based on three technical replicates and defined as the lowest concentration of the compound that prevented visible growth of bacteria.

### *In vivo* Assays

Ten *G. mellonella* larvae (Serum Therapeutics Inc., average weight of 0.265 g) per group were infected using a 10 μL injection in the right second proleg with mid-log phase (OD_600_ = 0.5)-growing bacteria resuspended and diluted in phosphate-buffered saline (PBS) to achieve the target inoculum of 10^5^ colony forming unit (cfu) per larva. Inoculum density was verified by plating suitable dilutions on nonselective Luria–Bertani agar. Treatment was performed at 1 h postinfection by injecting 10 μL of the indicated compound dose into the left second proleg. The infected larvae were collected in a Petri dish and incubated at 37 °C. The viability of the larvae was assessed twice a day up to a total of 72 h postinfection by checking for movement. Larvae were considered dead if no movement could be observed in response to stimulus with a pipet tip.

## Supporting Information Available

Details of bioinformatic analyses, HRMS data, HPLC traces, and 1D and 2D NMR spectra and for all peptides synthesized and protocols used for hemolysis and serum stability assays (PDF)
