# Supplementary material for: Total Synthesis and Structure Assignment of the Relacidine Lipopeptide Antibiotics and Preparation of Analogues with Enhanced Stability
Source: ACS Infect Dis. 2023 Mar 31;9(4):739–48. doi: 10.1021/acsinfecdis.3c00043 (PMC10111413; doi:10.1021/acsinfecdis.3c00043)
Supplement: Supplementary file 1 — id3c00043_si_001.pdf [file id3c00043_si_001.pdf]

Supplementary Information for:

Total Synthesis and Structure Assignment of the Relacidine Lipopeptide Antibiotics and Preparation of Analogues with Enhanced Stability

Karol Al Ayed[a], Denise Zamarbide Losada[a], Nataliia V. Machushynets[b], Barbara Terlouw[c], Somayah S. Elsayed[b], Julian Schill[d], Vincent Trebosc[d], Michel Pieren[d], Marnix H. Medema[c], Gilles P. van Wezel[b] and Nathaniel I. Martin[a,*]

[a]Biological Chemistry Group, Institute of Biology, Leiden University, Sylviusweg 72, 2333 BE Leiden, NL
[*]E-mail: n.i.martin@biology.leidenuniv.nl

[b]Molecular Biotechnology Group, Institute of Biology, Leiden University, Sylviusweg 72, 2333 BE Leiden, NL

[c]Bioinformatics Group, Wageningen University, Droevendaalsesteeg 1, 6708 PB Wageningen, NL

[d]BioVersys AG, c/o Technologiepark, Hochbergerstrasse 60c, CH-4057 Basel, CH

TABLE OF CONTENTS

Section	Contents	Page Number
I	Bioinformatics	S2
II	^{1}H-NMR comparison of relacidines	S3
III	Materials	S4
IV	General procedures for peptide synthesis	S5
V	Purification and analysis methods	S8
VI	Culturing conditions and extraction natural products	S9
VII	LC-MS/MS	S10
VIII	Antimicrobial testing	S11
IX	Hemolytic assay	S12
X	Serum stability assay	S13
XI	*In vivo* experiments	S14
XII	HPLC and HRMS analysis of peptides	S15
XIII	NMR characterization	S19
XIV	References	S40

I. Bioinformatics

We extracted the A-domains from the relacidine, laterocidine and brevicidine BGCs with HMMer (3.3.2) (http://hmmer.org), using the AMP-binding domain profile hidden Markov model (HMM) also used by antiSMASH:
https://github.com/antismash/antismash/blob/master/antismash/detection/hmm_detection/data/AMP-binding.hmm

We extracted the active sites of these A-domains and predicted their substrates using PARAS (v0.0.1; unpublished; code available at https://pypi.org/project/paras/). We predicted the structures of the Gly-recognizing A-domains of the relacidine, laterocidine and brevicidine BGCs (Table S1) with AlphaFold2, building separate structure models for the N-terminal A-subdomain and the C-terminal A-subdomain. In pymol, we combined the predicted subdomains into complete structures by aligning them to the PDB structure 1AMU, a published X-ray crystallography structure of an A-domain bound to its substrate phenylalanine. We positioned the alanine in the active site of the predicted A-domain structures by mutating the phenylalanine substrate in 1AMU to alanine and measured the distance between the alanine residue and W291/Y290 (or equivalent) with pymol's measurement wizard (Table S1).

Table S1: Distances between the modelled alanine substrate and residue 290 based on AlphaFold structure models. The domains visualized in Fig. 3B are indicated in bold.

Domain	Module nr	Residue at position 290 or equivalent	Distance between Ala substrate and residue 290
RlcC A6	6	W290	1.9Å
RlcD A2	11	W294	2.4Å
**RlcD A4**	**13**	**Y290**	**3.4Å**
LatC A6	6	W290	2.5Å
LatD A3	12	W297	2.2Å
**LatD A4**	**13**	**W291**	**2.4Å**
BreC A6	6	W290	2.3Å
BreD A2	11	W297	2.4Å

II. ^{1}H-NMR comparison of relacidines

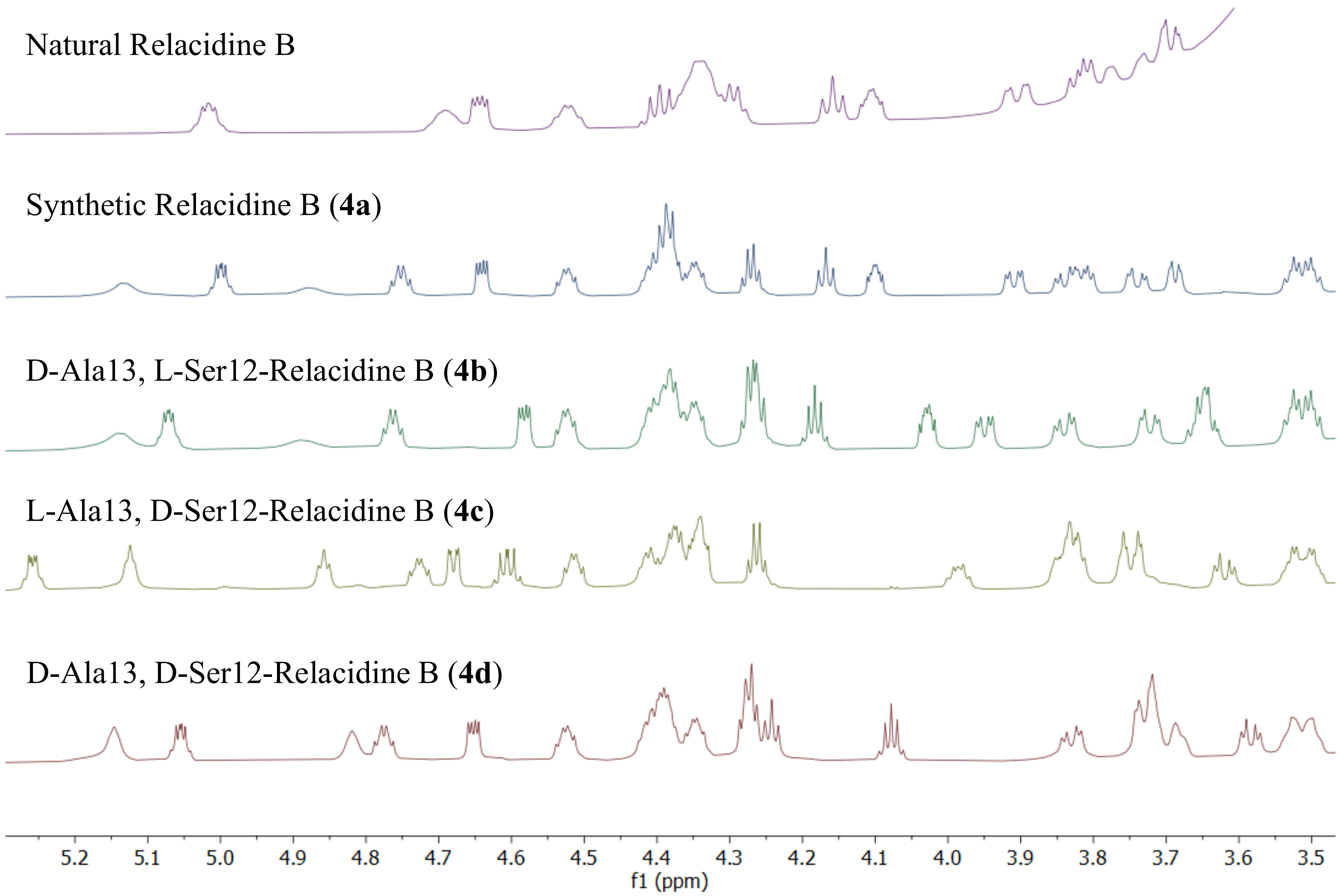

Figure S1. Previously published[1] ^{1}H-NMR (600 MHz, d_6-DMSO) spectrum of natural relacidine B isolated after fermentation of the producing organism overlaid with ^{1}H-NMR (850 MHz, d_6-DMSO) spectra of synthetic relacidines **4a-4d**. Spectra were recorded at room temperature.

III. Materials

Synthesis

All reagents employed were of American Chemical Society (ACS) grade or higher and were used without further purification unless otherwise stated. Fmoc-L-Ser-OAllyl and Fmoc-D-Ser-OAllyl[2] as well as Alloc-Gly-OH, Alloc-L-Ala-OH and Alloc-D-Ala-OH[3] were synthesized according to referenced literature procedures. Fmoc-L-Orn(Boc)-OH, Fmoc-D-Orn(Boc)-OH and 4-methylhexanoic acid were purchased from Combi-Blocks. All other Fmoc-amino acids were purchased from P3 BioSystems. 2-Chloro trityl chloride (2-CT) resin was purchased from Iris Biotech. ((1H-Benzo[d][1,2,3]triazol-1-yl)oxy)tris(dimethylamino)phosphonium hexafluorophosphate (BOP), N,N-Diisopropylcarbodiimide (DIC) and triisopropylsilane (TIPS) were purchased from Manchester Organics. 1M PMe_3 in THF and 4-Dimethylaminopyridine (DMAP) were purchased from Sigma Aldrich. Phenylsilane was purchased from Thermo Scientific ($PhSiH_3$). Fmoc-L-azidoalanine was purchased from Chiralix. Diisopropylethylamine (DIPEA), piperidine, trifluoroacetic acid (TFA) and dimethyl sulfoxide (DMSO) were purchased from Carl Roth. Dichloromethane (CH_2Cl_2) and petroleum ether were purchased from VWR Chemicals. Acetonitrile (MeCN), dimethylformamide (DMF) and methyl tertiary-butyl ether (MTBE) were purchased from Biosolve.

IV. General procedures for peptide synthesis

Resin Loading

2-Chlorotrityl chloride resin (2-CT) (5 g, 1.55 mmol/g) was loaded by overnight coupling via the free sidechain hydroxyl of Fmoc-L-Ser-OAllyl (2.84 g, 7.75 mmol, 1 eq.) or Fmoc-D-Ser-OAllyl (1.5 g, 7.75 mmol, 1 eq.) with DIPEA (1.4 mL, 7.75 mmol, 1 eq.) in 23 mL of CH_2Cl_2. The suspension was stirred under argon at 45ºC for 5 min. An additional volume of DIPEA (2.1 mL, 11.1 mmol, 1.5 eq.) was added, the suspension was stirred under argon at 45ºC, overnight. After capping with MeOH (0.92 mL, 22.7 mmol, 3 eq.) and DIPEA (0.67 mL, 3.7 mmol, 0.5 eq.) for 15 min, the resin was filtered, washed and dried overnight under a stream of air. The resin loading was then determined to be 0.37 mmol/g and 0.33 mmol/g for 2-CT-Fmoc-L-Ser-OAllyl and 2-CT-Fmoc-D-Ser-OAllyl respectively.

Synthesis of Relacidines (3a-4d)

Resin loaded with Fmoc-L-Ser-OAllyl (0.68 g, 0.25 mmol) or Fmoc-D-Ser-OAllyl (0.75 g, 0.25 mmol) was added to a manual SPPS cartridge and bubbled with nitrogen in DMF (5 mL, 30 min) to swell. Fmoc deprotections (1 min then 10 min) were carried out with 5 mL of dry piperidine in DMF (1 : 5, v/v). The next 4 amino acids (Gly11, Ile10, Thr9, Trp8) were coupled manually (1 h) under nitrogen flow via standard Fmoc solid-phase peptide synthesis (SPPS) (resin : Fmoc-AA : BOP : DIPEA, 1 : 4 : 4 : 8 molar eq.). The following Fmoc amino acids were used: Fmoc-Gly-OH, Fmoc-Ile-OH, Fmoc-Thr-OH (used without sidechain protection) and Fmoc-Trp(Boc)-OH. After coupling of Fmoc-Trp(Boc)-OH, esterification of the Thr sidechain was achieved by treating the resin-bound peptide with Alloc-Gly-OH (0.60 g, 3.75 mmol, 15 eq.), DIC (0.59 mL, 3.75 mmol, 15 eq.), and DMAP (15 mg, 0.13 mmol, 0.5 eq.) in 8 mL CH_2Cl_2 : DMF (3 : 1, v/v) for 18 h under argon, for relacidine A; or Alloc-L-Ala-OH or Alloc-D-Ala-OH (0.65 g, 3.75 mmol, 15 eq.), DIC (0.59 mL, 3.75 mmol, 15 eq.), and DMAP (15 mg, 0.13 mmol, 0.5 eq.) in 8 mL CH_2Cl_2 : DMF (3 : 1, v/v) for 18 h under argon, for relacidine B. The resin was treated with $Pd(PPh_3)_4$ (75 mg, 0.075 mmol, 0.3 eq.), and $PhSiH_3$ (0.75 mL, 7.5 mmol, 30 eq.) in CH_2Cl_2 (16.5 mL) under argon for 2 h. The resin was subsequently washed with dry CH_2Cl_2 (5 x 5 mL x 3 min), diethyldithiocarbamic acid trihydrate sodium salt in dry DMF (5 mg/mL, 5 x 5 mL x 3 min), and dry DMF (5 x 5 mL x 3 min). Subsequently, BOP (442 mg, 1.0 mmol, 4 eq.) and dry DIPEA (0.35 mL, 2.0 mmol, 8 eq.) were added to cyclize the peptide in 5 mL of DMF, the suspension was bubbled with nitrogen for 1 h. The remaining N-terminal section of the peptide was then synthesized using the standard SPPS protocol mentioned above. The following Fmoc amino acids were used: Fmoc-D-Orn(Boc)-OH, Fmoc-Gly-OH, Fmoc-L-Orn(Boc)-OH, Fmoc-D-Trp(Boc)-OH,

Fmoc-D-Tyr(tBu)-OH, and Fmoc-D-Ser(tBu)-OH. Following the coupling of the last amino acid, the resin was split into two batches of 0.125 mmol. 4-Methylhexanoic acid (34 mg, 0.25 mmol, 2 eq.) was coupled using BOP (221 mg, 0.5 mmol, 4 eq.), and DIPEA (0.17 mL, 1.0 mmol, 8 eq.) in dry DMF (3 mL), under nitrogen flow for 2 h. Final deprotection was carried out by treating the resins with TFA : H_2O : TIPS (95 : 2.5 : 2.5, v/v, 5 mL) for 90 min while shaking. The reaction mixture was filtered through cotton, the filtrate was precipitated from MTBE : petroleum ether (1 : 1, v/v, 45 mL) and centrifuged (4500 rpm, 5 min). The pellet was then resuspended in MTBE : petroleum ether (1 : 1, v/v, 50 mL) and centrifuged again (4500 rpm, 5 min). Finally the pellet containing the crude lipopeptide was dissolved in tBuOH : H_2O (1 : 1, v/v, 20 mL) and lyophilized overnight. The crude mixtures were subsequently purified by RP-HPLC (See Purification and analysis methods). Fractions were assessed by HPLC and LC-MS and product containing fractions were pooled, frozen and lyophilized to yield the pure lipopeptides as white powders in 3–10% yield over 28 steps. See section V, HPLC and HRMS analysis of peptides, for traces and individual yields.

Synthesis of Relacidamide (5)

2-Chlorotrityl resin (2-CT) (5.0 g, 1.60 mmol/g) was loaded with 1 eq. Fmoc-Gly-OH following the same protocol as described above. Resin loading was determined to be 0.67 mmol/g. The linear peptide was assembled manually on a 0.25 mmol scale under nitrogen flow via standard Fmoc solid-phase peptide synthesis (SPPS) (1 h couplings, resin : Fmoc-AA : BOP : DIPEA, 1 : 4 : 4 : 8 molar eq.). DMF (5 mL) was used as solvent and Fmoc deprotections (5 min then 15 min) were carried out with 5 mL piperidine : DMF (1 : 4, v/v). The following Fmoc amino acids were used: Fmoc-D-Ser(tBu)-OH, Fmoc-D-Tyr(tBu)-OH, Fmoc-D-Trp(Boc)-OH, Fmoc-D-Orn(Boc)-OH, Fmoc-L-Orn(Boc)-OH, Fmoc-Gly-OH, Fmoc-Trp(Boc)-OH, Fmoc-L-azidoalanine, Fmoc-Ile-OH and Fmoc-Ser(tBu)-OH. Following the final Fmoc removal step, 4-methylhexanoic acid (65 mg, 0.5 mmol, 2 eq.) was coupled using BOP (221 mg, 0.5 mmol, 2 eq.) and DIPEA (0.17 mL, 1.0 mmol, 4 eq.) in 5 mL of DMF overnight, under nitrogen flow. The azide was reduced by treating the resin with 9 mL of 1M PMe_3 in THF and 1 mL of H_2O for 3 hours. After washing the resin with DMF and CH_2Cl_2, the peptide was cleaved off the resin by treating it with HFIP : CH_2Cl_2 (1 : 4, v/v, 20 mL) for 1 hour and rinsed with additional HFIP : CH_2Cl_2 and CH_2Cl_2. The combined washings were then evaporated to yield the linear protected peptide with a free C-terminus and amino sidechain. The peptide was dissolved in 250 mL of CH_2Cl_2 and 50 mL of DMF, treated with BOP (221 mg, 0.5 mmol, 2 eq.) and DIPEA (0.17 mL, 1.0 mmol, 4 eq.) and the solution was stirred overnight under nitrogen atmosphere. The reaction mixture was concentrated *in vacuo* and directly treated with TFA : H_2O : TIPS (95 : 2.5 : 2.5, v/v, 10 mL) for 90 min while shaking.

The reaction mixture was filtered through cotton, the filtrate was precipitated from MTBE : petroleum ether (1 : 1, v/v, 45 mL) and centrifuged (4500 rpm, 5 min). The pellet was then resuspended in MTBE : petroleum ether (1 : 1, v/v, 50 mL) and centrifuged again (4500 rpm, 5 min). Finally the pellet containing the crude lipopeptide was dissolved in tBuOH : H_2O (1 : 1, v/v, 20 mL) and lyophilized overnight. The crude mixture were subsequently purified by RP-HPLC (See Purification and analysis methods). Fractions were assessed by HPLC and LC-MS and product containing fractions were pooled, frozen and lyophilized to yield the pure lipopeptide as white powder in 18% yield over 30 steps.

V. Purification and analysis methods

Preparative HPLC: Purification was performed on a BESTA-Technik system with a Dr. Maisch ReproSil Gold 120 C18 column (10 µm, 25 x 250 mm) and equipped with a ECOM Flash UV detector. Runs were performed at a flow rate of 12 mL/min with UV detection at 214 nm and 254 nm. Solvent A = 0.1% TFA in water/MeCN (95 : 5) and solvent B = 0.1% TFA in water/MeCN (5 : 95). A gradient method was employed, starting at 100 % solvent A for 2 min, ramping up to 100 % solvent B over 55 min, remaining at 100 % solvent B for 3 min before ramping down to 100 % solvent A over 1 min and remaining there for 1 min. Product containing fractions were pooled, partially concentrated under vacuum, frozen and then lyophilized to yield pure peptides as white flocculent solids. A small amount of purified peptide was analyzed by analytical HPLC.

Analytical HPLC: Analytical runs were performed on a Shimadzu Prominence-i LC-2030 system with a Dr. Maisch ReproSil Gold 120 C18 (5 µm, 4.6 x 250 mm) at 30 °C. Runs were performed at a flow rate of 1 mL/min with UV detection at 214 nm and 254 nm. Solvent A = 0.1% TFA in water/MeCN (95 : 5) and solvent B = 0.1% TFA in water/MeCN (5 : 95). A gradient method was employed, starting at 100% solvent A for 2 min, ramping up to 50 % solvent B over 23 min, ramping up to 100% solvent B over 1 min, remaining there for 2 min before ramping down to 100% solvent A over 1 min and remaining there for 1 min.

HRMS: HRMS spectra were acquired on a Thermo Scientific Dionex UltiMate 3000 HPLC system with a Phenomenex Kinetex C18 (2.6 µm, 2.1 x 150 mm) column at 35 °C and equipped with a diode array detector. The following solvent system, at a flow rate of 0.3 mL/min, was used: solvent A = 0.1% formic acid in water, solvent B = 0.1% formic acid in MeCN. A gradient method was employed, starting at 95 % solvent A and 5 % solvent B for 1 min, ramping up to 95 % solvent B over 9 min, ramping up to 98 % solvent B over 1 min, remaining there for 1 min before ramping back down to 95 % solvent A over 2 min and remaining there for 1 min. The system was connected to a Bruker micrOTOF-Q II mass spectrometer (ESI ionization) calibrated internally with sodium formate.

VI. Culturing conditions and extraction of natural products

Brevibacillus laterosporus MG64 was cultured on Luria-Bertani (LB) agar and colonies grown overnight in 5 ml LB broth at 37°C. This inoculum was transferred to 2 L Erlenmeyer flasks containing 500 ml of LB broth and incubated at 37°C with 220 rpm shaking for 24 h. Cells were collected by centrifugation (10,000 × *g*, 10 min, 4°C) and extracted with 100 mL of 70% isopropyl alcohol (IPA), pH 2 (acidified with 1 M HCl). Supernatant was separated by centrifugation (6000×g, 10 min, 4 °C) and solvent was evaporated under vacuum using rotary evaporation. The crude extract was reconstituted in H_2O and filtered with a 0.22 µm syringe filter.

VII. LC–MS/MS analysis

LC–MS analysis was performed using a Shimadzu Nexera X2 UHPLC system coupled to a Shimadzu 9030 QTOF mass spectrometer as previously described.[4] Briefly, extracts and pure compounds were dissolved in H_2O to a final concentration of 1 mg/mL and 0.01 mg/mL, and 2 μL was injected into a Waters Acquity HSS C18 column (1.8 μm, 100 Å, 2.1 × 100 mm). The column was maintained at 30 °C, and run at a flow rate of 0.5 mL/min, using 0.1% formic acid in water as solvent A, and 0.1% formic acid in MeCN as solvent B. A gradient was employed for chromatographic separation starting at 5% B for 1 min, then 5 – 85% B for 9 min, 85 – 100% B for 1 min, and finally held at 100% B for 4 min. The column was re-equilibrated to 5% B for 3 min before the next run was started. The parameters used for the ESI source were: interface voltage 4 kV, interface temperature 300 °C, nebulizing gas flow 3 L/min, and drying gas flow 10 L/min.

VIII. Antimicrobial testing

Table S2. Minimum inhibitory concentrations (MICs) determined against panel of previously characterized colistin-resistant *A. baumannii* clinical isolates[5]

	Strain	Relacidine A (3a)	Relacidine B (4a)	Relacidamide (5)	Colistin
Peptide MIC[a]	*A. baumannii* ATCC 17978	8	4	4	1
	A. baumannii NCTC 13304	4	4	4	1
	A. baumannii NCTC 13420	8	16	16	0.5
	A. baumannii HUMC1	16	8	8	1
	A. baumannii LAC-4	4	8	8	0.5
	A. baumannii BV94	8	16	8	>64
	A. baumannii BV95	8	8	8	32
	A. baumannii BV172	16	16	16	>64
	A. baumannii BV173	8	16	16	16

[a]MIC values given in µg/ml.

All minimum inhibitory concentrations were determined according to Clinical and Standards Laboratory Institute (CLSI) guidelines. Blood agar plates were inoculated from glycerol stocks of the different *A. baumannii* strains used and then incubated for 16 h at 37 °C. Individually grown colonies were subsequently used to inoculate 5 mL aliquots of TSB that were then incubated at 37 °C. In parallel, the lipopeptide antibiotics DMSO stocks to be assessed were serially diluted with cation-adjusted MHB in polypropylene 96-well plates (50 µL in each well). Colistin sulfate stocks were dissolved in water before being diluted with cation-adjusted MHB. The *A. baumannii* inoculated TSB aliquots were incubated until an OD_{600} of around 0.5 was reached. The bacterial suspensions were then diluted with cation-adjusted MHB (2 x 10^5 CFU mL^{-1}) and added to the microplates containing the test compounds (50 µL to each well). The well-plates were sealed with an adhesive membrane and after 18 h of incubation at 37 °C visually inspected for bacterial growth. MIC values reported are based on three technical replicates and defined as the lowest concentration of the compound that prevented visible growth of bacteria.

IX. Hemolytic assay

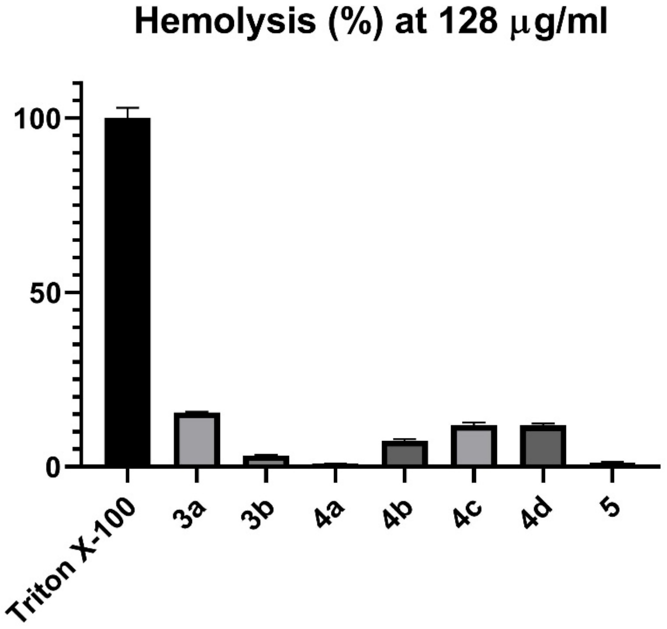

Figure S2. Hemolytic activity of compounds **3a-5** at 128 μg/mL and 1 h incubation against sheep red blood cells. Colistin was also included as a reference and showed no detectable hemolysis (<0.1%) under the same conditions.

Experiments were performed in triplicate and Triton X-100 used as a positive control. Red blood cells from defibrinated sheep blood obtained from Thermo Fisher were centrifuged (400 g for 15 min at 4°C) and washed with Phosphate-Buffered Saline (PBS) containing 0.002% Tween20 (buffer) five times. Then, the red blood cells were normalized to obtain a positive control read-out between 2.5 and 3.0 at 415 nm to stay within the linear range with the maximum sensitivity. A serial dilution of the compounds (128 – 1 μg/mL, 75 μL) was prepared in a 96-well polypropylene plate. The outer border of the plate was filled with 75 μL buffer. Each plate contained a positive control (0.1% Triton-X final concentration, 75 μL) and a negative control (buffer, 75 μL) in triplicate. The normalized blood cells (75 μL) were added and the plates were incubated at 37 °C for 1 h while shaking at 500 rpm. A flat-bottom polystyrene plate with 100 μL buffer in each well was prepared. After incubation, the plates were centrifuged (800 g for 5 min at room temperature) and 25 μL of the supernatant was transferred to their respective wells in the flat-bottom plate. The values obtained from a read-out at 415 nm were corrected for background (negative control) and transformed to a percentage relative to the positive control.

X. Serum Stability assay

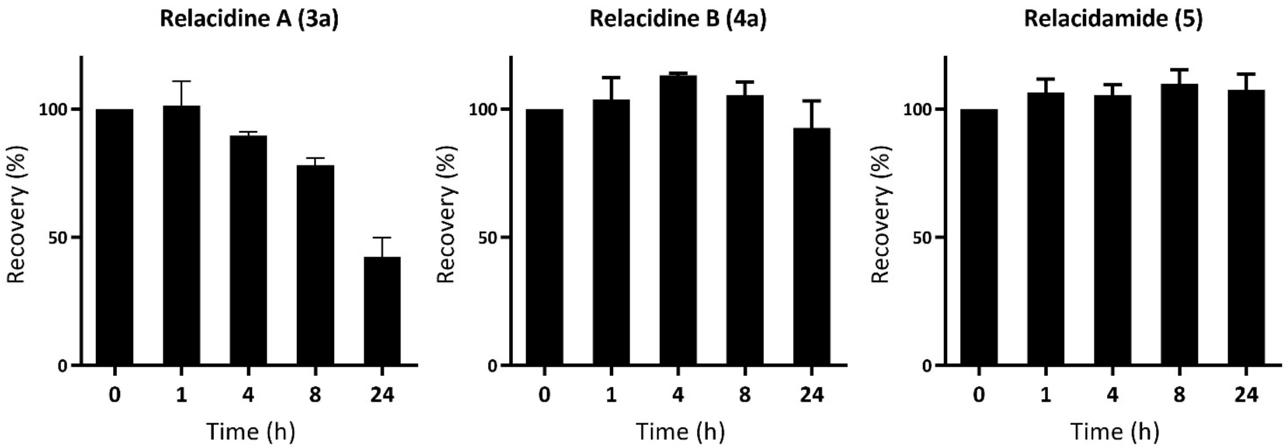

Figure S3. Serum stability assay comparing natural lipopeptides **3a** and **4a** to relacidamide analogue **5**.

10 mg/mL peptide solutions were prepared in in Milli-Q water. Samples were prepared with 42 µL peptide solution and 518 µL human serum (obtained from Sigma Aldrich, product number: H4522) and incubated at 37 °C. Samples were taken at t = 0, 1, 4, 8 and 24 h. To 100 µL of serum, 100 µL of 6% trichloroacetic acid in acetonitrile (containing 0.2 µg/mL D-Phenylalanine as internal standard) was added to precipitate the proteins. The samples were vortexed, left for 15 min at room temperature and stored at -20 °C. Before analysis the samples were centrifuged for 5 min at 13 000 rpm. The supernatant was analyzed by RP-HPLC using a Shimadzu Prominence-i LC-2030 system with a Dr. Maisch ReproSil Gold 120 C18 column (4.6 × 250 mm, 5 µm) at 30 °C and equipped with a UV detector monitoring at 220 nm and 254 nm. The following solvent system, at a flow rate of 1 mL/min, was used: solvent A, 0.1 % TFA in water/acetonitrile 95/5; solvent B, 0.1 % TFA in water/acetonitrile 5/95. Gradient elution was as follows: 100:0 (A/B) for 2 min, 100:0 to 50:50 (A/B) over 45 min, 50:50 (A/B) to 0:100 (A/B) over 1 min, 0:100 (A/B) for 6 min then reversion back to 100:0 (A/B) over 1 min, 100:0 (A/B) for 5 min. The peaks were integrated and normalized to the internal standard. The t=0 value was then set at 100% for each analogue and all time-points were calculated as a percentage of t=0. Biological duplicates of the experiment were performed.

XI. *In vivo* experiments

Ten *G. mellonella* larvae (Serum Therapeutics Inc., average weight 0.265 g) per group were infected using a 10-µl injection in the right second proleg with mid-log phase (OD_{600} = 0.5) growing bacteria resuspended and diluted in phosphate-buffered saline (PBS) to achieve the target inoculum of 10^5 colony forming unit (cfu) per larva. Inoculum density was verified by plating suitable dilutions on non-selective Luria-Bertani agar. Treatment was performed at 1 hour post-infection by injecting 10 µl of the indicated compound dose in the left second proleg. The infected larvae were collected in a Petri dish and incubated at 37°C. The viability of the larvae was assessed twice a day up to a total of 72 hours post-infection by checking for movement. Larvae were considered dead if no movement could be observed in response to stimulus with a pipette tip.

XII. HPLC and HRMS analysis of peptides

Table S3. Peptide number, name, chemical formula, exact mass, mass found and overall yield for peptides **3a**-**5**.

**Peptide**	**Name**	**Chemical Formula**	**Calcd Exact Mass**	**Mass found**	**Calcd**	**Overal l Yield [%]**
**3a**	Relacidine A	$C_{75}H_{108}N_{18}O_{18}$	1548.8089	775.4117	775.4118	5.4
**3b**	D-Ser12-Relacidine A	$C_{75}H_{108}N_{18}O_{18}$	1548.8089	775.4120	775.4118	6.8
**4a**	Relacidine B	$C_{76}H_{110}N_{18}O_{18}$	1562.8245	782.4199	782.4195	9.1
**4b**	D-Ala13, L-Ser12-Relacidine B	$C_{76}H_{110}N_{18}O_{18}$	1562.8245	782.4197	782.4195	9.5
**4c**	L-Ala13, D-Ser12-Relacdine B	$C_{76}H_{110}N_{18}O_{18}$	1562.8245	782.4199	782.4195	3.2
**4d**	D-Ala13, D-Ser12-Relacdine B	$C_{76}H_{110}N_{18}O_{18}$	1562.8245	782.4195	782.4195	5.7
**5**	Relacidamide	$C_{74}H_{107}N_{19}O_{17}$	1533.8092	767.9120	767.9119	18

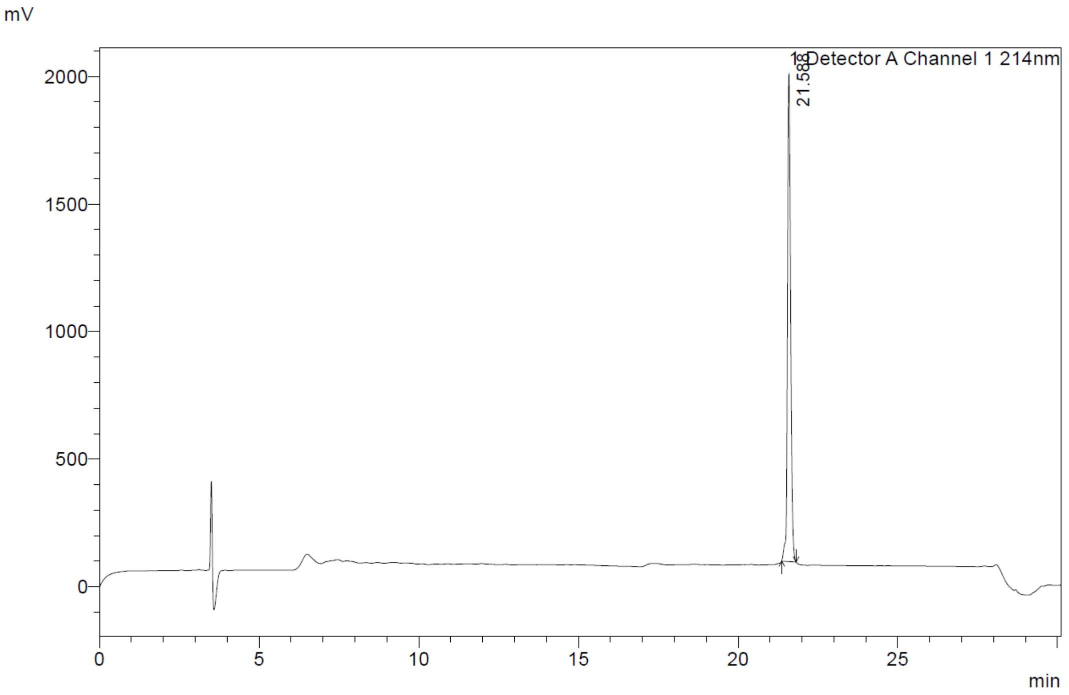

Figure S4. HPLC trace showing the reinjection of purified (**3a**). The peptide eluted as a single peak at 21.588 min using the HPLC method outlined in part.

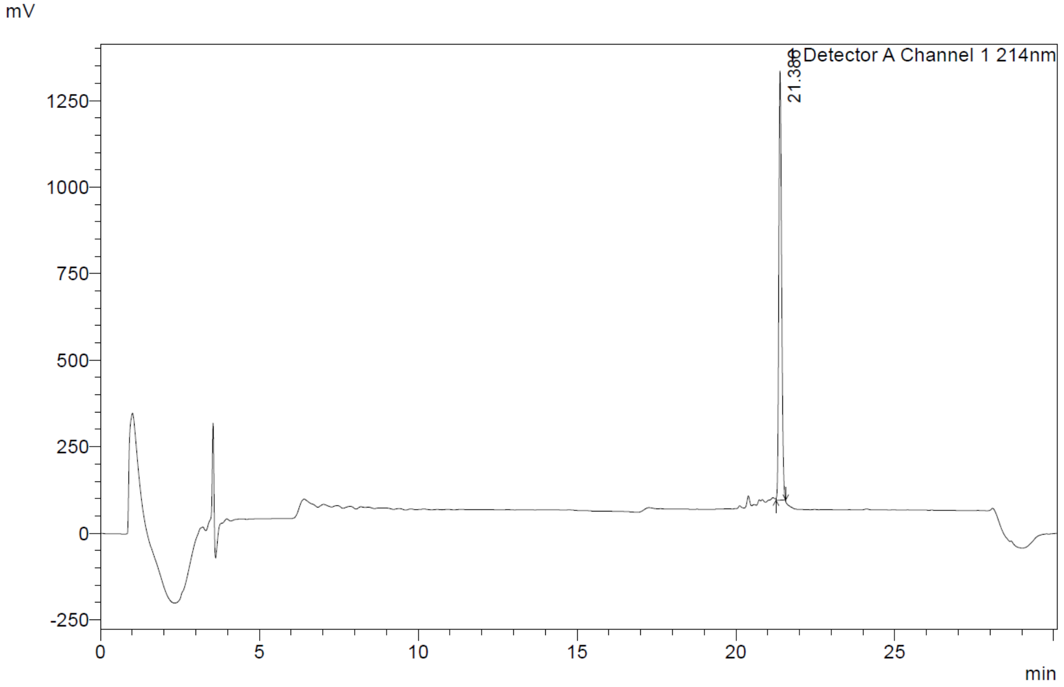

Figure S5. HPLC trace showing the reinjection of purified (**3b**). The peptide eluted as a single peak at 21.386 min using the HPLC method outlined in part.

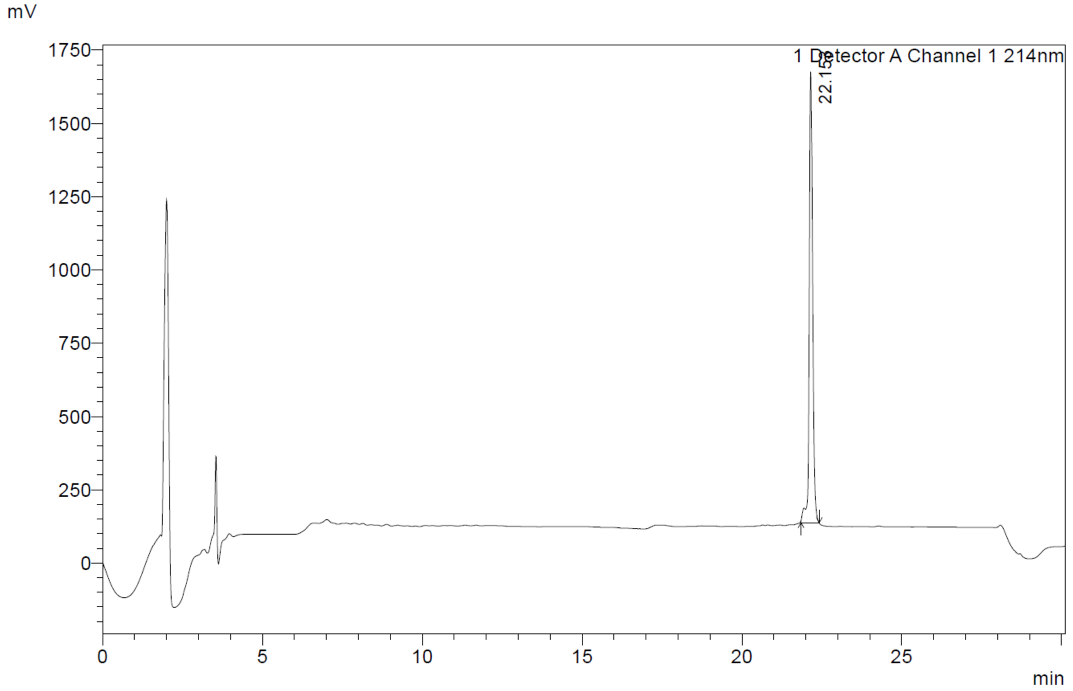

Figure S6. HPLC trace showing the reinjection of purified (**4a**). The peptide eluted as a single peak at 22.153 min using the HPLC method outlined in part.

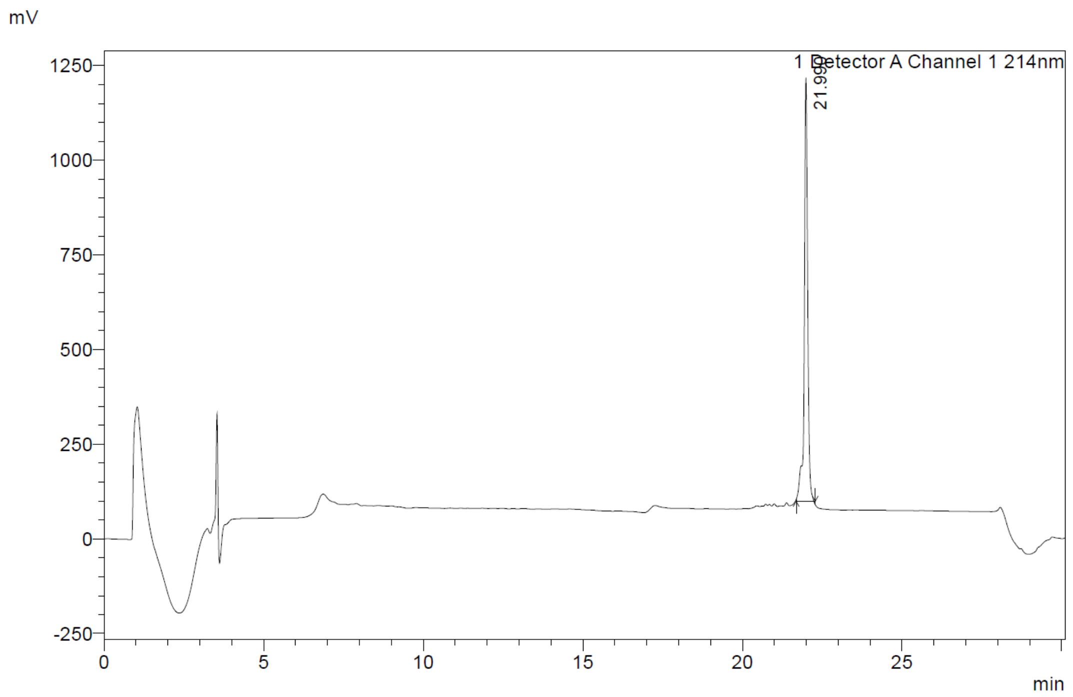

Figure S7. HPLC trace showing the reinjection of purified (**4b**). The peptide eluted as a single peak at 21.990 min using the HPLC method outlined in part.

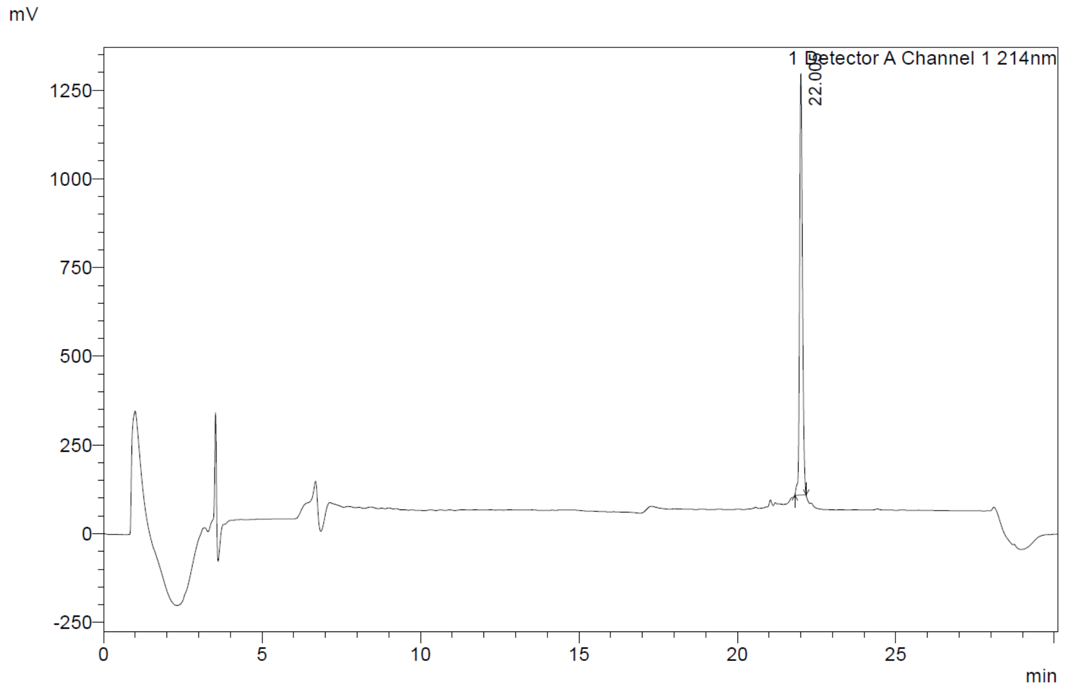

Figure S8. HPLC trace showing the reinjection of purified (**4c**). The peptide eluted as a single peak at 22.005 min using the HPLC method outlined in part.

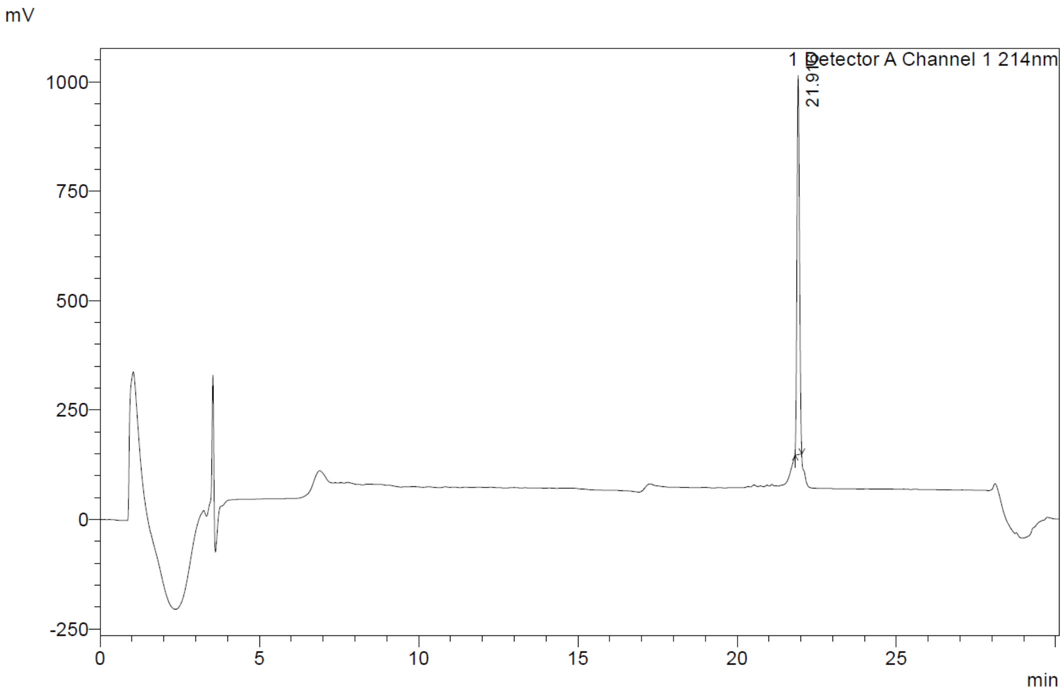

Figure S9. HPLC trace showing the reinjection of purified (**4d**). The peptide eluted as a single peak at 21.916 min using the HPLC method outlined in part.

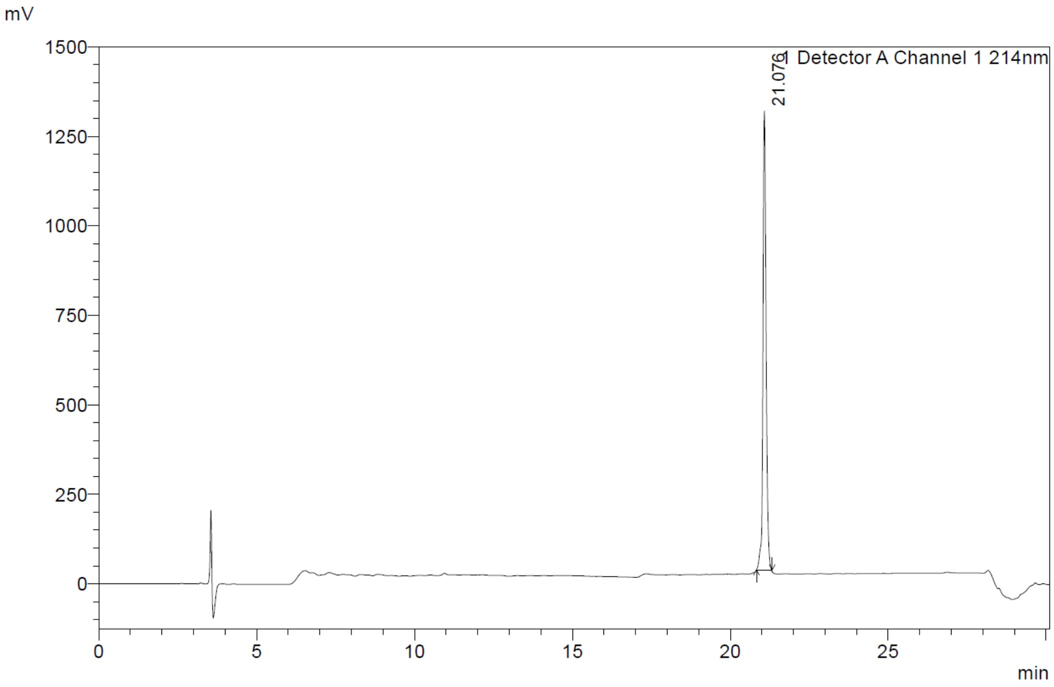

Figure S10. HPLC trace showing the reinjection of purified (**5**). The peptide eluted as a single peak at 21.076 min using the HPLC method outlined in part.

XIII. NMR characterization

Table S4. ^{1}H-NMR (d_6-DMSO) characterization of relacidine A **(3a)**

Residue	-NH	Hα	Hβ	Hγ	Hδ	Hε
D-Ser1	7.88 (1H, d, *J* = 7.4 Hz)	4.27 (1H, m)	3.51 (2H, m)	5.11 (1H, t, *J* = 5.4 Hz)		
D-Tyr2	8.01 (1H, m)	4.36 (1H, m)	2.83 (1H, dd, *J* = 14.2, 4.3 Hz) & 2.62 (1H, m)	Phenol: 9.17 (1H, s), 6.89 (2H, d, *J* = 8.5 Hz), 6.57 (2H, d, *J* = 8.5 Hz)		
D-Trp3	8.00 (1H, m)	4.52 (1H, m)	3.17 (1H, m) & 2.95 (1H, m)	Indole: 10.79 (1H, s), 7.57 (1H, d, *J* = 7.9 Hz), 7.34 (1H, d, *J* = 8.1 Hz), 7.15 (1H, d, *J* = 2.3 Hz), 7.07 (1H, m)), 6.99 (1H, t, *J* = 7.2 Hz)		
D-Orn4	8.04 (1H, d, *J* = 8.1 Hz)	4.38 (1H, m)	1.73 (1H, m) & 1.56 (1H, m)	1.56 (2H, m)	2.78 (2H, m)	7.69 (2H, br m)
Orn5	8.08 (1H, d, *J* = 8.1 Hz)	4.41 (1H, m)	1.71 (1H, m) & 1.53 (1H, m)	1.53 (2H, m)	2.77 (2H, m)	7.69 (2H, br m)
Gly6	8.26 (1H, m)	3.83 (1H, m) & 3.73 (1H, m)				
D-Orn7	8.11 (1H, m)	4.38 (1H, m)	1.40 (1H, m) & 1.28 (1H, m)	1.33 (2H, m)	2.59 (2H, m)	7.62 (2H, br m)
Trp8	8.18 (1H, d, *J* = 8.2 Hz)	4,76 (1H, m)	3.13 (1H, dd, *J* = 14.5, 5.1 Hz) & 2.95 (1H, m)	Indole: 10.77 (1H, s), 7.57 (1H, d, *J* = 7.9 Hz), 7.31 (1H, d, *J* = 8.1 Hz), 7.09 (1H, d, *J* = 2.4 Hz), 7.04 (1H, m), 6.95 (1H, t, *J* = 7.2 Hz)		
Thr9	8.08 (1H, d, *J* = 8.1 Hz)	4.58 (1H, dd, *J* = 8.3, 4.1 Hz)	4.96 (1H, m)	1.02 (3H, d, *J* = 6.6 Hz)		
Ile10	7.95 (1H, d, *J* = 9.2 Hz)	4.27 (1H, m)	1.67 (1H, m)	1.50 (1H, m) & 1.10 (1H, m), 0.84 (3H, d, *J* = 6.8 Hz)	0.86 (3H, t, *J* = 7.4 Hz)	
Gly11	9.19 (1H, t, *J* = 5.1 Hz)	3.94 (1H, m) & 3.33 (1H, under H_2O)				
Ser12	8.56 (1H, d, *J* = 8.1 Hz)	4.12 (1H, m)	3.74 (1H, m) & 3.66 (1H, m)	4.88 (1H, t, *J* = 5.7 Hz)		
Gly13	8.12 (1H, m)	3.94 (1H, m) & 3.81 (1H, m)				
Lipid	2.12 (2H, m, $O{=}CC\mathbf{H_2}$), 1.50 (2H, $O{=}CCH_2C\mathbf{H_2}$), 1.27 (5H, -$\mathbf{CH_2CH}(CH)_3\mathbf{CH_2}CH_3$), 1.09 (2H, -$\mathbf{CH_2}CH_3$), 0.82 (6H, -$CH(C\mathbf{H_3})CH_2C\mathbf{H_3}$)					

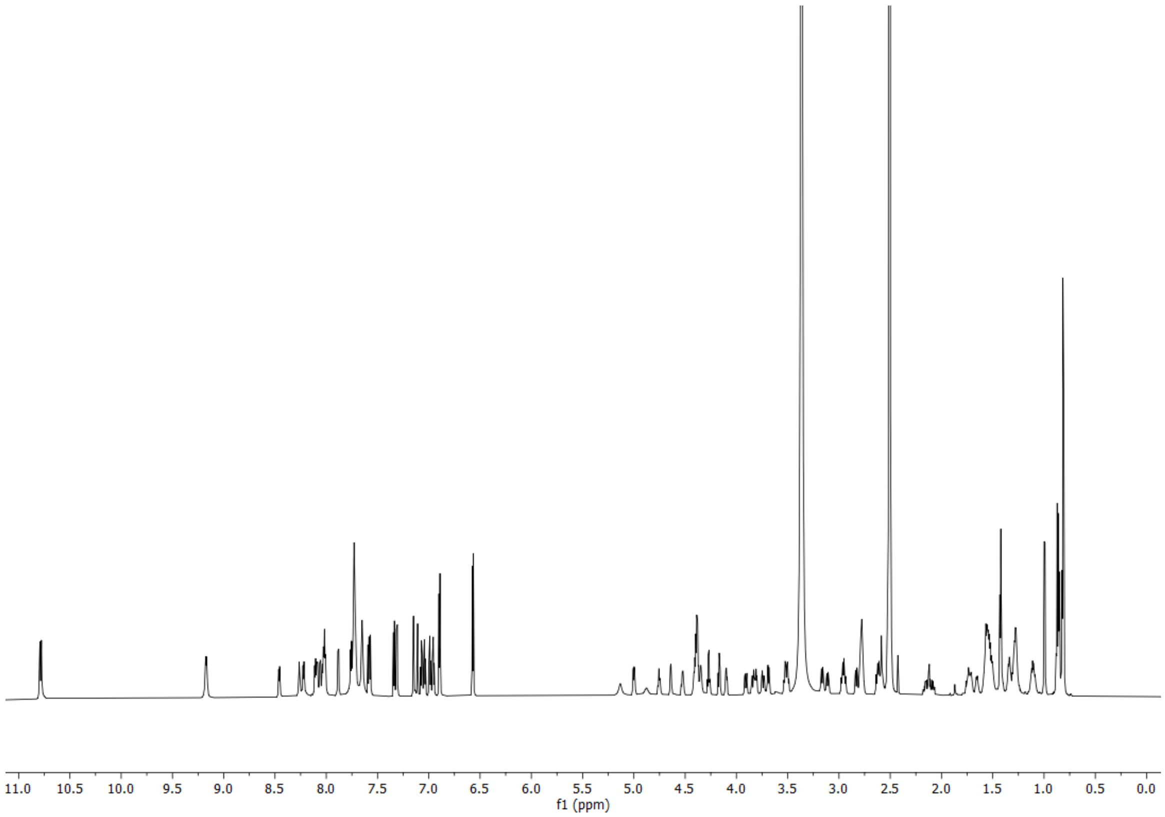

Figure S11. ^{1}H-NMR (d_6-DMSO) of relacidine A **(3a)**

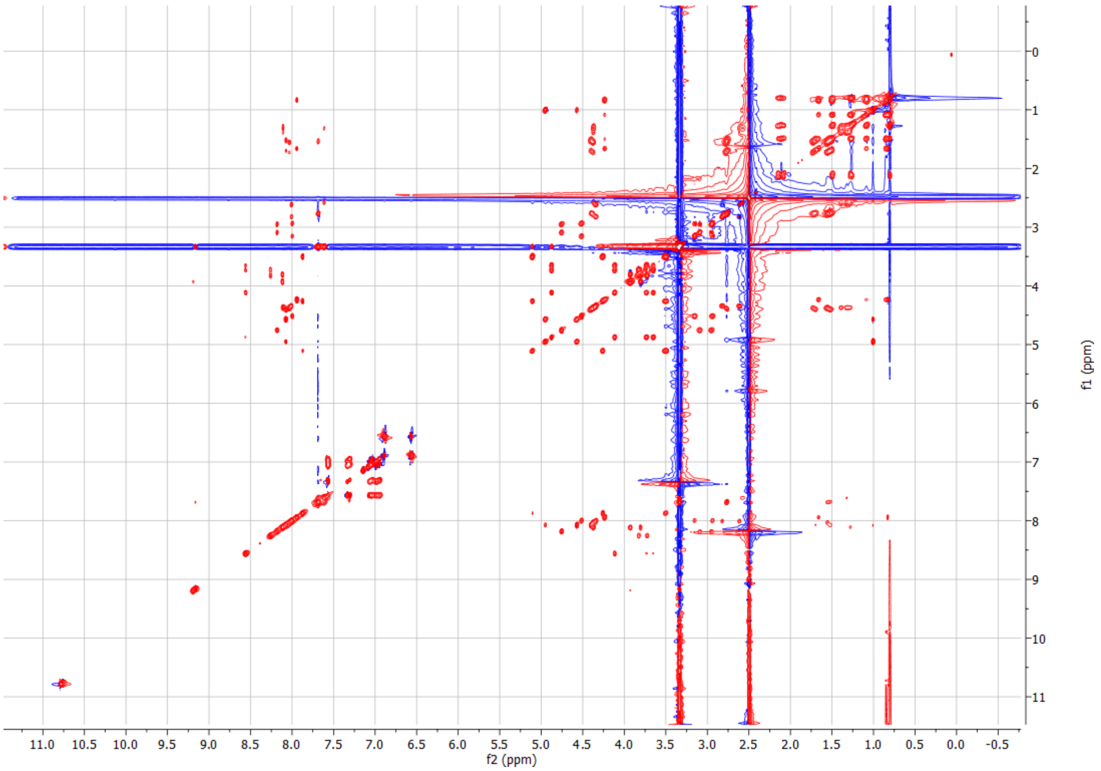

Figure S12. ^{1}H-^{1}H-TOCSY-NMR (d_6-DMSO) of relacidine A **(3a)**

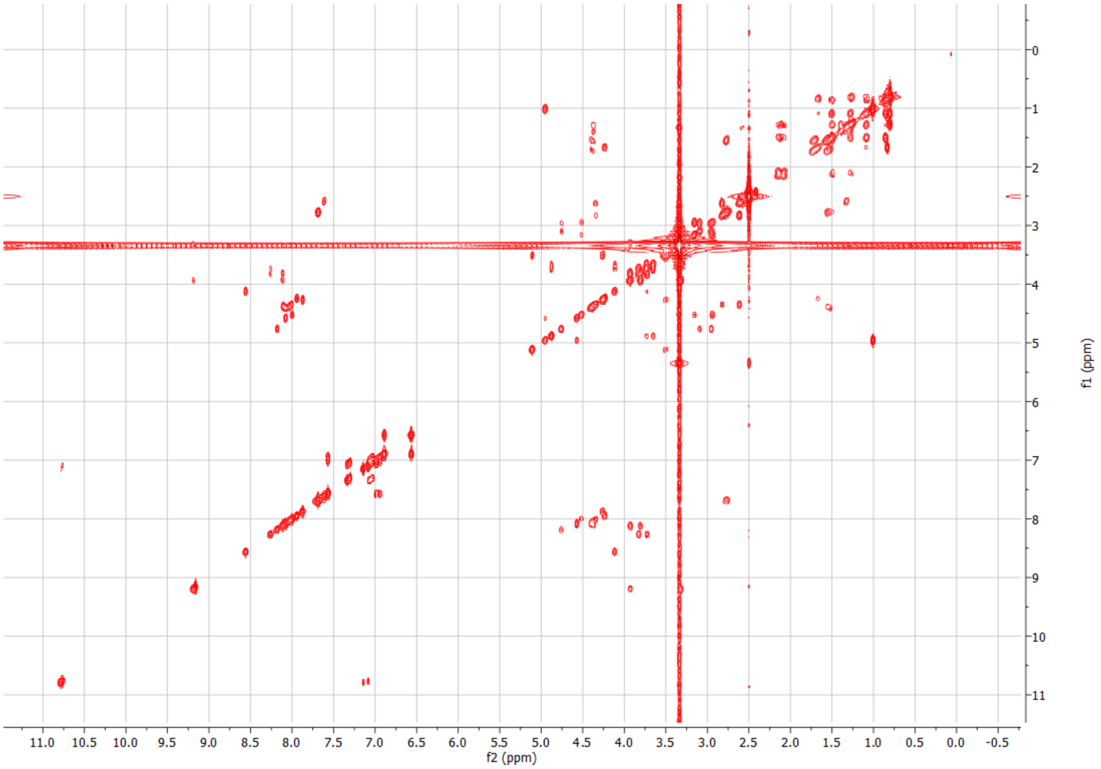

Figure S13. ^{1}H-^{1}H-COSY-NMR (d_6-DMSO) of relacidine A **(3a)**

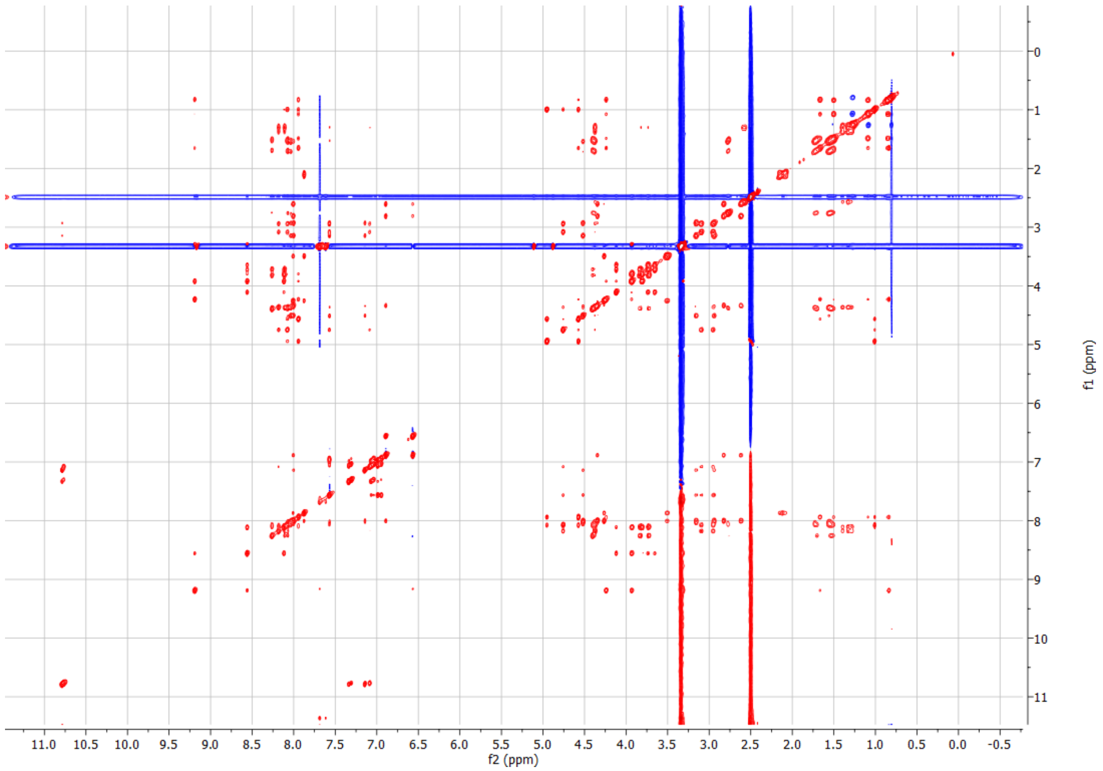

Figure S14. ^{1}H-^{1}H-NOESY-NMR (d_6-DMSO) of relacidine A **(3a)**

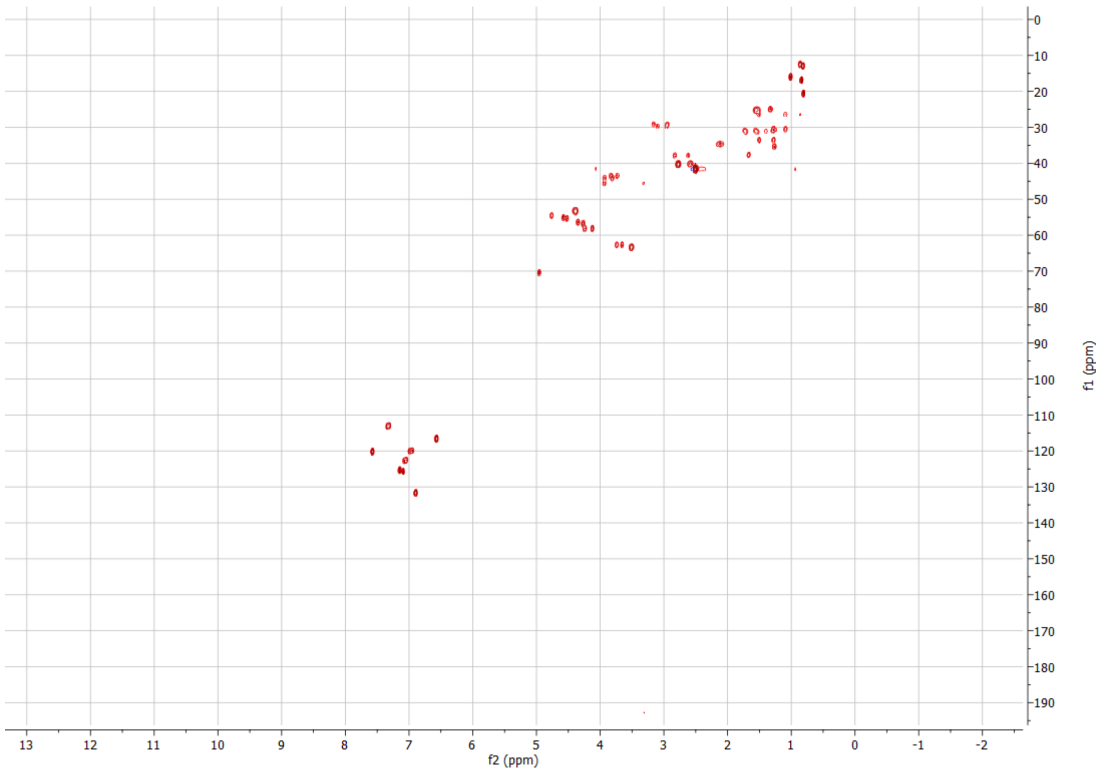

Figure S15. ^{13}C-^{1}H-HSQC-NMR (d_6-DMSO) of relacidine A **(3a)**

Table S5. ^{1}H-NMR (d_6-DMSO) characterization of D-Ser12-relacidine A **(3b)**

Residue	-NH	Hα	Hβ	Hγ	Hδ	Hε
D-Ser1	7.89 (1H, d, *J* = 7.3 Hz)	4.27 (1H, q, *J* = 6.5 Hz)	3.51 (2H, m)	5.14 (1H, br s)		
D-Tyr2	8.02 (1H, m)	4.34 (1H, m)	2.83 (1H, dd, *J* = 14.2, 4.3 Hz) & 2.62 (1H, m)	Phenol: 9.18 (1H, s), 6.89 (2H, d, *J* = 8.6 Hz), 6.57 (2H, d, *J* = 8.4 Hz)		
D-Trp3	8.01 (1H, m)	4.52 (1H, m)	3.16 (1H, m) & 2.95 (1H, m)	Indole: 10.80 (1H, s), 7.64 (1H, m), 7.34 (1H, d, *J* = 8.1 Hz), 7.15 (1H, d, *J* = 2.4 Hz), 7.07 (1H, t, *J* = 7.5 Hz), 6.99 (1H, t, *J* = 7.4 Hz)		
D-Orn4	8.02 (1H, m)	4.38 (1H, m)	1.73 (1H, m) & 1.56 (1H, m)	1.56 (2H, m)	2.78 (2H, m)	7.75 (2H, br m)
Orn5	8.09 (1H, d, *J* = 8.4 Hz)	4.41 (1H, m)	1.71 (1H, m) & 1.53 (1H, m)	1.53 (2H, m)	2.77 (2H, m)	7.75 (2H, br m)
Gly6	8.24 (1H, t, *J* = 5.6 Hz)	3.83 (1H, m) & 3,75 (1H, m)				
D-Orn7	8.04 (1H, d, *J* = 8.5 Hz)	4.38 (1H, m)	1.36 (1H, m) & 1.20 (1H, m)	1.28 (2H, m)	2.56 (2H, m)	7.65 (2H, br m)
Trp8	8.21 (1H, d, *J* = 8.2 Hz)	4,72 (1H, m)	3.16 (1H, m) & 2.91 (1H, m)	Indole: 10.76 (1H, s), 7.57 (1H, d, *J* = 7.9 Hz), 7.31 (1H, d, *J* = 8.0 Hz), 7.14 (1H, d, *J* = 2.4 Hz), 7.04 (1H, t, *J* = 7.5 Hz), 6.96 (1H, t, *J* = 7.4 Hz)		
Thr9	7.85 (1H, d, *J* = 8.8 Hz)	4.68 (1H, dd, *J* = 8.9, 3.1 Hz)	5.19 (1H, m)	1.14 (3H, d, *J* = 6.2 Hz)		
Ile10	8.40 (1H, d, *J* = 3.7 Hz)	3.90 (1H, dd, *J* = 7.6, 3.7 Hz)	1.64 (1H, m)	1.61 (1H, m) & 1.19 (1H, m), 0.88 (3H, d, *J* = 6.8 Hz)	0.90 (3H, t, *J* = 7.3 Hz)	
Gly11	9.22 (1H, t, *J* = 5.5 Hz)	3.73 (1H, m) & 3.65 (1H, m)				
D-Ser12	7.52 (1H, t, *J* = 9.1 Hz)	4.37 (1H, m)	3.98 (1H, m) & 3.83 (1H, m)	4.87 (1H, br s)		
Gly13	7.52 (1H, t, *J* = 9.1 Hz)	4.50 (1H, m) & 3.39 (1H, under H_2O)				
Lipid	2.12 (2H, m, O=C$\mathbf{CH_2}$), 1.50 (2H, O=CCH$_2$$\mathbf{CH_2}$), 1.27 (5H, -$\mathbf{CH_2CH}(CH)_3$$\mathbf{CH_2}CH_3$), 1.09 (2H, -$\mathbf{CH_2}CH_3$), 0.82 (6H, -CH($\mathbf{CH_3}$)CH$_2$$\mathbf{CH_3}$)					

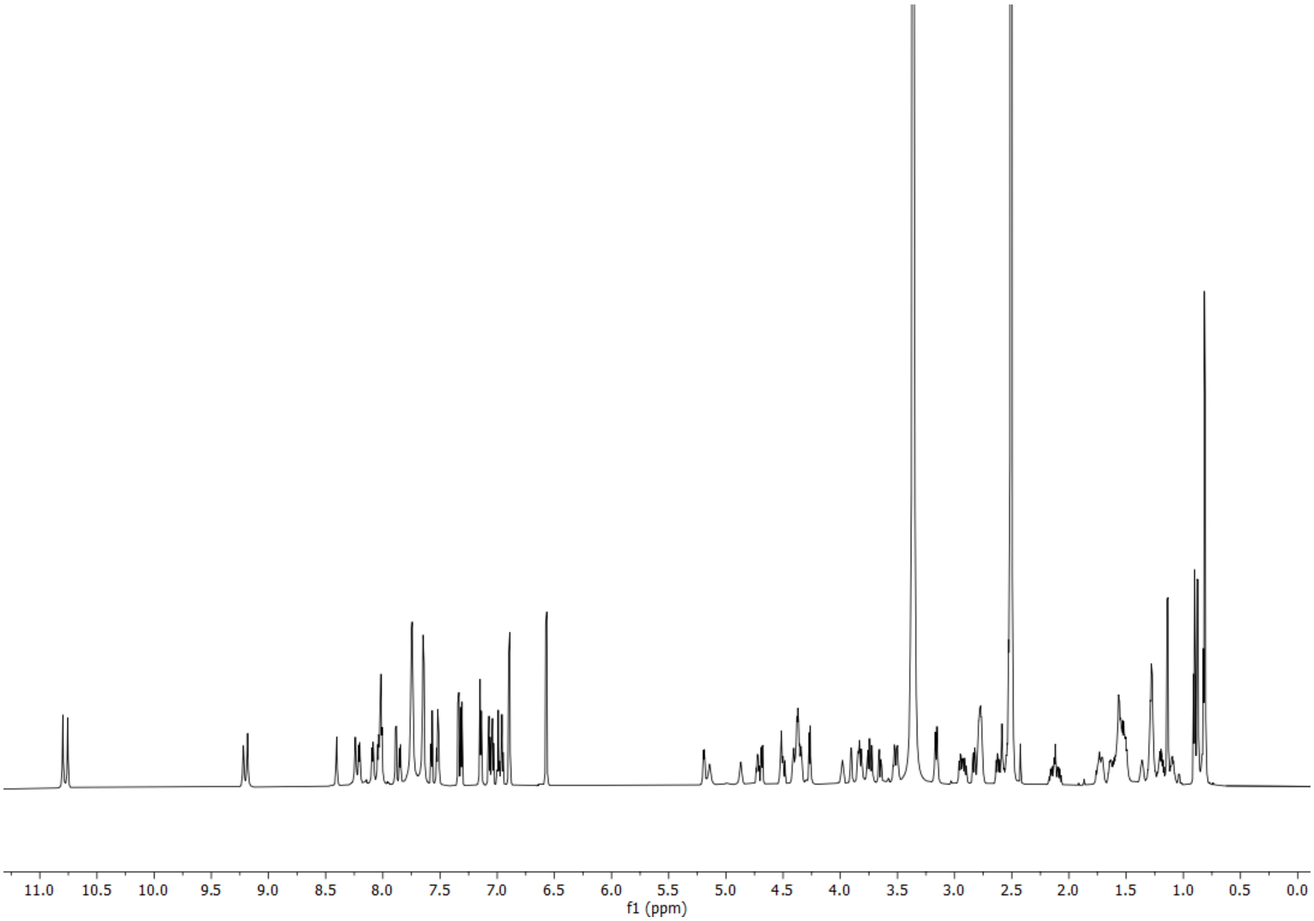

Figure S16. ^{1}H-NMR (d_6-DMSO) of D-Ser12-relacidine A **(3b)**

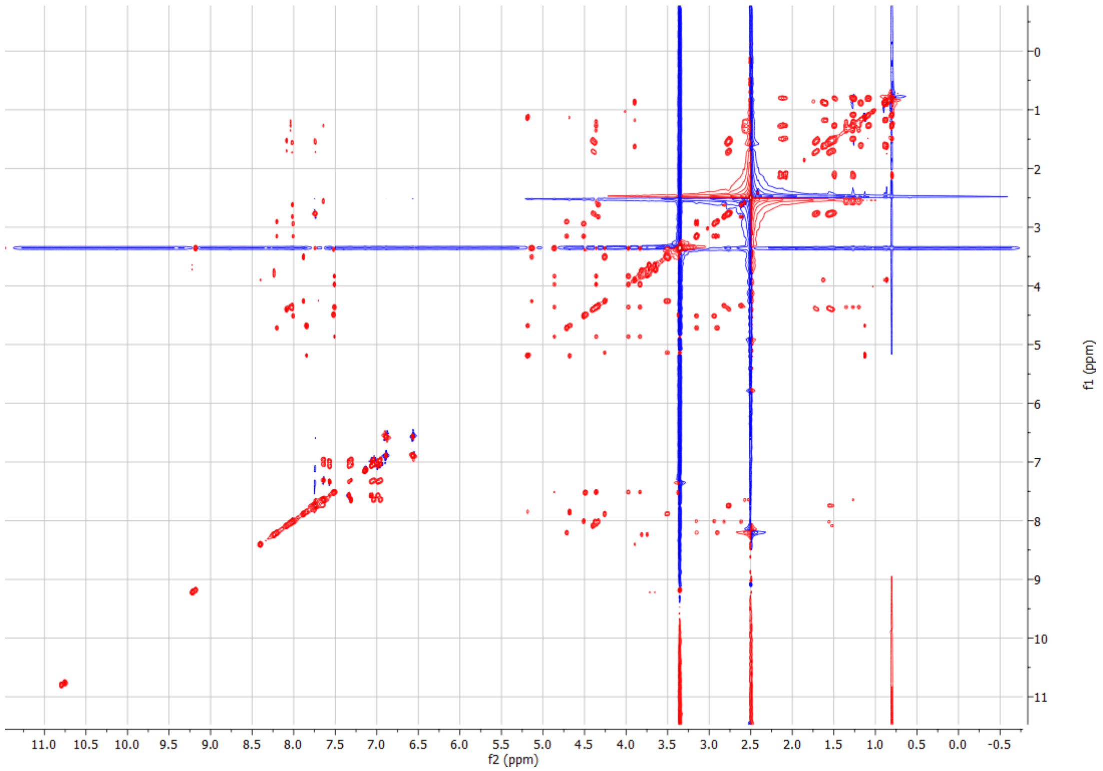

Figure S17. ^{1}H-^{1}H-TOCSY-NMR (d_6-DMSO) of D-Ser12-relacidine A **(3b)**

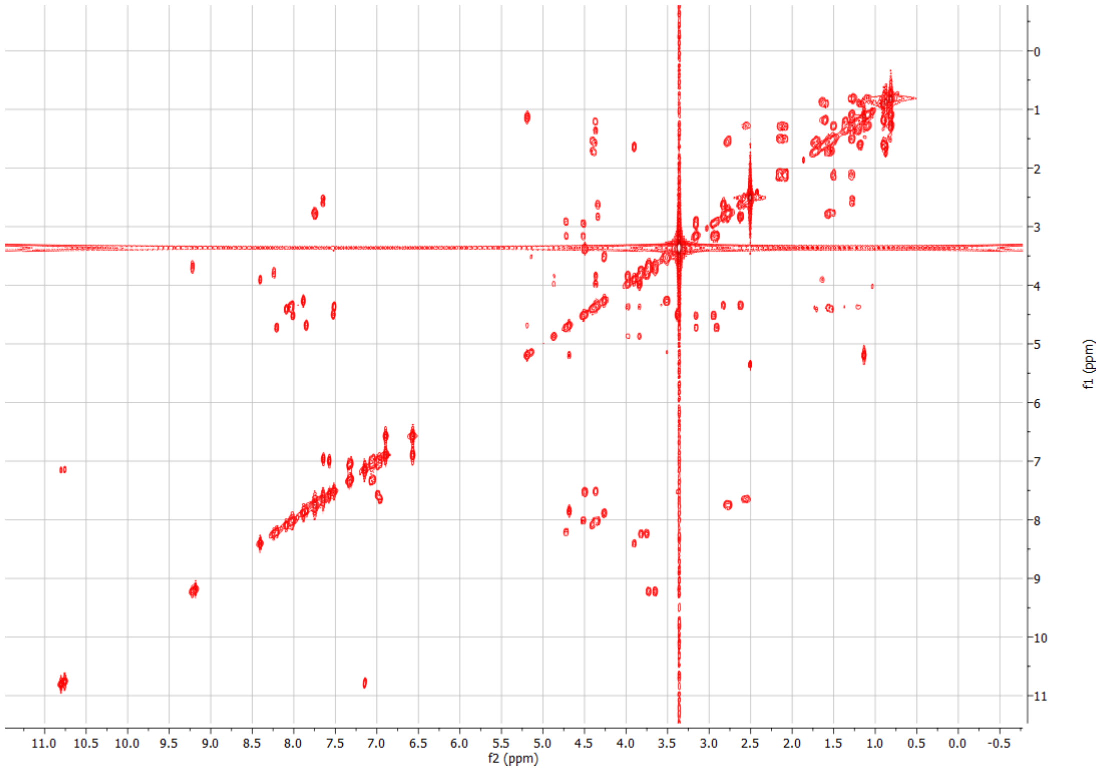

Figure S18. ^{1}H-^{1}H-COSY-NMR (d_6-DMSO) of D-Ser12-relacidine A **(3b)**

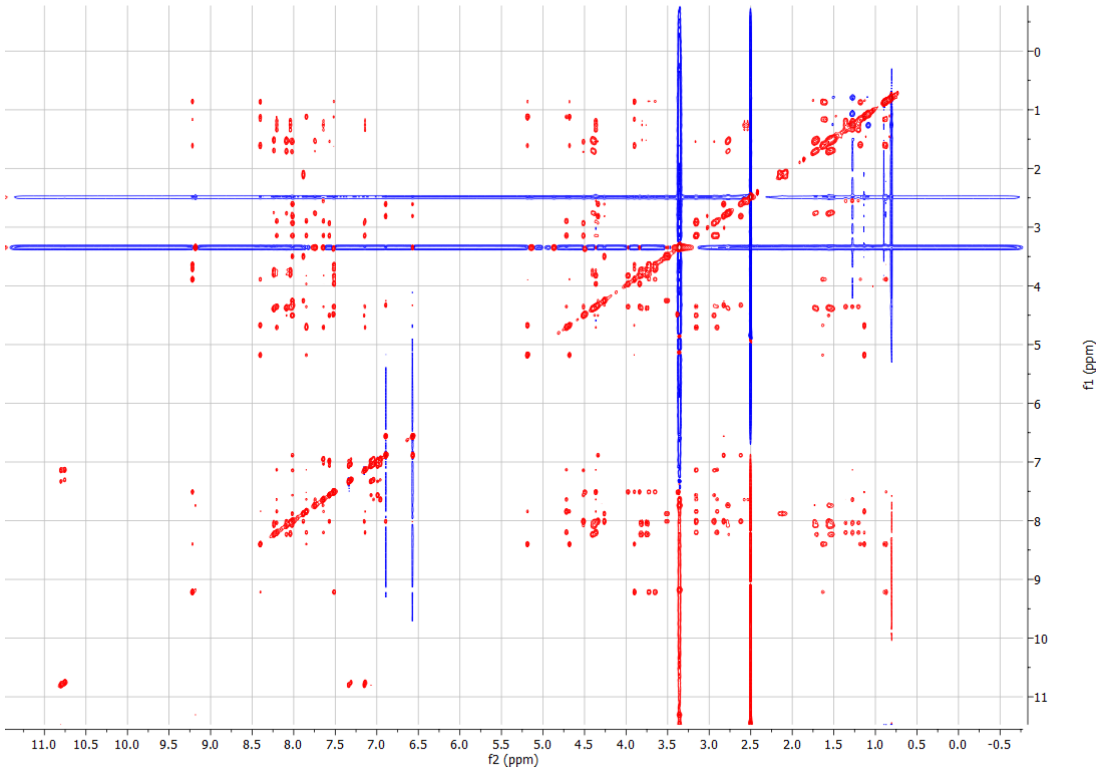

Figure S19. ^{1}H-^{1}H-NOESY-NMR (d_6-DMSO) of D-Ser12-relacidine A **(3b)**

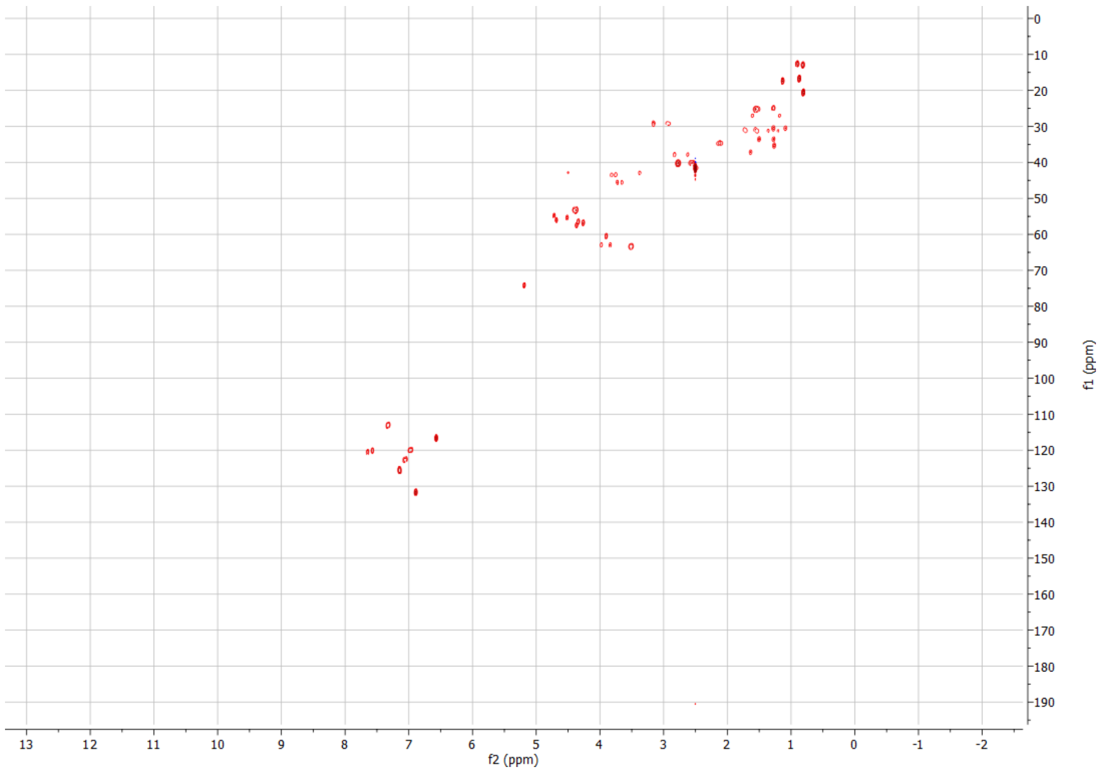

Figure S20. ^{13}C-^{1}H-HSQC-NMR (d_6-DMSO) of D-Ser12-relacidine A **(3b)**

Table S6. ^{1}H-NMR (d_6-DMSO) characterization of relacidine B **(4a)**

Residue	-NH	Hα	Hβ	Hγ	Hδ	Hε
D-Ser1	7.88 (1H, d, *J* = 7.4 Hz)	4.27 (1H, m)	3.51 (2H, m)	5.13 (1H, br s)		
D-Tyr2	8.02 (1H, m)	4.35 (1H, m)	2.83 (1H, m) & 2.62 (1H, m)	Phenol: 9.17 (1H, m), 6.90 (2H, d, *J* = 8.5 Hz), 6.57 (2H, d, *J* = 8.4 Hz)		
D-Trp3	8.01 (1H, m)	4.52 (1H, m)	3.16 (1H, dd, *J* = 15.0, 4.5 Hz) & 2.95 (1H, m)	Indole: 10.79 (1H, s), 7.57 (1H, d, *J* = 8.0 Hz), 7.34 (1H, d, *J* = 8.1 Hz), 7.15 (1H, d, *J* = 2.4 Hz), 7.07 (1H, m), 6.99 (1H, m)		
D-Orn4	8.03 (1H, m)	4.38 (1H, m)	1.73 (1H, m) & 1.56 (1H, m)	1.56 (2H, m)	2.78 (2H, m)	7.72 (2H, br m)
Orn5	8.09 (1H, d, *J* = 8.3 Hz)	4.41 (1H, m)	1.71 (1H, m) & 1.53 (1H, m)	1.53 (2H, m)	2.77 (2H, m)	7.72 (2H, br m)
Gly6	8.26 (1H, t, *J* = 5.6 Hz)	3.84 (1H, m) & 3.74 (1H, dd, *J* = 16.9, 5.1 Hz)				
D-Orn7	8.11 (1H, d, *J* = 8.4 Hz)	4.38 (1H, m)	1.41 (1H, m) & 1.29 (1H, m)	1.34 (2H, m)	2.59 (2H, m)	7.65 (2H, br m)
Trp8	8.22 (1H, d, *J* = 8.1 Hz)	4,75 (1H, m)	3.11 (1H, dd, *J* = 14.7, 5.2 Hz) & 2.96 (1H, m)	Indole: 10.78 (1H, s), 7.59 (1H, d, *J* = 7.9 Hz), 7.31 (1H, d, *J* = 8.0 Hz), 7.11 (1H, d, *J* = 2.4 Hz), 7.04 (1H, m), 6.96 (1H, m)		
Thr9	8.06 (1H, d, *J* = 8.1 Hz)	4.64 (1H, dd, *J* = 8.1, 4.0 Hz)	5.00 (1H, m)	0.99 (3H, d, *J* = 6.5 Hz)		
Ile10	7.72 (1H, m)	4.17 (1H, t, *J* = 8.5 Hz)	1.66 (1H, m)	1.54 (1H, m) & 1.11 (1H, m), 0.86 (3H, m)	0.87 (3H, m)	
Gly11	9.17 (1H, t, *J* = 6.4 Hz)	3.91 (1H, dd, *J* = 14.3, 4.7 Hz) & 3.36 (1H, under H_2O)				
Ser12	8.46 (1H, d, *J* = 7.8 Hz)	4.10 (1H, m)	3.81 (1H, m) & 3.69 (1H, dd, *J* = 11.0, 3.4 Hz)	4.88 (1H, br s)		
Ala13	7.76 (1H, d, *J* = 8.6 Hz)	4.39 (1H, m)	1.43 (3H, d, *J* = 7.2 Hz)			
Lipid	2.12 (2H, m, O=CC**H**$_2$), 1.50 (2H, O=CCH$_2$C**H**$_2$), 1.27 (5H, -**CH**$_2$**CH**(CH)$_3$C**H**$_2$CH$_3$), 1.09 (2H, -C**H**$_2$CH$_3$), 0.82 (6H, -CH(C**H**$_3$)CH$_2$C**H**$_3$)					

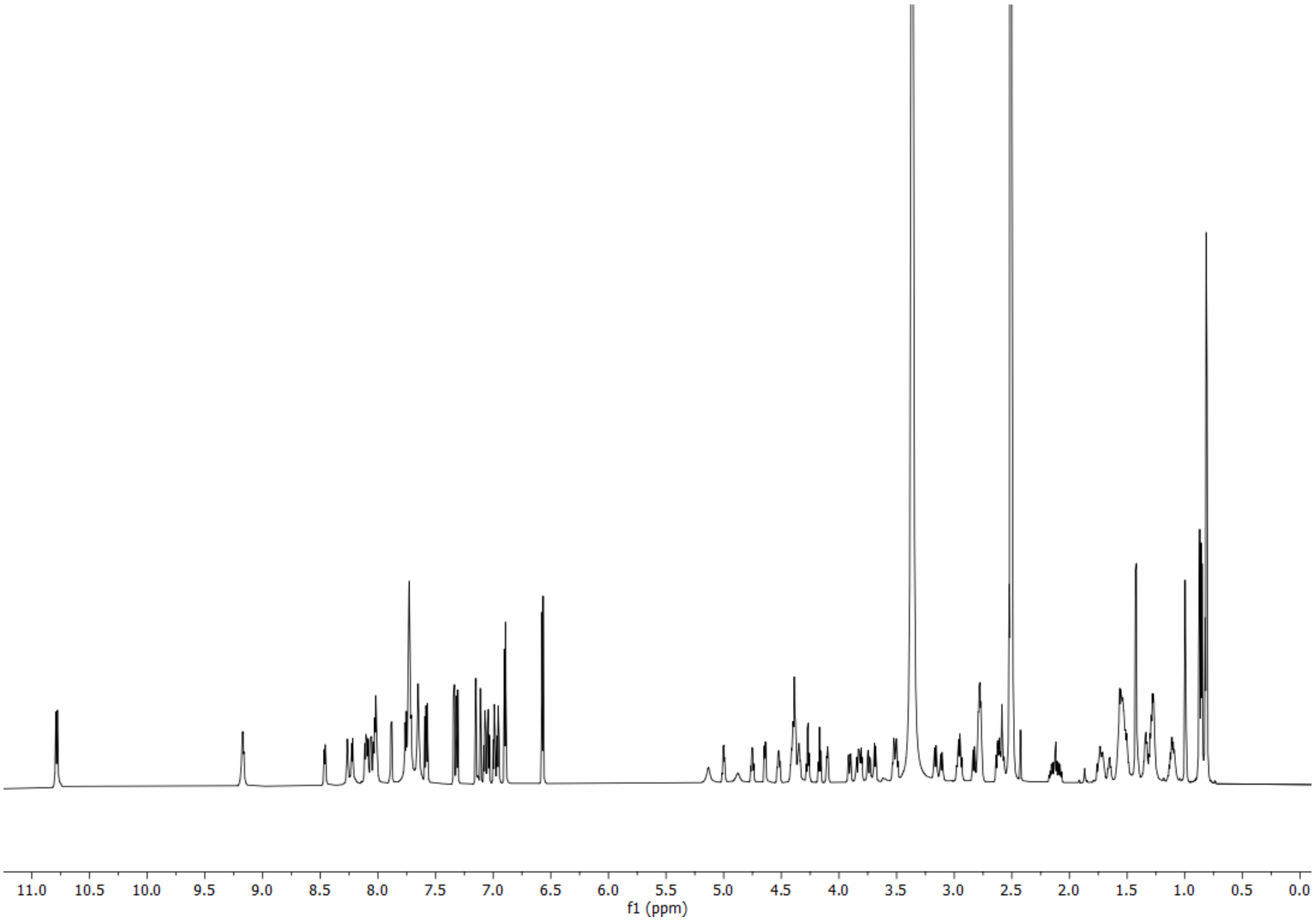

Figure S21. ^{1}H-NMR (d_6-DMSO) of relacidine B **(4a)**

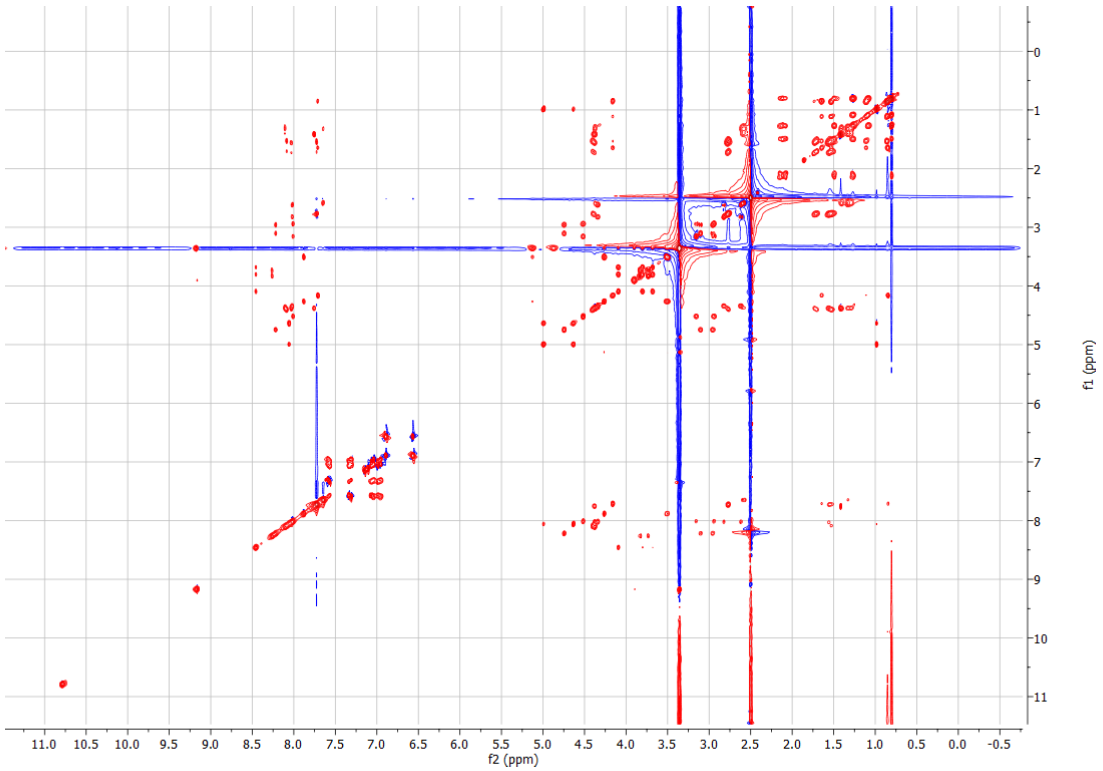

Figure S22. ^{1}H-^{1}H-TOCSY-NMR (d_6-DMSO) of relacidine B **(4a)**

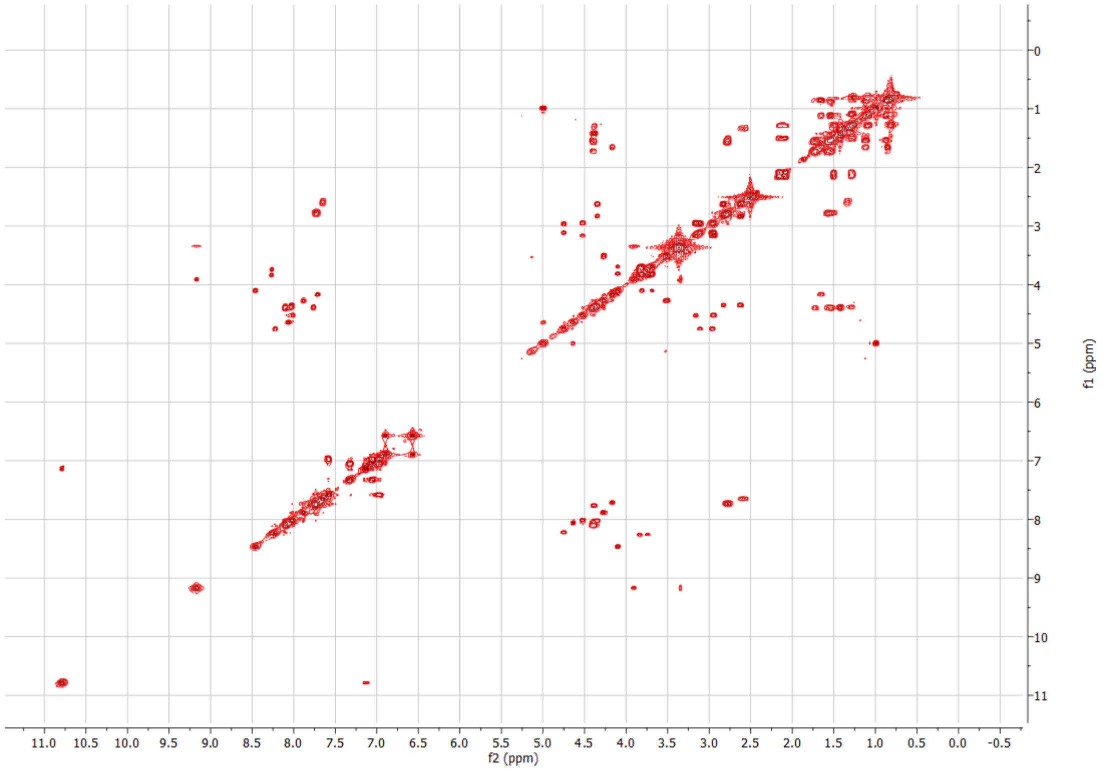

Figure S23. ^{1}H-^{1}H-COSY-NMR (d_6-DMSO) of relacidine B **(4a)**

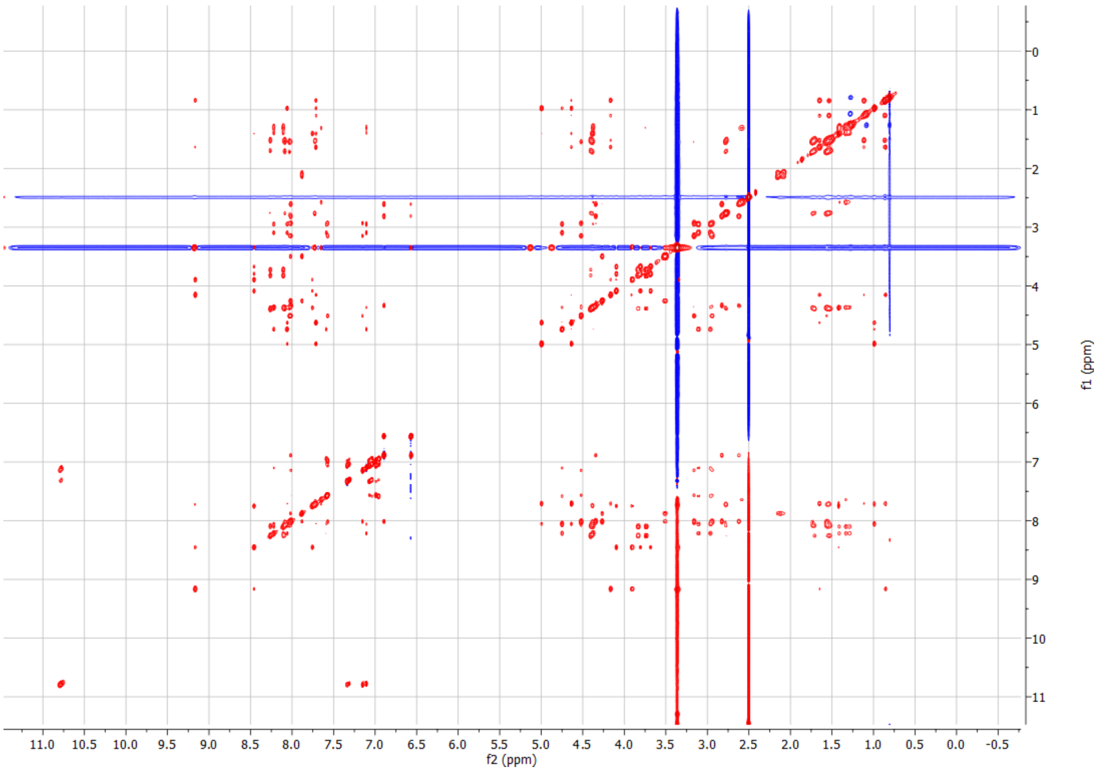

Figure S24. ^{1}H-^{1}H-NOESY-NMR (d_6-DMSO) of relacidine B **(4a)**

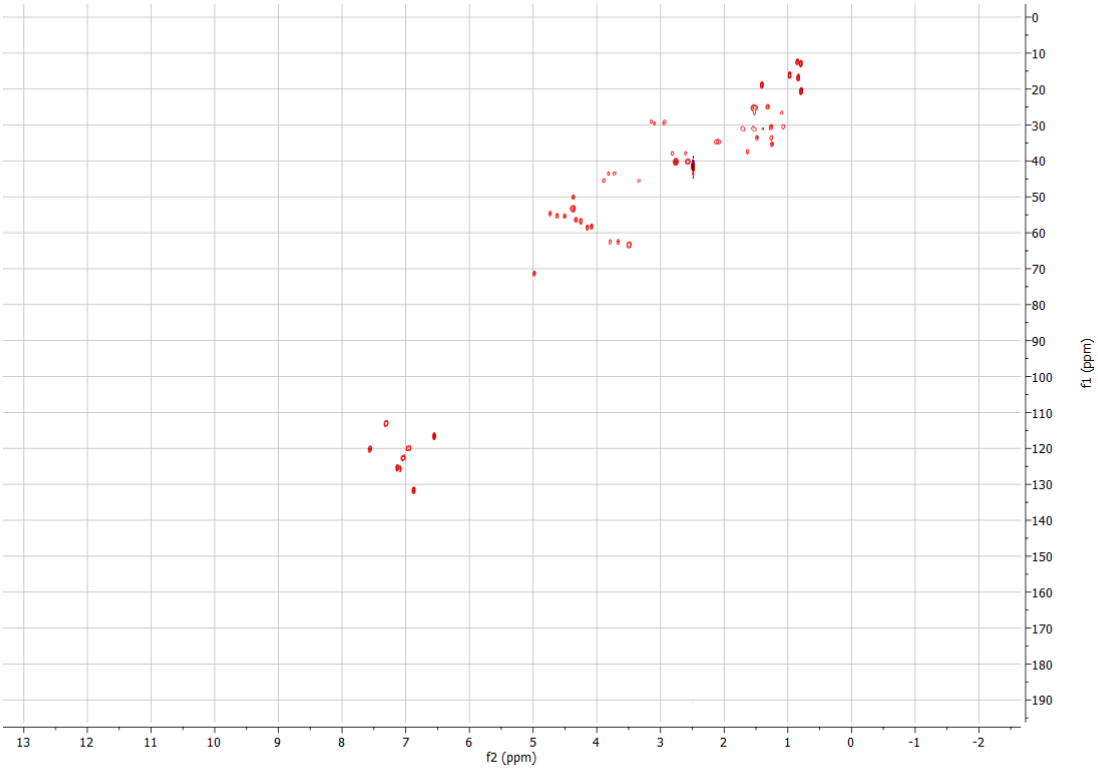

Figure S25. ^{13}C-^{1}H-HSQC-NMR (d_6-DMSO) of relacidine B **(4a)**

Table S7. ^{1}H-NMR (d_6-DMSO) characterization of D-Ala13, L-Ser12-relacidine B **(4b)**

Residue	-NH	Hα	Hβ	Hγ	Hδ	Hε
D-Ser1	7.88 (1H, d, *J* = 7.4 Hz)	4.27 (1H, m)	3.51 (2H, m)	5.14 (1H, br s)		
D-Tyr2	8.02 (1H, m)	4.35 (1H, m)	2.83 (1H, dd, *J* = 14.2, 4.3 Hz) & 2.62 (1H, m)	Phenol: 9.18 (1H, s), 6.90 (2H, d, *J* = 8.5 Hz), 6.57 (2H, d, *J* = 8.4 Hz)		
D-Trp3	8.01 (1H, m)	4.53 (1H, m)	3.16 (1H, dd, *J* = 15.0, 4.5 Hz) & 2.95 (1H, m)	Indole: 10.79 (1H, s), 7.58 (1H, d, *J* = 2.9 Hz), 7.34 (1H, d, *J* = 8.1 Hz), 7.15 (1H, d, *J* = 2.4 Hz), 7.07 (1H, t, *J* = 7.5 Hz), 6.99 (1H, t, *J* = 7.4 Hz)		
D-Orn4	8.03 (1H, m)	4.38 (1H, m)	1.73 (1H, m) & 1.56 (1H, m)	1.56 (2H, m)	2.78 (2H, m)	7.74 (2H, br m)
Orn5	8.09 (1H, d, *J* = 8.2 Hz)	4.41 (1H, m)	1.71 (1H, m) & 1.53 (1H, m)	1.53 (2H, m)	2.77 (2H, m)	7.74 (2H, br m)
Gly6	8.27 (1H, t, *J* = 5.6 Hz)	3.84 (1H, dd, *J* = 16.9, 6.0 Hz) & 3.72 (1H, dd, *J* = 16.8, 5.1 Hz)				
D-Orn7	8.11 (1H, d, *J* = 8.5 Hz)	4.38 (1H, m)	1.42 (1H, m) & 1.29 (1H, m)	1.35 (2H, m)	2.59 (2H, m)	7.66 (2H, br m)
Trp8	8.21 (1H, d, *J* = 8.1 Hz)	4,76 (1H, m)	3.13 (1H, dd, *J* = 14.6, 5.0 Hz) & 2.97 (1H, m)	Indole: 10.79 (1H, s), 7.57 (1H, d, *J* = 3.0 Hz), 7.31 (1H, d, *J* = 8.0 Hz), 7.10 (1H, d, *J* = 2.4 Hz), 7.04 (1H, t, *J* = 7.5 Hz), 6.95 (1H, t, *J* = 7.4 Hz)		
Thr9	7.95 (1H, d, *J* = 8.4 Hz)	4.58 (1H, dd, *J* = 8.4, 3.9 Hz)	5.07 (1H, m)	1.05 (3H, d, *J* = 6.5 Hz)		
Ile10	7.80 (1H, d, *J* = 9.4 Hz)	4.27 (1H, m)	1.74 (1H, m)	1.42 (1H, m) & 1.10 (1H, m), 0.84 (3H, m)	0.84 (3H, m)	
Gly11	8.96 (1H, t, *J* = 5.8 Hz)	3.95 (1H, dd, *J* = 14.0, 5.1 Hz) & 3.31 (1H, dd, *J* = 14.0, 6.4 Hz)				
Ser12	8.66 (1H, d, *J* = 7.8 Hz)	4.03 (1H, m)	3.65 (2H, m)	4.89 (1H, br s)		
D-Ala13	7.98 (1H, d, *J* = 7.5 Hz)	4.18 (1H, p, *J* = 7.1 Hz)	1.26 (3H, d, *J* = 7.0 Hz)			
Lipid	2.12 (2H, m, $O{=}C\mathbf{CH_2}$), 1.50 (2H, $O{=}CCH_2\mathbf{CH_2}$), 1.27 (5H, $-\mathbf{CH_2CH}(CH)_3\mathbf{CH_2}CH_3$), 1.09 (2H, $-\mathbf{CH_2}CH_3$), 0.82 (6H, $-CH(\mathbf{CH_3})CH_2\mathbf{CH_3}$)					

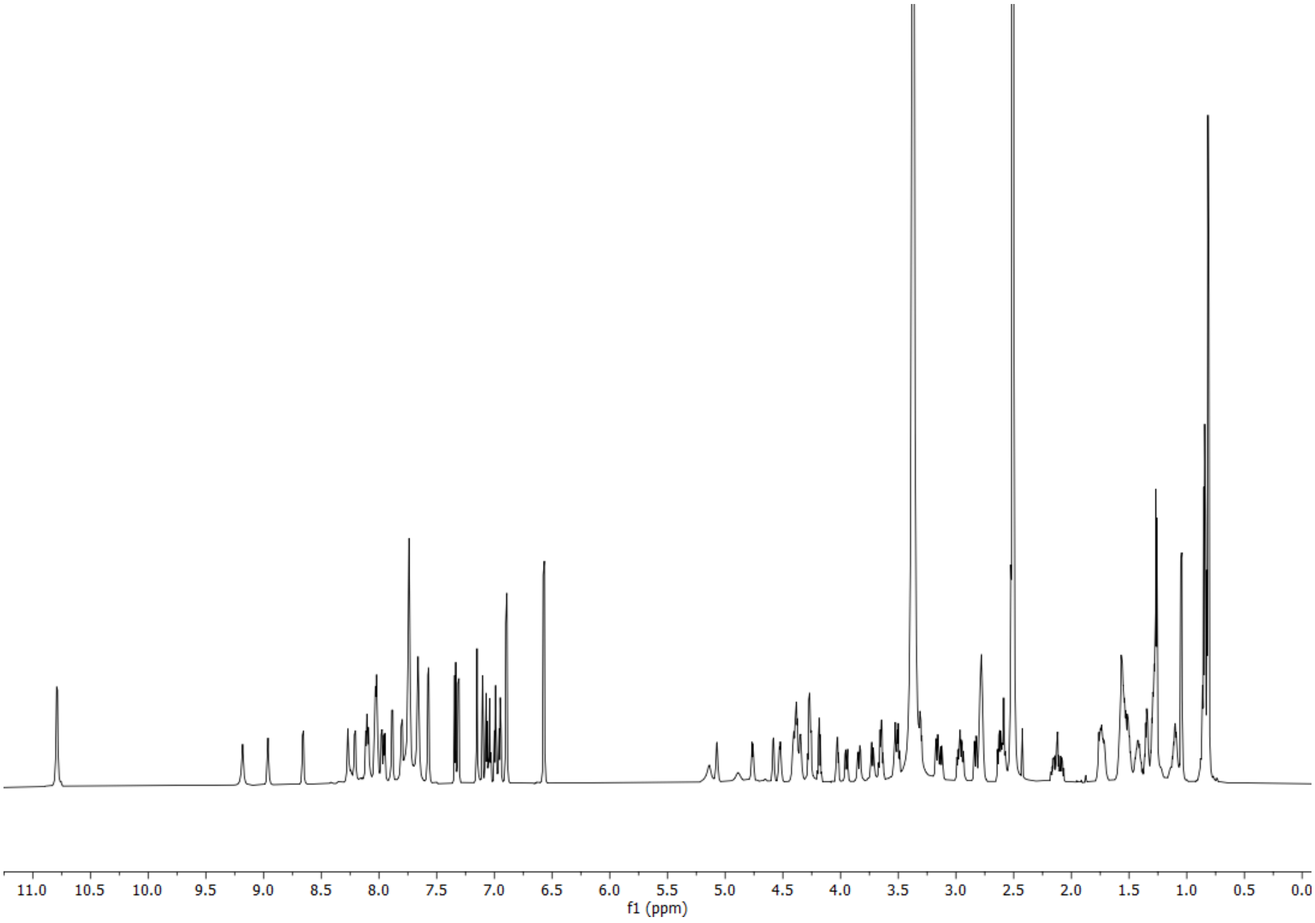

Figure S26. ^{1}H-NMR (d_6-DMSO) of D-Ala13, L-Ser12-relacidine B **(4b)**

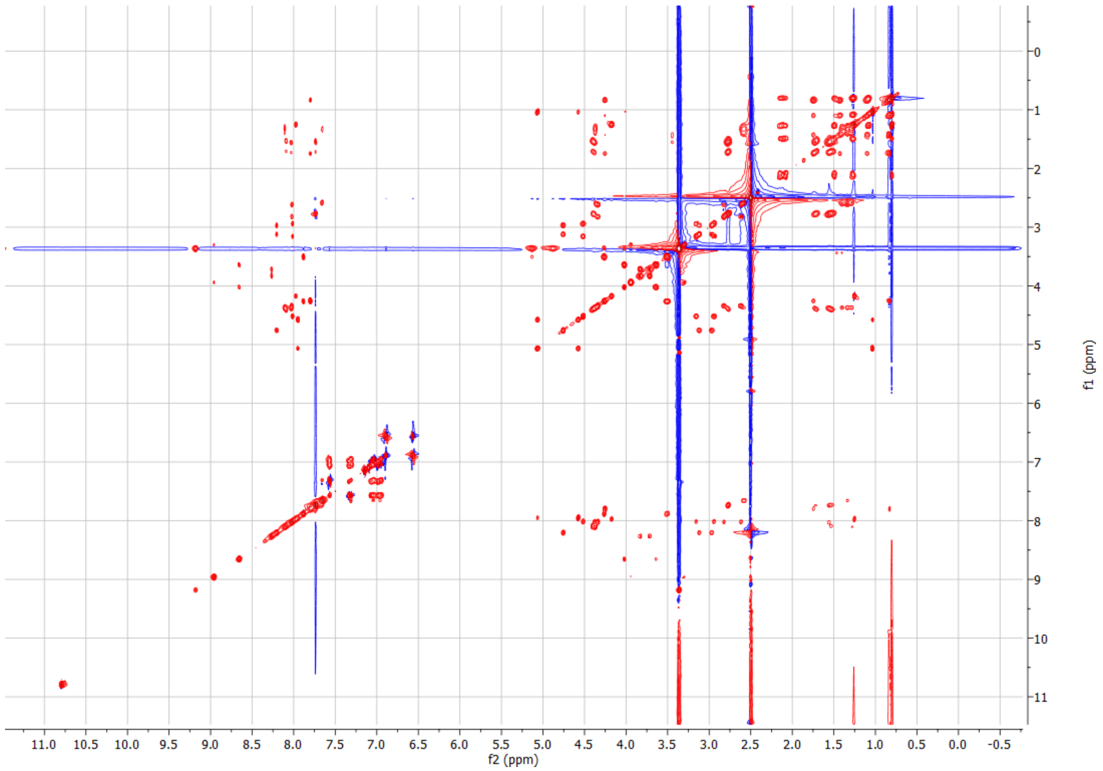

Figure S27. ^{1}H-^{1}H-TOCSY-NMR (d_6-DMSO) of D-Ala13, L-Ser12-relacidine B **(4b)**

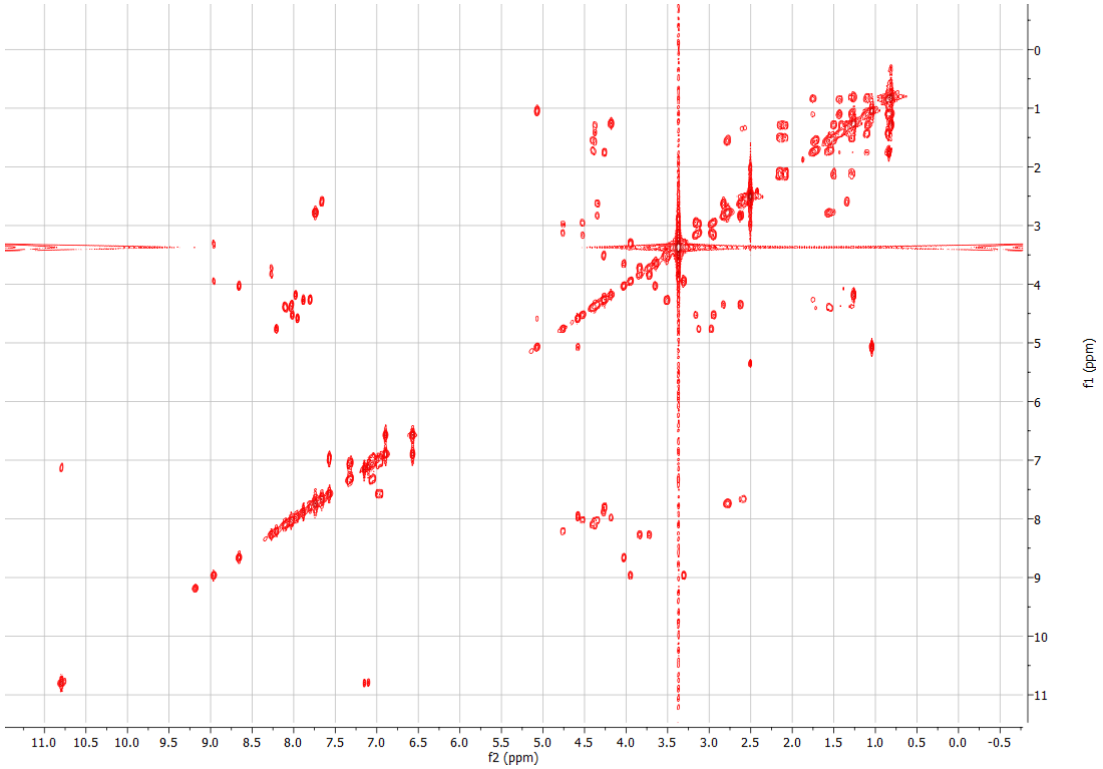

Figure S28. ^{1}H-^{1}H-COSY-NMR (d_6-DMSO) of D-Ala13, L-Ser12-relacidine B **(4b)**

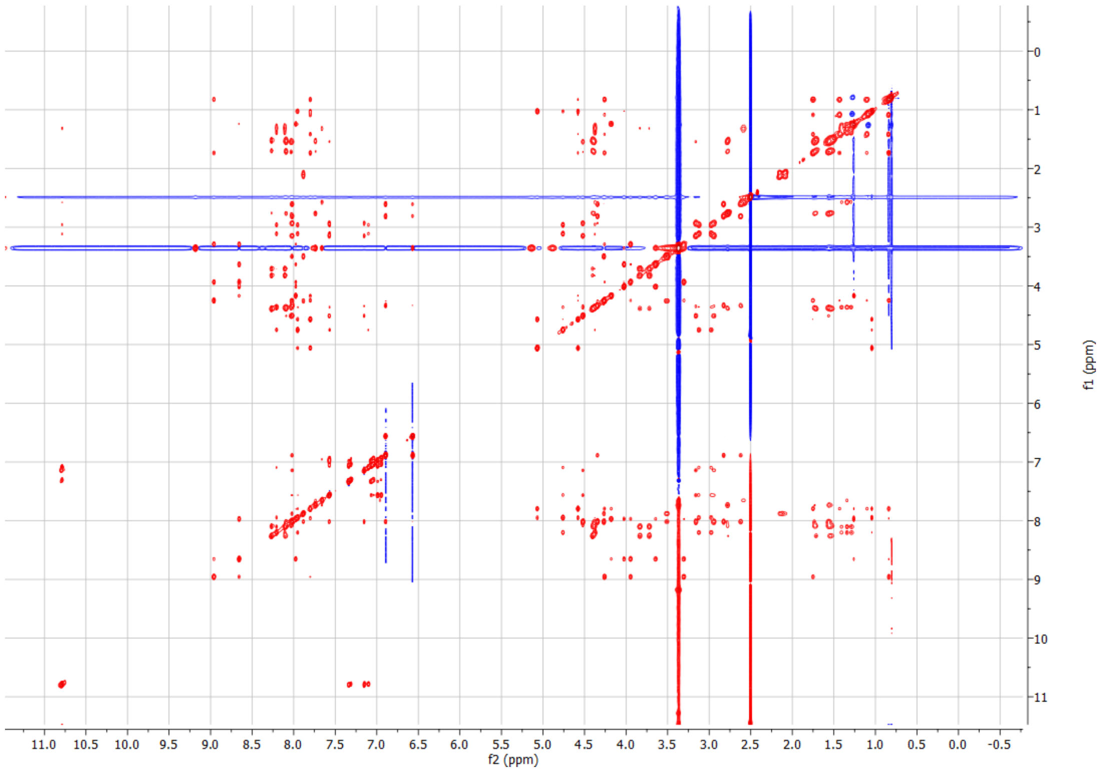

Figure S29. ^{1}H-^{1}H-NOESY-NMR (d_6-DMSO) of D-Ala13, L-Ser12-relacidine B **(4b)**

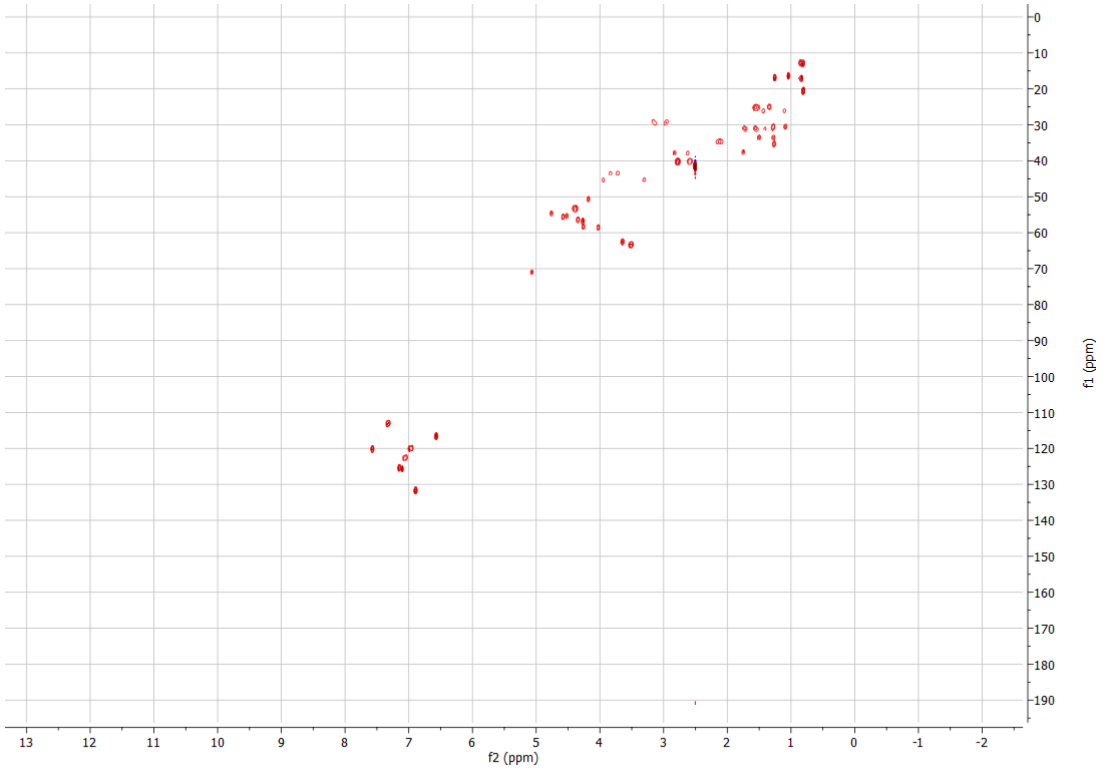

Figure S30. ^{13}C-^{1}H-HSQC-NMR (d_6-DMSO) of D-Ala13, L-Ser12-relacidine B **(4c)**

Table S8. ^{1}H-NMR (d_6-DMSO) characterization of L-Ala13, D-Ser12-relacidine B **(4c)**

Residue	-NH	Hα	Hβ	Hγ	Hδ	Hε
D-Ser1	7.88 (1H, d, *J* = 7.3 Hz)	4.26 (1H, q, *J* = 6.5 Hz)	3.52 (2H, m)	5.12 (1H, t, *J* = 5.5 Hz)		
D-Tyr2	8.02 (1H, m)	4.35 (1H, m)	2.83 (1H, dd, *J* = 14.2, 4.3 Hz) & 2.62 (1H, dd, *J* = 14.2, 9.5 Hz)	Phenol: 9.17 (1H, s), 6.89 (2H, m), 6.57 (2H, m)		
D-Trp3	8.00 (1H, m)	4.51 (1H, m)	3.16 (1H, m) & 2.95 (1H, dd, *J* = 15.0, 9.4 Hz)	Indole: 10.79 (1H, s), 7.57 (1H, d, *J* = 7.9 Hz), 7.34 (1H, d, *J* = 8.0 Hz), 7.15 (1H, m), 7.07 (1H, m), 6.99 (1H, m)		
D-Orn4	8.02 (1H, d, *J* = 8.4 Hz)	4.35 (1H, m)	1.73 (1H, m) & 1.56 (1H, m)	1.56 (2H, m)	2.77 (2H, m)	7.71 (2H, br m)
Orn5	8.08 (1H, d, *J* = 8.2 Hz)	4.41 (1H, m)	1.71 (1H, m) & 1.53 (1H, m)	1.53 (2H, m)	2.77 (2H, m)	7.71 (2H, br m)
Gly6	8.24 (1H, t, *J* = 5.6 Hz)	3.81 (1H, m) & 3.74 (1H, m)				
D-Orn7	8.04 (1H, d, *J* = 8.4 Hz)	4.38 (1H, m)	1.35 (1H, m) & 1.20 (1H, m)	1.25 (2H, m)	2.54 (2H, m)	7.61 (2H, br m)
Trp8	8.20 (1H, d, *J* = 8.2 Hz)	4,73 (1H, m)	3.16 (1H, m) & 2.90 (1H, dd, *J* = 14.7, 9.7 Hz)	Indole: 10.74 (1H, s), 7.66 (1H, d, *J* = 7.8 Hz), 7.31 (1H, d, *J* = 8.1 Hz), 7.15 (1H, m), 7.04 (1H, m), 6.96 (1H, m)		
Thr9	7.84 (1H, d, *J* = 8.9 Hz)	4.68 (1H, dd, *J* = 9.0, 2.8 Hz)	5.26 (1H, m)	1.13 (3H, d, *J* = 6.3 Hz)		
Ile10	8.39 (1H, d, *J* = 3.1 Hz)	3.82 (1H, m)	1.66 (1H, m)	1.61 (1H, m) & 1.20 (1H, m), 0.88 (3H, d, *J* = 6.7 Hz)	0.91 (3H, t, *J* = 7.3 Hz)	
Gly11	9.17 (1H, m)	3.75 (1H, m) & 3.62 (1H, m)				
D-Ser12	7.47 (1H, d, *J* = 9.0 Hz)	4.34 (1H, m)	3.99 (1H, m) & 3.84 (1H, m)	4.86 (1H, t, *J* = 6.6 Hz)		
Ala13	7.45 (1H, d, *J* = 9.2 Hz)	4.61 (1H, m)	1.18 (3H, d, *J* = 6.9 Hz)			
Lipid	2.12 (2H, m, O=CC**H₂**), 1.50 (2H, O=CCH₂C**H₂**), 1.27 (5H, -C**H₂**C**H**(CH)₃C**H₂**CH₃), 1.09 (2H, -C**H₂**CH₃), 0.82 (6H, -CH(C**H₃**)CH₂C**H₃**)					

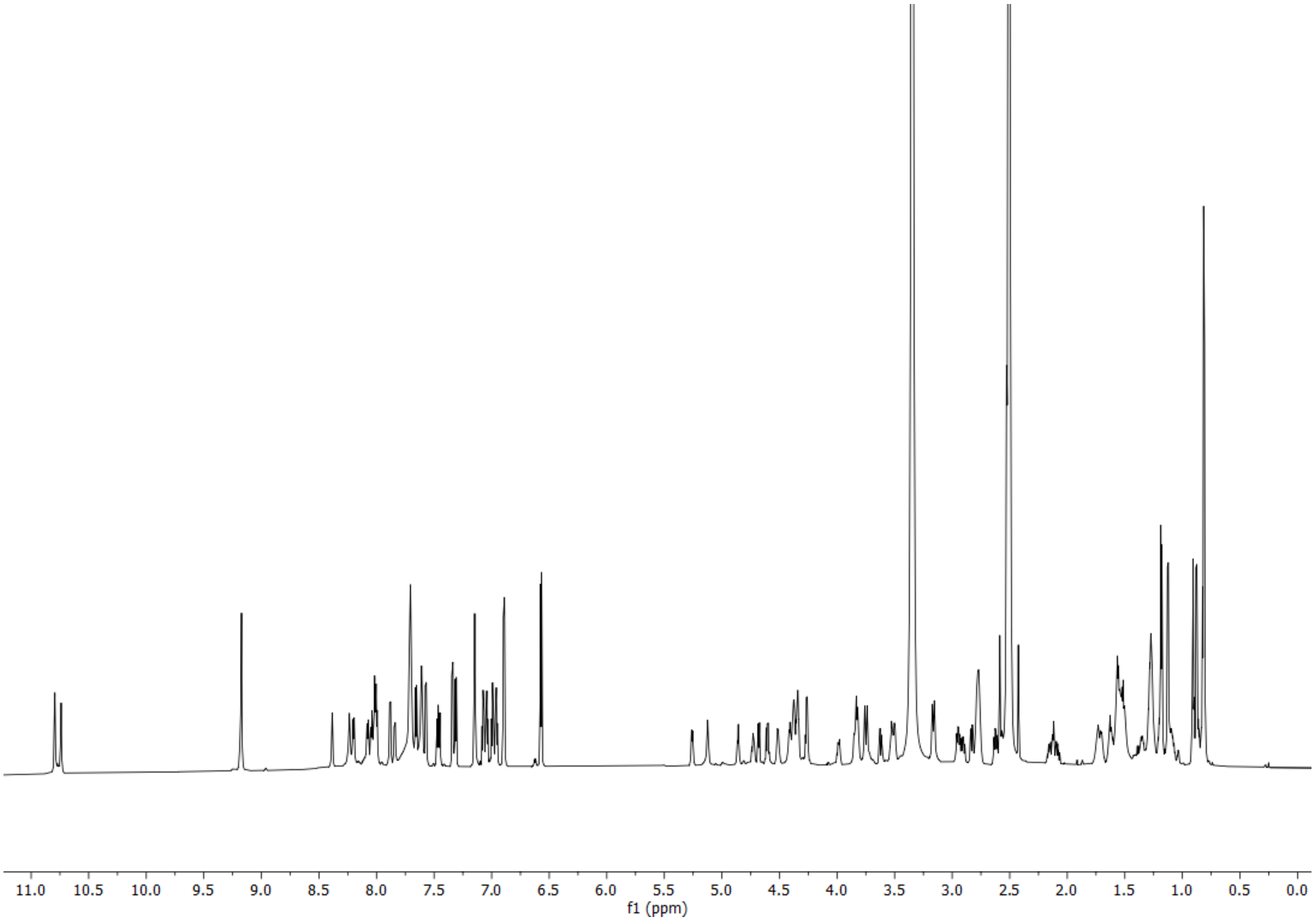

Figure S31. ^{1}H-NMR (d_6-DMSO) of L-Ala13, D-Ser12-relacidine B **(4c)**

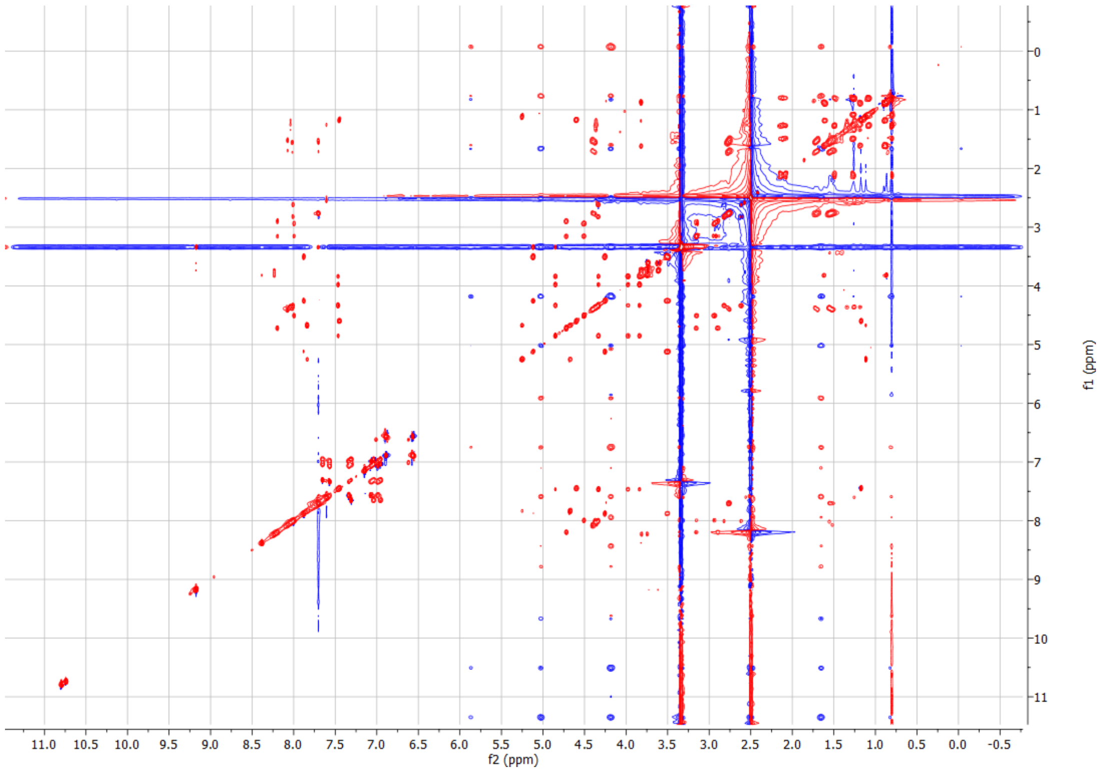

Figure S32. ^{1}H-^{1}H-TOCSY-NMR (d_6-DMSO) of L-Ala13, D-Ser12-relacidine B **(4c)**

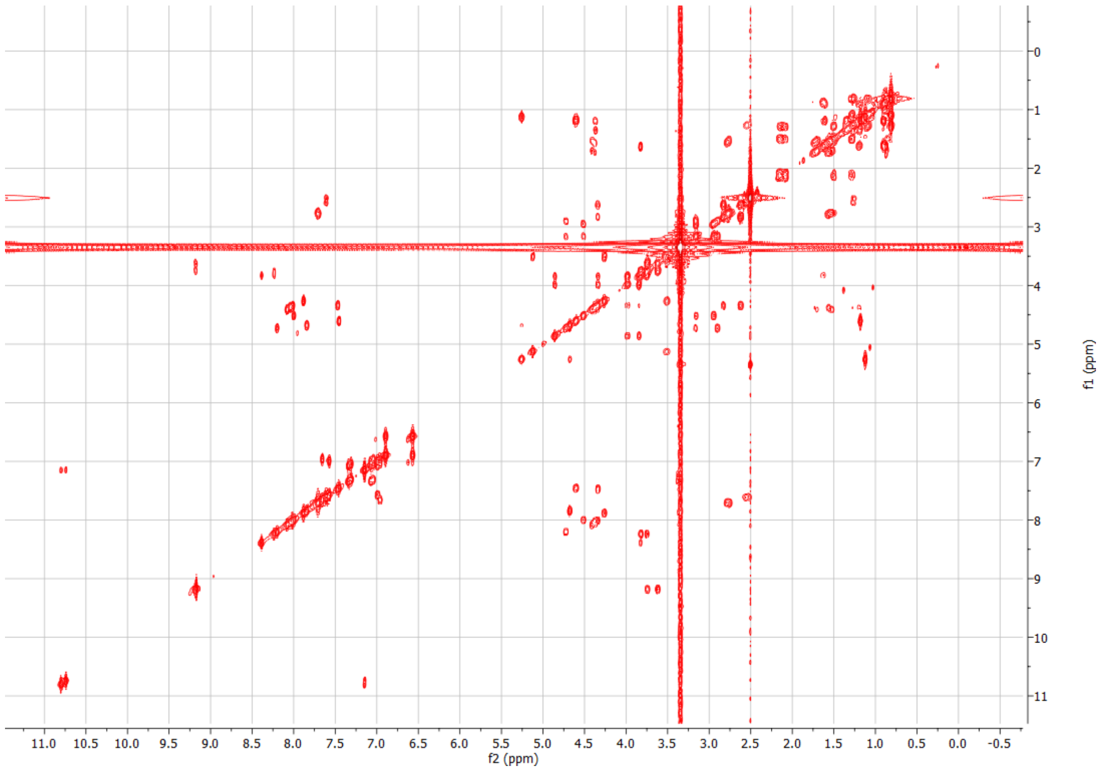

Figure S33. ^{1}H-^{1}H-COSY-NMR (d_6-DMSO) of L-Ala13, D-Ser12-relacidine B **(4c)**

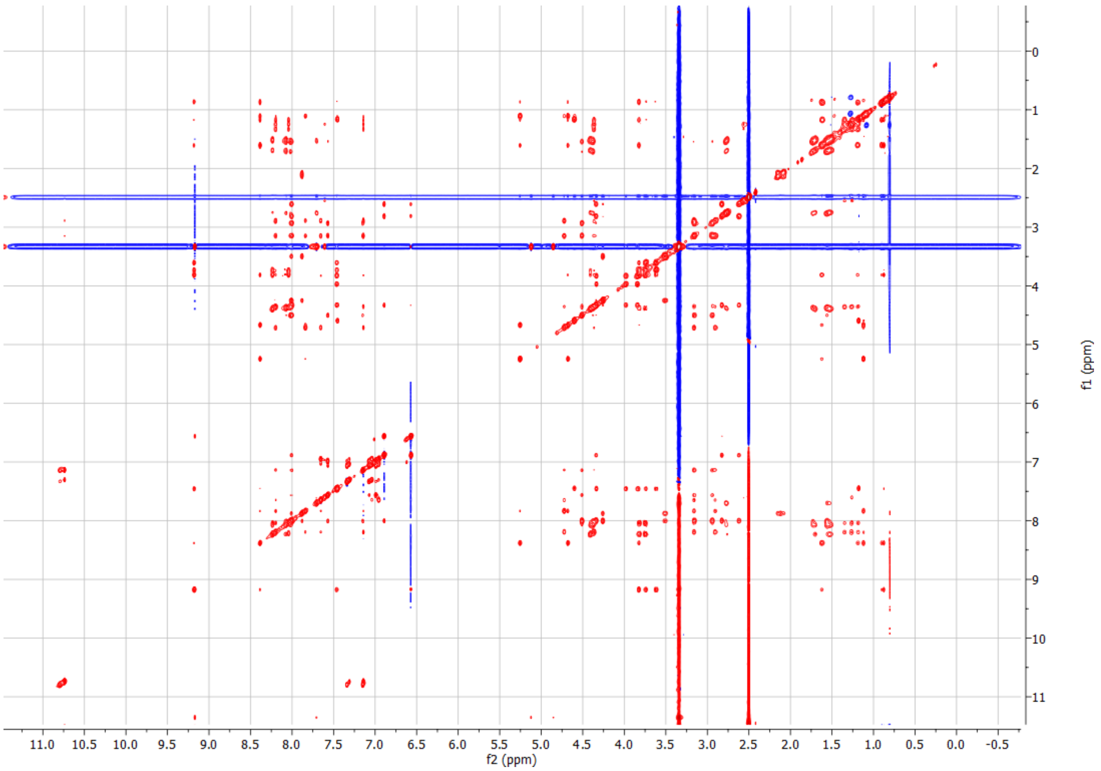

Figure S34. ^{1}H-^{1}H-NOESY-NMR (d_6-DMSO) of L-Ala13, D-Ser12-relacidine B **(4c)**

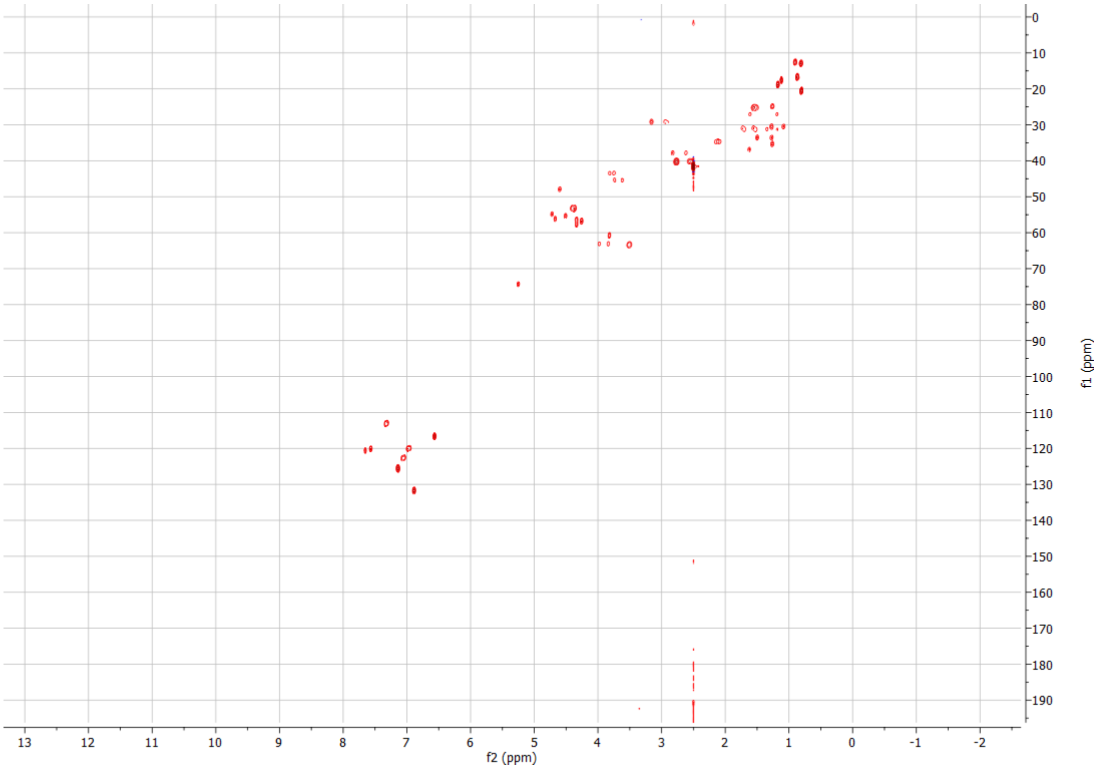

Figure S35. ^{13}C-^{1}H-HSQC-NMR (d_6-DMSO) of L-Ala13, D-Ser12-relacidine B **(4c)**

Table S9. ^{1}H-NMR (d_6-DMSO) characterization of D-Ala13, D-Ser12-relacidine B **(4d)**

Residue	-NH	Hα	Hβ	Hγ	Hδ	Hε
D-Ser1	7.88 (1H, d, *J* = 7.4 Hz)	4.27 (1H, m)	3.51 (2H, m)	5.15 (1H, br s)		
D-Tyr2	8.02 (1H, m)	4.35 (1H, m)	2.83 (1H, dd, *J* = 14.2, 4.3 Hz) & 2.62 (1H, m)	Phenol: 9.18 (1H, s), 6.90 (2H, d, *J* = 8.5 Hz), 6.57 (2H, d, *J* = 8.3 Hz)		
D-Trp3	8.01 (1H, m)	4.52 (1H, m)	3.16 (1H, m) & 2.95 (1H, m)	Indole: 10.80 (1H, s), 7.58 (1H, t, *J* = 7.3 Hz), 7.34 (1H, d, *J* = 8.1 Hz), 7.15 (1H, d, *J* = 2.4 Hz), 7.07 (1H, t, *J* = 7.5 Hz), 6.99 (1H, t, *J* = 7.4 Hz)		
D-Orn4	8.03 (1H, m)	4.38 (1H, m)	1.73 (1H, m) & 1.56 (1H, m)	1.56 (2H, m)	2.78 (2H, m)	7.75 (2H, br m)
Orn5	8.09 (1H, m)	4.41 (1H, m)	1.71 (1H, m) & 1.53 (1H, m)	1.53 (2H, m)	2.77 (2H, m)	7.75 (2H, br m)
Gly6	8.25 (1H, t, *J* = 5.6 Hz)	3.83 (1H, dd, *J* = 17.0, 6.0 Hz) & 3.73 (1H, m)				
D-Orn7	8.11 (1H, m)	4.38 (1H, m)	1.41 (1H, m) & 1.29 (1H, m)	1.33 (2H, m)	2.59 (2H, m)	7.67 (2H, br m)
Trp8	8.20 (1H, d, *J* = 8.1 Hz)	4,78 (1H, m)	3.12 (1H, dd, *J* = 14.6, 4.9 Hz) & 2.96 (1H, m)	Indole: 10.78 (1H, s), 7.58 (1H, t, *J* = 7.3 Hz), 7.31 (1H, d, *J* = 8.0 Hz), 7.10 (1H, d, *J* = 2.4 Hz), 7.04 (1H, t, *J* = 7.5 Hz), 6.95 (1H, t, *J* = 7.4 Hz)		
Thr9	8.04 (1H, m)	4.65 (1H, dd, *J* = 8.4, 4.0 Hz)	5.05 (1H, m)	1.07 (3H, d, *J* = 6.4 Hz)		
Ile10	8.11 (1H, m)	4.24 (1H, t, *J* = 7.8 Hz)	1.68 (1H, m)	1.52 (1H, m) & 1.13 (1H, m), 0.86 (3H, m)	0.86 (3H, m)	
Gly11	8.97 (1H, t, *J* = 5.4 Hz)	3.73 (1H, m) & 3.58 (1H, m)				
D-Ser12	7.42 (1H, d, *J* = 9.0 Hz)	4.27 (1H, m)	3.71 (2H, m)	4.82 (1H, br s)		
D-Ala13	7.50 (1H, d, *J* = 6.9 Hz)	4.08 (1H, p, *J* = 7.1 Hz)	1.39 (3H, d, *J* = 7.1 Hz)			
Lipid	2.12 (2H, m, O=CC**H₂**), 1.50 (2H, O=CCH₂C**H₂**), 1.27 (5H, -C**H₂CH**(CH)₃C**H₂**CH₃), 1.09 (2H, -C**H₂**CH₃), 0.82 (6H, -CH(C**H₃**)CH₂C**H₃**)					

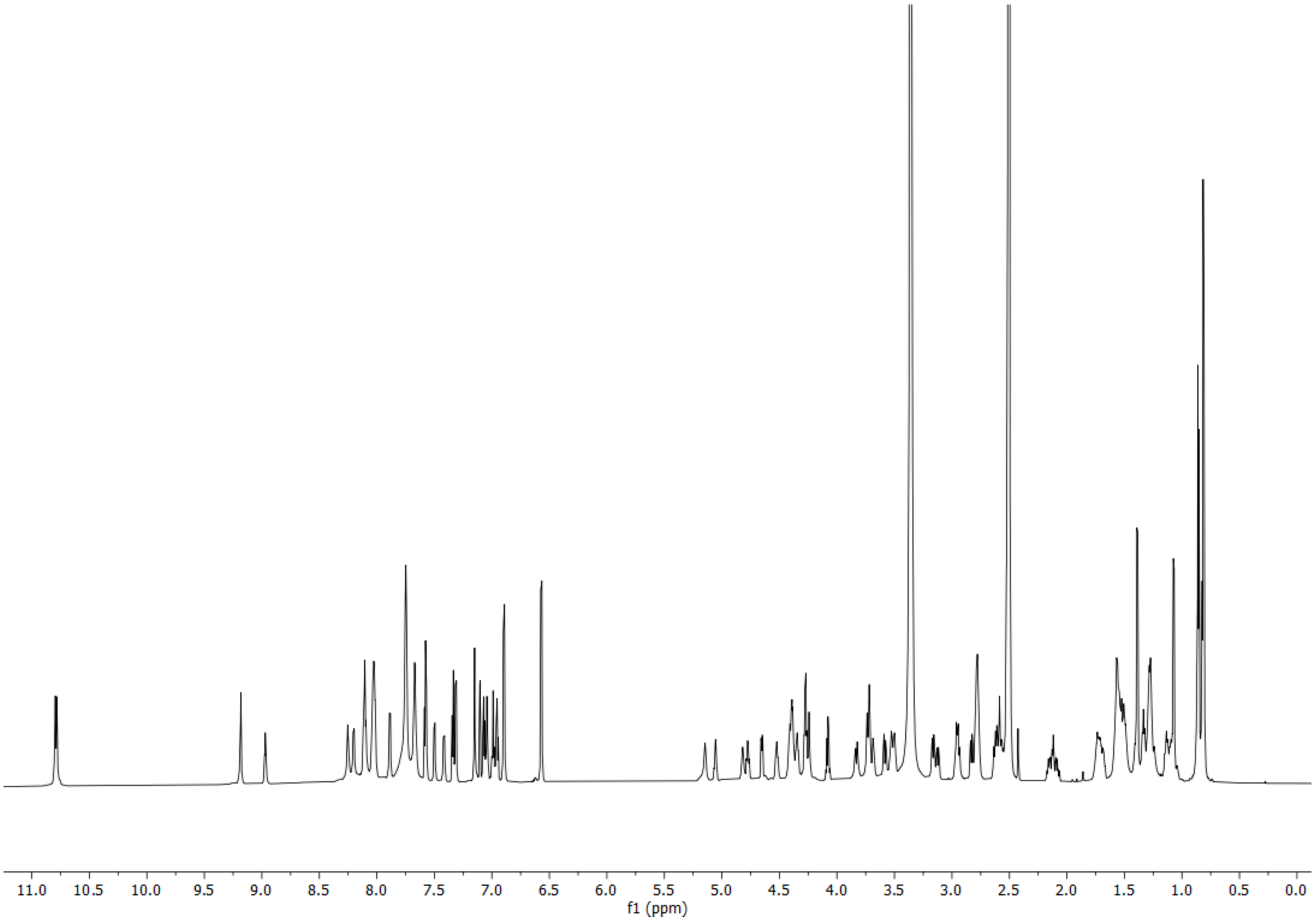

Figure S36. ^{1}H-NMR (d_6-DMSO) of D-Ala13, D-Ser12-relacidine B **(4d)**

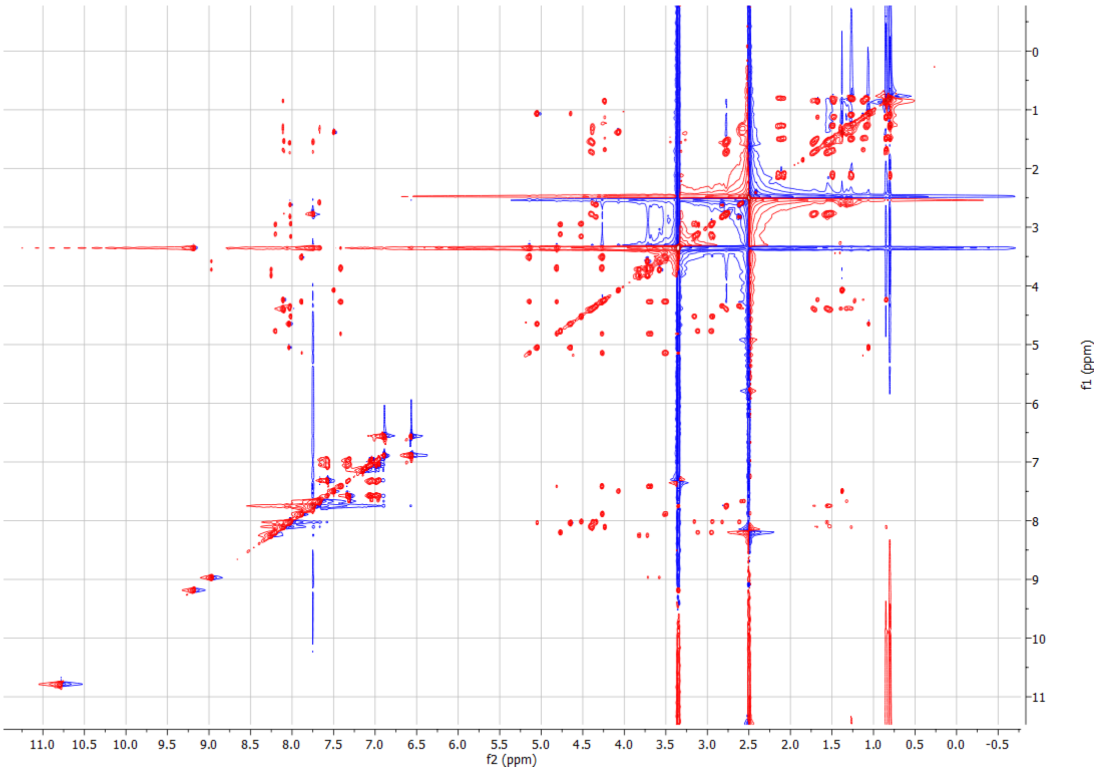

Figure S37. ^{1}H-^{1}H-TOCSY-NMR (d_6-DMSO) of D-Ala13, D-Ser12-relacidine B **(4d)**

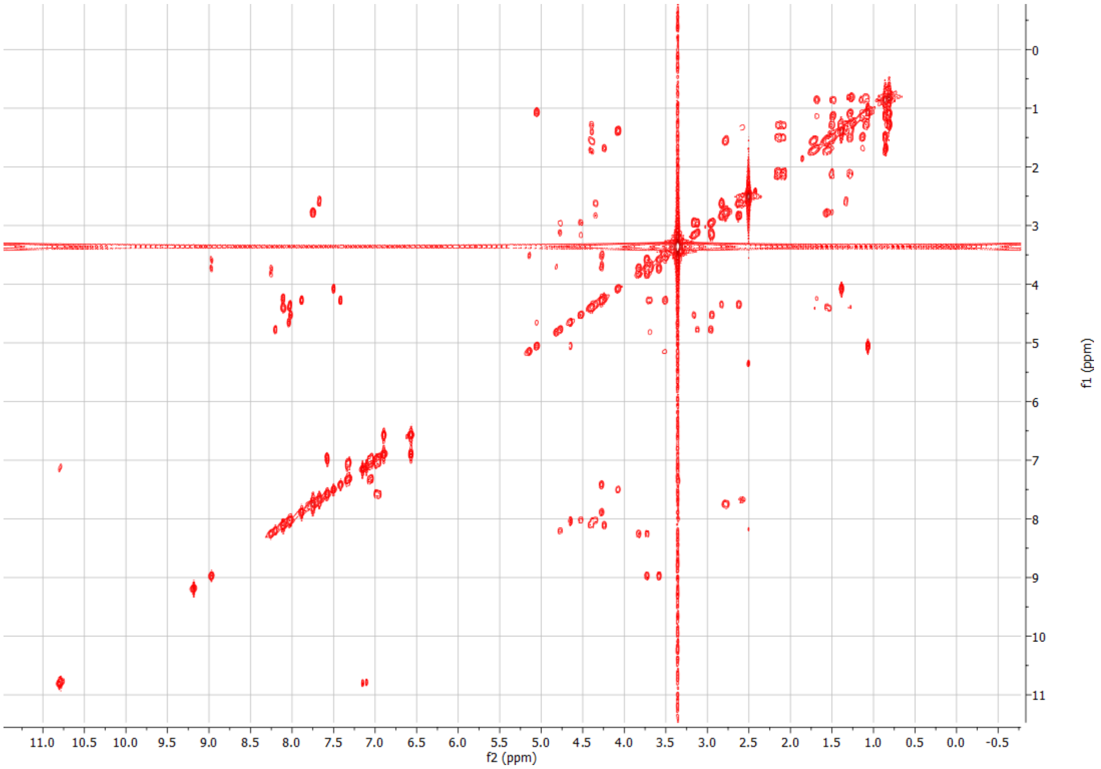

Figure S38. ^{1}H-^{1}H-COSY-NMR (d_6-DMSO) of D-Ala13, D-Ser12-relacidine B **(4d)**

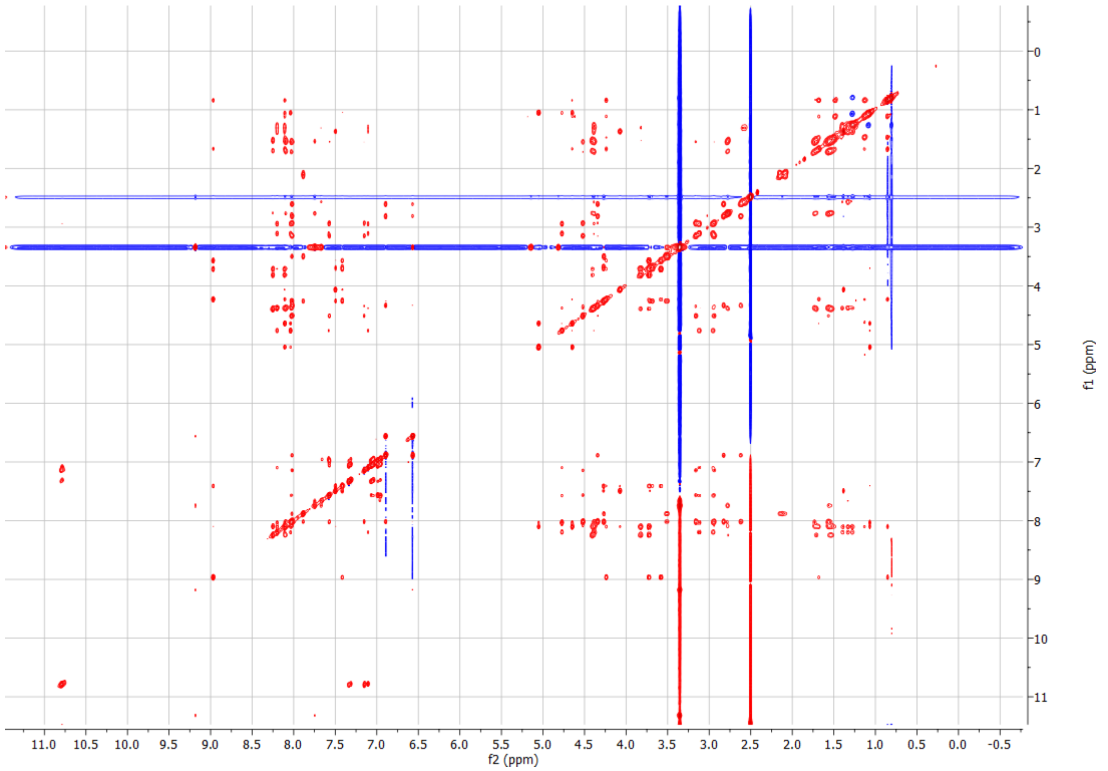

Figure S39. ^{1}H-^{1}H-NOESY-NMR (d_6-DMSO) of D-Ala13, D-Ser12-relacidine B **(4d)**

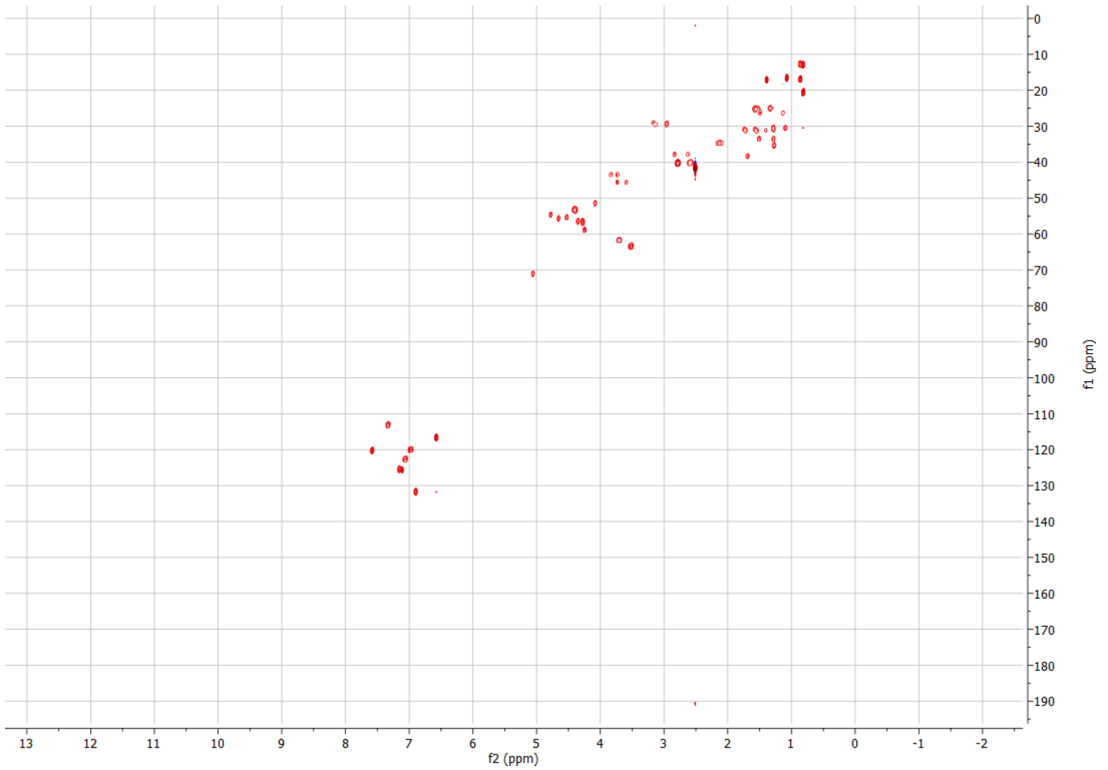

Figure S40. ^{13}C-^{1}H-HSQC-NMR (d_6-DMSO) D-Ala13, D-Ser12-Relacidine B **(4d)**

Table S10. ^{1}H-NMR (d_6-DMSO) characterization of relacidamide **(5)**

Residue	-NH	Hα	Hβ	Hγ	Hδ	Hε
D-Ser1	7.89 (1H, d, *J* = 7.4 Hz)	4.27 (1H, m)	3.51 (2H, m)	5.14 (1H, br s)		
D-Tyr2	8.02 (1H, m)	4.35 (1H, m)	2.83 (1H, dd, *J* = 14.3, 4.5 Hz) & 2.62 (1H, m)	Phenol: 9.18 (1H, s), 6.90 (2H, d, *J* = 8.5 Hz), 6.57 (2H, d, *J* = 8.4 Hz)		
D-Trp3	8.01 (1H, m)	4.52 (1H, m)	3.16 (1H, dd, *J* = 15.0, 4.5 Hz) & 2.95 (1H, dd, *J* = 14.9, 9.4 Hz)	Indole: 10.80 (1H, s), 7.60 (1H, d, *J* = 7.9 Hz), 7.34 (1H, d, *J* = 8.1 Hz), 7.15 (1H, d, *J* = 2.4 Hz), 7.07 (1H, t, *J* = 7.6 Hz), 6.99 (1H, t, *J* = 7.4 Hz)		
D-Orn4	8.03 (1H, m)	4.38 (1H, m)	1.74 (1H, m) & 1.57 (1H, m)	1.57 (2H, m)	2.78 (2H, m)	7.75 (2H, br m)
Orn5	8.09 (1H, d, *J* = 8.2 Hz)	4.41 (1H, m)	1.71 (1H, m) & 1.54 (1H, m)	1.54 (2H, m)	2.78 (2H, m)	7.75 (2H, br m)
Gly6	8.27 (1H, m)	3.80 (1H, dd, *J* = 16.8, 5.8 Hz) & 3.76 (1H, m)				
D-Orn7	8.07 (1H, d, *J* = 8.3 Hz)	4.33 (1H, m)	1.39 (1H, m) & 1.28 (1H, m)	1.32 (2H, m)	2.59 (2H, m)	7.66 (2H, br m)
Trp8	8.17 (1H, d, *J* = 8.3 Hz)	4,63 (1H, m)	3.10 (1H, m) & 2.90 (1H, dd, *J* = 14.7, 8.7 Hz)	Indole: 10.78 (1H, s), 7.58 (1H, d, *J* = 8.0 Hz), 7.31 (1H, d, *J* = 8.0 Hz), 7.10 (1H, d, *J* = 2.3 Hz), 7.04 (1H, t, *J* = 7.5 Hz), 6.97 (1H, t, *J* = 7.4 Hz)		
Dap9	8.22 (1H, d, *J* = 7.4 Hz)	4.27 (1H, m)	3.56 (1H, m) & 2.80 (1H, m)	6.83 (1H, t, *J* = 6.5 Hz)		
Ile10	8.27 (1H, m)	4.10 (1H, t, *J* = 9.7 Hz)	1.76 (1H, m)	1.56 (1H, m) & 1.16 (1H, m), 0.83 (3H, m)	0.84 (3H, t, *J* = 7.4 Hz)	
Gly11	9.02 (1H, t, *J* = 5.3 Hz)	3.95 (1H, dd, *J* = 14.8, 3.8 Hz) & 3.45 (1H, m)				
Ser12	9.00 (1H, d, *J* = 5.5 Hz)	3.91 (1H, m)	3.74 (1H, m) & 3.68 (1H, m)	5.07 (1H, br s)		
Gly13	7.95 (1H, m)	3.92 (1H, m) & 3.51 (1H, m)				
Lipid	2.12 (2H, m, O=CC$\mathbf{H_2}$), 1.50 (2H, O=CCH_2C$\mathbf{H_2}$), 1.27 (5H, -$\mathbf{CH_2CH}$(CH)$_3$$\mathbf{CH_2}$$CH_3$), 1.09 (2H, -$\mathbf{CH_2}$$CH_3$), 0.82 (6H, -CH($\mathbf{CH_3}$)$CH_2$$\mathbf{CH_3}$)					

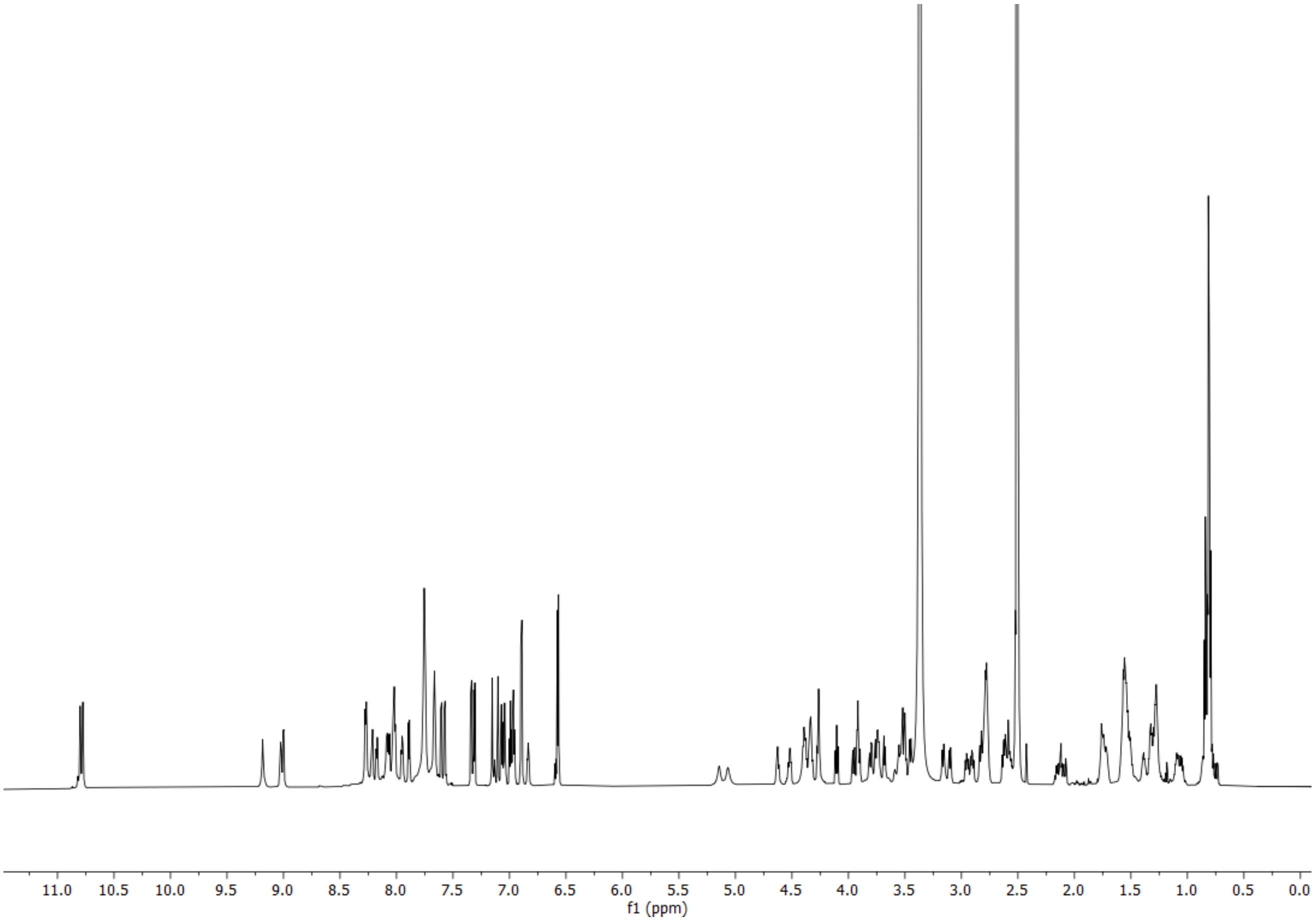

Figure S40. ^{1}H-NMR (d_6-DMSO) of relacidamide **(5)**

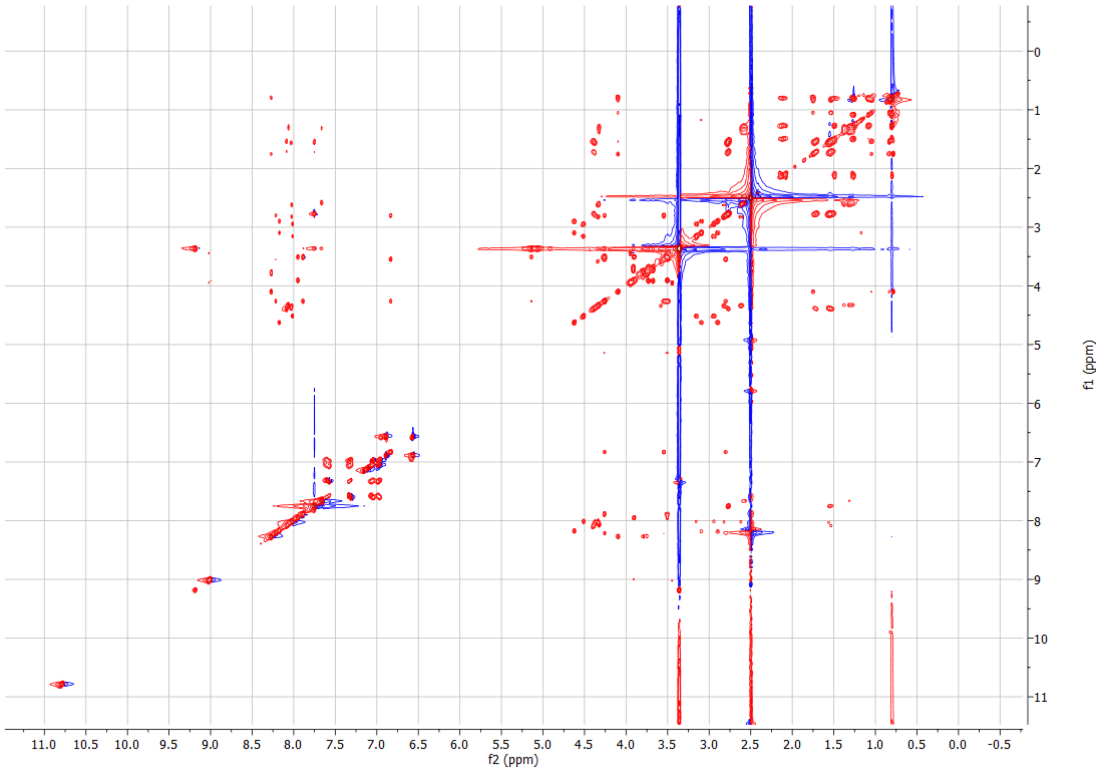

Figure S41. ^{1}H-^{1}H-TOCSY-NMR (d_6-DMSO) of relacidamide **(5)**

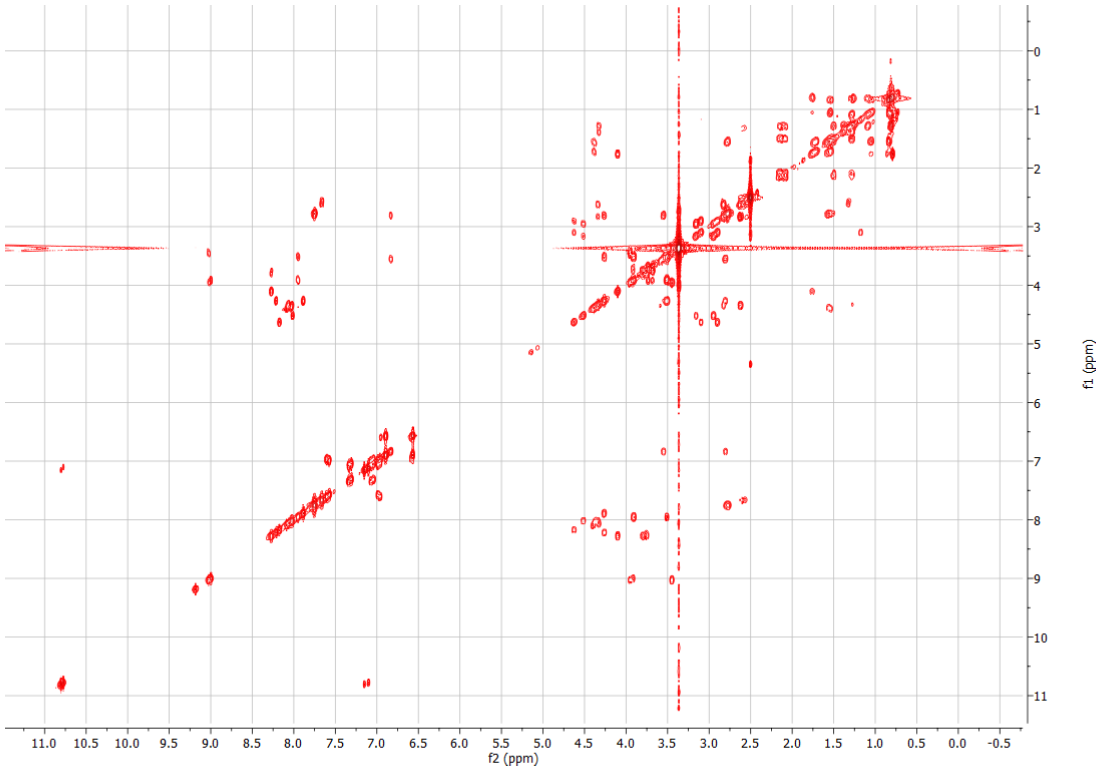

Figure S42. ^{1}H-^{1}H-COSY-NMR (d_6-DMSO) of relacidamide **(5)**

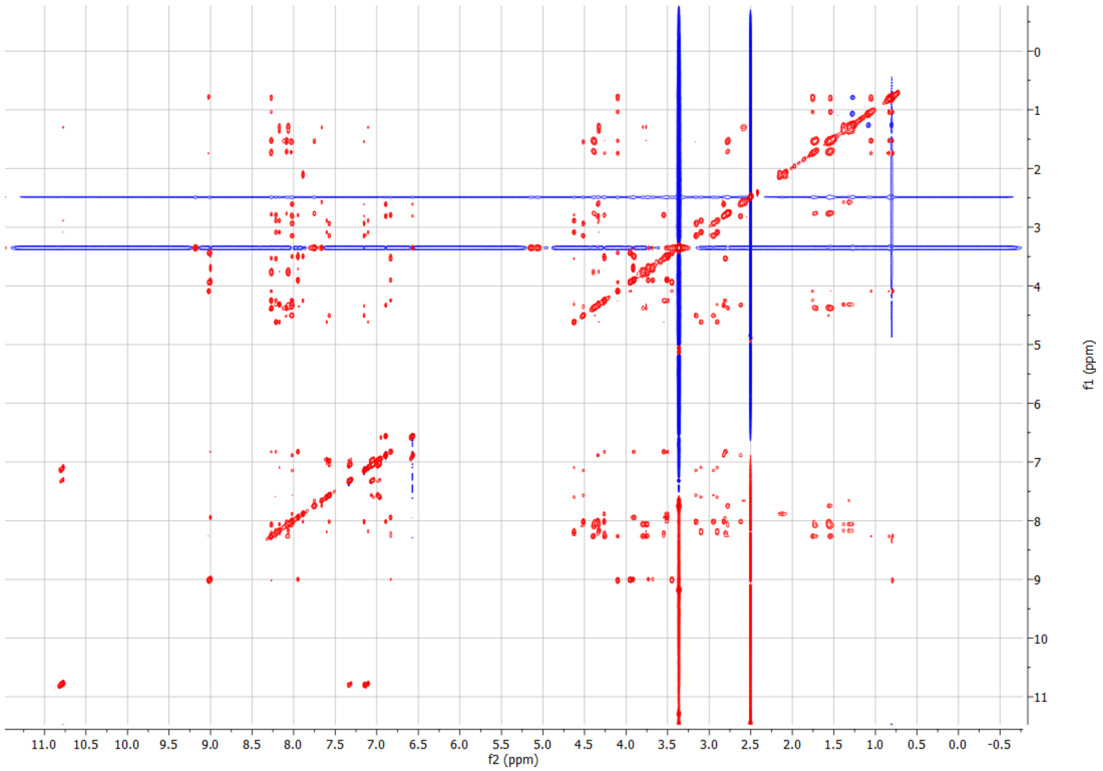

Figure S43. ^{1}H-^{1}H-NOESY-NMR (d_6-DMSO) of relacidamide **(5)**

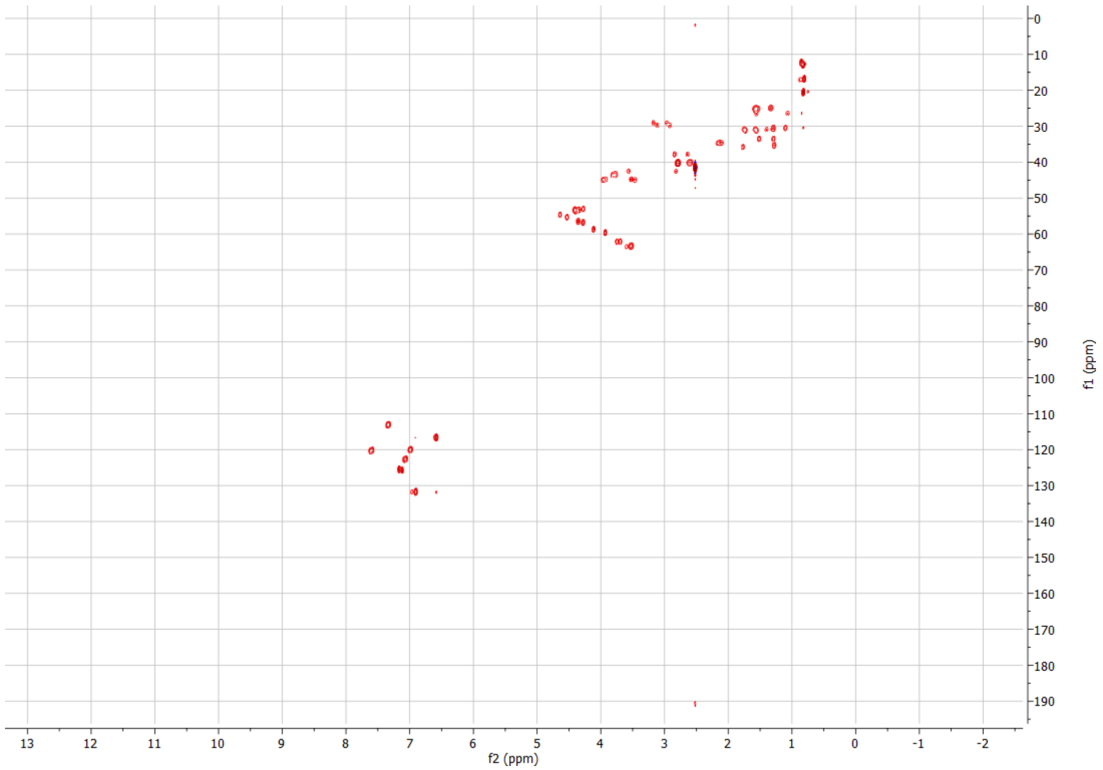

Figure S43. ^{13}C-^{1}H-HSQC-NMR (d_6-DMSO) of relacidamide **(5)**

XIV. References

1. Li, Z. *et al.* Characterization of two relacidines belonging to a novel class of circular lipopeptides that act against Gram-negative bacterial pathogens. *Environ. Microbiol.* **2020**, *22*, 5125–5136.
2. Mukherjee, S. & Van Der Donk, W. A. Mechanistic studies on the substrate-tolerant lanthipeptide synthetase ProcM. *J. Am. Chem. Soc.* **2014**, *136*, 10450–10459.
3. Dexter, H. L., Williams, H. E. L., Lewis, W. & Moody, C. J. Total Synthesis of the Post-translationally Modified Polyazole Peptide Antibiotic Goadsporin. *Angew. Chemie Int. Ed.* **2017**, *56*, 3069–3073.
4. Xiao, X. *et al.* Functional and Structural Insights into a Novel Promiscuous Ketoreductase of the Lugdunomycin Biosynthetic Pathway. *ACS Chem. Biol.* **2020**, *15*, 2529–2538.
5. Trebosc, V. *et al.* Dissecting Colistin Resistance Mechanisms in Extensively Drug-Resistant *Acinetobacter baumannii* Clinical Isolates. *mBio* **2019**, *10*, e01083-198.
